# Rational Computational Approaches in Drug Discovery: Potential Inhibitors for Allosteric Regulation of Mutant Isocitrate Dehydrogenase-1 Enzyme in Cancers

**DOI:** 10.3390/molecules28052315

**Published:** 2023-03-02

**Authors:** Masthan Thamim, Ashish Kumar Agrahari, Pawan Gupta, Krishnan Thirumoorthy

**Affiliations:** 1 Department of Chemistry, School of Advanced Sciences, Vellore Institute of Technology, Vellore 632014, Tamil Nadu, India; 2 Translational Health Science and Technology Institute, Faridabad 121001, Haryana, India; 3 Department of Pharmaceutical Chemistry, Shri Vile Parle Kelavani Mandal’s Institute of Pharmacy, Dhule 424001, Maharashtra, India

**Keywords:** chirality, oncometabolite, epigenetics, cancers, 2-Hydroxyglutarate, inhibitors, 3D-QSAR, molecular docking, molecular dynamics simulation, ADME, drug discovery, CADD

## Abstract

Mutations in homodimeric isocitrate dehydrogenase (IDH) enzymes at specific arginine residues result in the abnormal activity to overproduce *D*-2 hydroxyglutarate (*D*-2HG), which is often projected as solid oncometabolite in cancers and other disorders. As a result, depicting the potential inhibitor for *D*-2HG formation in mutant IDH enzymes is a challenging task in cancer research. The mutation in the cytosolic IDH1 enzyme at R132H, especially, may be associated with higher frequency of all types of cancers. So, the present work specifically focuses on the design and screening of allosteric site binders to the cytosolic mutant IDH1 enzyme. The 62 reported drug molecules were screened along with biological activity to identify the small molecular inhibitors using computer-aided drug design strategies. The designed molecules proposed in this work show better binding affinity, biological activity, bioavailability, and potency toward the inhibition of *D*-2HG formation compare to the reported drugs in the in silico approach.

## 1. Introduction

**Scheme 1:**
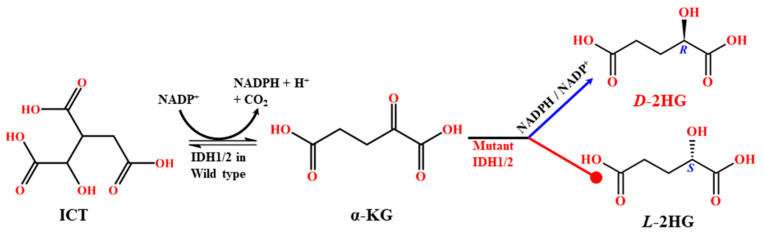
The mechanistic pathway of the wild-type and mutant-type IDH1/2 reaction in oncometabolite formation (Blue—allowed reaction, Red—forbidden reaction).

The wild-type cytosolic and mitochondrial homodimeric isocitrate dehydrogenase (IDH1/2) enzymes often catalyze the reversible oxidative decarboxylation of isocitrate into α-ketoglutarate (α-KG) using nicotinamide adenine dinucleotide phosphate (NADP^+^/NADPH) as a cofactor [1]. This biochemical reaction depicts the fundamental biotransformation reaction to maintain the post-translation modifications, DNA repair mechanism, cell signaling process, lipid synthesis, antioxidant formation, and control the redox potential [2,3,4]. Modification in the cellular metabolic process is a dynamic process for cancer progression [5]. Likewise, the alteration in enzyme nature is largely associated with many biological modifications including oncogene activation [6]. The mutations in IDH enzymes, especially, have an unexpected role in the genesis and progression of human malignancies [7]. Various clinical studies also state that the somatic point mutation in the mutant IDH (mIDH) enzymes causes a broad range of cancers [8]. Frequent experimental reports confirm that the mutations in IDH1/2 are the central grounds for gliomas [9], glioblastomas [10], medulloblastomas [11], acute myeloid leukemia [12], melanoma and sporadically in melanoma [13], intrahepatic cholangiocarcinoma [14], angioimmunoblastic T cell lymphoma [15], chondrosarcoma [16], prostate cancer [17], and sporadically in thyroid, breast, stomach, and pancreatic cancers and diseases including Ollier and Maffucci syndromes [7]. Mutations in IDH1 and 2 are the fundamental hallmarks of brain cancers and they are reported up to ≥80% in WHO grade II/III astrocytomas, oligodendrogliomas, glioblastomas, and oligoastrocytomas [18]. The mutations in IDH1/2 are somatic and heterozygous missense substituted, which are recognized to be playing a dominant role in gain of function over the wild-type enzyme. Intriguingly, the recurrent mutations in IDH1 and IDH2 occur exclusively at precise arginine residues to change a different amino acid in active sites of particular hotspots namely, R100Q, R132H, R132S, R132C, R132G, and R132L in IDH1, and R140Q and R172K in IDH2. This dictates the distinct catalytic properties which activate the oncogene pathway [19]. With respect to IDH1, the most accepted variation is R132H, which contributes ≥89% of mutation and plays a paramount role in the mutation to pledge the cancer progression extensively. All the remaining disparities are addressed at low frequencies and represented as passenger mutations [20]. The specific amino acid sequence R132 in IDH1 plays a prevailing role in the active site catalytic point of the isocitrate binding pocket for changing NADP^+^ into NADPH which yields α-KG [21]. The mutations of this enzyme change the amino acid sequence, resulting in the loss of the normal oxidative decarboxylation of isocitrate to α-KG process and leading to the gain of the neomorphic catalytic activity that reduces α-KG to an optically active endogenous oncometabolite 2HG in specific to *D* form as a product by consuming NADPH [22,23] (Scheme 1). Mutations in these enzymes harbor an exclusive gain of function to vary the normal cell route to produce specific enantiomer *D*-2HG [24], which is reported as an important biomarker in many cancers via the disruption of DNA and histone demethylation [25,26].

To date, huge numbers of mIDH1 inhibitors are reported worldwide, and most of the inhibitors are in the pre-clinical stage, except AG-120 and IDH-305. AG-120, also known as Ivosidenib, is a potential candidate for various IDH1 mutants and the only inhibitor which successfully entered into the phase III level of various clinical tests including Cholangiocarcinoma, Leukemia, Acute Myeloid Cancer, and solid tumors [27]. IDH-305 is also a potential candidate for the mIDH1 enzyme and has successfully entered the phase II level. Because of safety issues, its use was terminated for the clinical trials of mIDH1-associated Myeloid Leukemia, Grade II and Grade III gliomas, and neurological cancers [28,29]. Given that, it is important to continue designing small-molecular inhibitors or drugs that are active organic compounds modulating cellular pathways by targeting specific proteins, which have a low molecular weight and easy mobility in the biological environment. Small-molecular drug development is a challenging task that calls for a wide range of skills and several methodologies [30]. These small molecules can be obtained by target-based drug discovery via utilizing the structure and activity relationship, which typically entails target identification, target validation, assay development, hit identification, hit-to-lead, lead optimization, candidate selection, and later development. The exploration of the structure and activity relationship in detail yields potential, safe, and efficient inhibitors for drug discovery pathways [31]. In this direction, our underlying aim is to design a robust inhibitor to identify as a potential candidate to suppress the overproduction of *D*-2HG via the deactivation of the mIDH1 enzyme. Based on the literature review, 62 active reported inhibitors were identified [29,32,33,34], which specifically focus on the allosteric binding pocket of the mutant IDH1 enzyme.

Computer-aided drug discovery (CADD) tools are commonly used in drug discovery pathways which is a relatively lower-cost method to deliver potential drug candidates [35]. In CADD, the most commonly used methodology is the Quantitative Structure Activity Relationship (QSAR) which has been applied for decades in the development of relationships between physicochemical properties of chemical substances and their biological activities to obtain a well-grounded statistical model to design the potential drugs [36]. The three-dimensional QSAR (3D-QSAR) model is a statistical analysis method of the structure–activity relationship through the sequence of structural analogs to examine the relationship between the ligand and the protein [37]. Likewise, Molecular Docking is the foremost method in CADD used in a hit-to-lead optimization for drug discovery pathways to predict the binding energy conformations of the optimized ligand, which helps to realize the structural interaction between the ligand and its target protein [38]. The Molecular Dynamics (MD) simulation is the technique to describe the stability of the ligand and protein complex over time, under dynamic conditions [39]. Absorption, Distribution, Metabolism, and Excretion (ADME) are the fundamental properties to confirm the organic compound as the lead molecule in the biological condition via the pharmacokinetic properties such as drug-likeness, bioavailability, and synthetic accessibility [40]. So, the periodic combination of 3D-QSAR, Molecular Docking, MD simulation, and ADME studies is considered a standard method to investigate the binding nature between the ligand and its target receptor. In the present work, the research findings are systematically validated using 3D-QSAR, Molecular Docking, MD simulation, and ADME to figure out the potential inhibitors of the potent R132H in the mIDH enzyme at the allosteric site. To address our hypothesis, uniform analyses are performed to identify the hit molecules via the structure-activity relationship of Imidazole-Pyrimidine-Oxazolidin-based derivatives [29,32,33,34] as discussed earlier using 3D-QSAR modeling to build the effective predicted half maximal inhibitory concentration (pIC_50_) value and identify the required structural modification via the generated 3D contour maps. Based on the results, 3459 compounds are designed and shortlisted into 600 molecules to check their binding energy using molecular docking analysis and authenticate the 3D-QSAR results to discover the ligand–receptor interactions. Based on the docking results, the designed molecules are further subjected to MD simulation and ADME studies to validate the designed molecules that are potential candidates for the mutant IDH1 enzyme at the allosteric binding pocket.

## 2. Result and Discussion

### 2.1. 3D-QSAR

The fundamental principle underlying 3D-QSAR is that the difference in structural properties (3D-descriptors) is responsible for the variations in the biological activities (IC_50_) of the compounds; this leads to optimizing a series of compounds and identifying the target molecules. The result of the 3D-QSAR helps to enhance the specificity and potency of the small chemical moieties via altering the structural chemical changes of the compounds which enhances the affinity of the compounds for its target to preserve its original binding mode while forming additional and advantageous connections [36,41]. Based on these techniques and incorporated with the use of rational computational approaches, the neutralized reported compounds are split into a test set (17 compounds = 27.41%) and a training set (45 compounds = 72.59%) using the ratio of 70:30 to build a better Field-Based-3D-QSAR (FB-3D-QSAR) model. The FB-3D-QSAR is an advanced technique and is an execution of the CoMFA/CoMSIA methods with a specific set of parameters. It begins with the known activity of aligned compounds and predicts the biologically active or inactive ligands via steric, electrostatic, hydrophobic, and hydrogen bond donor and acceptor fields [42]. The resulting test set and a training set of reported molecules along with their biological activity and the parameters are shown in Table 1.

From the FB-3D-QSAR results, the obtained statistical data are well correlated and validated based on the references, as projected in Table S1 as the supporting information. The partial-least-square (PLS) factor 7 shows better results compared to all other factors where it contains minimal standard deviation (S.D = 0.1984), higher regression coefficient (R^2^ < 0.6 = 0.90), lower variance ratio, and root-mean-square error (P = 4.40 × 10^−17^ and RMSE = 0.71), and acceptable cross-validated correlation coefficient (Q2 < 0.5 = 0.5123) [43].

**Figure 1:**
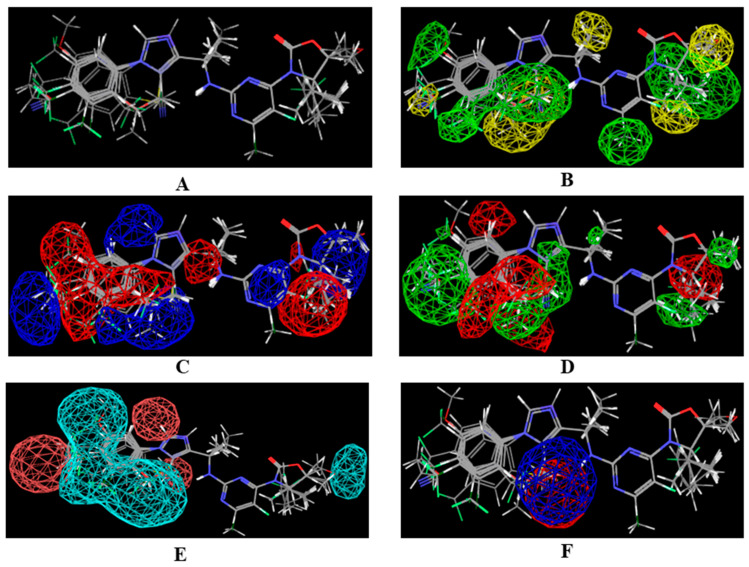
The aligned structures of (**A**) 62 reported ligands using Tanimoto similarities and the FB-3D-QSAR modeled results of favorable and unfavorable contour maps for (**B**) steric (Green—Positive, Yellow—Negative), (**C**) electrostatic (Blue—Positive, Red—Negative), (**D**) hydrophobic (Green—Positive, Red—Negative), (**E**) hydrogen bond acceptor (Cyan—Positive, Pink—Negative) and (**F**) donor (Blue—Positive, Red—Negative) properties.

The reference ligands are aligned via the Tanimoto similarities [44] and clustered [45] based on the related central nucleus with limited disparities, as shown in Figure 1, along with the physicochemical properties. The most biologically active compound is selected to modify the favorable functional node based on the R-group enumeration method [46] to design the potential candidate.

**Figure 2:**
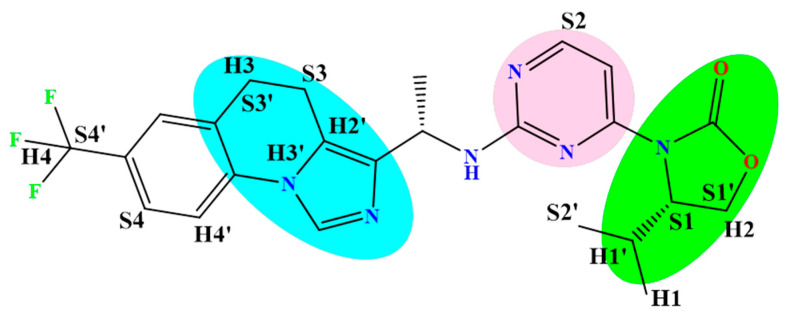
The steric (S and S′) and hydrophobic (H and H′) modification of favorable and non-favorable functional points in reference compound (RC-01). The green represents the oxazolidine moiety, pink represents the pyrimidine moiety, and cyan represents the imidazole moiety.

Among the five physiochemical properties, the steric and hydrophobic functions are playing a major role to determine and enhance the predicted biological activity of the designed compounds. Here, the steric function contributes roughly 45% and the hydrophobic factor contributes up to 29%, which is shown in Table S2 in the Supplementary Materials. Based on contour map analysis, the favorable modification in the selected molecules can result in improved activity via the functional group modifications. The most anticipated modifications of favorable and non-favorable functional groups for steric (S and S′) and hydrophobic (H and H′) properties are shown in Figure 2.

The most expected favorable and non-favorable modifications of steric and hydrophobic functional nodes are highly available in the oxazolidine, followed by imidazole and pyrimidine moieties. The favorable steric modification (S3) in the imidazole moiety can observe the steric and hydrophobic repulsion through the neighboring methyl group. To overcome this effect, the hydrophilic modification or non-favorable hydrophobic modification (H2′), is required. Likewise, the favorable (H3, H4, and S4) and non-favorable (H3′, S3′, H4′, and S4′) modifications of steric and hydrophobic functional groups enhance the activity of the designed compounds in a minimal range. Similarly, the favorable modification of steric and hydrophobic properties (S1, H1, and H2) in the oxazolidine moiety highly augments the activity of the designed compounds due to the free rotation away from the steric groups. The steric modification (S1), especially, will boost the activity of the designed compound; specifically, below the plane (dative bond), not above the plane, and it is incorporated in the ligand design. Likewise, the favorable hydrophobic (H2) modification will increase the activity only above the plane and not below. The non-favorable steric modification (S2′) is highly correlated with the favorable steric point (S2) of the pyrimidine moiety due to the pyrimidine–oxazolidine rotation (N-C), and the non-favorable hydrophobic modification (H1′) arises due to the availability of neighboring methyl group. The non-favorable steric modification S1′ avails above the plane to destabilize the oxazolidine moiety. From the FB-3D-QSAR, one can conclude that the small modification in the selected groups with specific functional nodes can dramatically increase the activity of the designed compound. Based on the contour map results, the high biological activity of molecule RC-01 was taken as the template molecule for further studies.

### 2.2. Molecular Docking

One of the most essential and widely used tools to deliver the potential drug candidate using low cost computer-assisted drug designing is molecular docking. The core of molecular docking is to place the small molecules in the active region of the target enzyme and use scoring functions to estimate a compound’s biological activity to predict the ligand accessibility in the biological environment. Molecular docking provides essential information about the three-dimensional structure possibilities of the specific ligand in the various regions of its target protein connectivity to list out via the scoring functions [47]. Based on this scoring value, one can determine the binding affinity of the ligand–protein interactions. From the FB-3D-QSAR results, the template molecule can allow modification with the selected nodes of the specific functional groups to check their binding affinity using the R-group enumeration method. Based on the available space in each functional node, it is possible to generate more than 2750 entries using the diverse R-group functionalities [46] such as steric effects, electronegativity, hydrophobicity, and hydrogen bond donor and acceptor nature. The limited entries were specifically selected based on the contour map results and designed 3459 ligands, especially using steric and hydrophobic functional nodes. A total of 936 designed molecules were selected and further allowed for the ligand preparation analysis [48] and finally, 600 compounds were shortlisted based on the highest predicted biological activity, which is further examined in the molecular docking study [49]. The molecular docking results are shown in Table S3 (reference compounds) and Table S4A,B (designed compounds) are in Supplementary Materials. Based on the molecular docking results, two ligands were chosen as reference compounds from the reported drugs, where one has the highest biological activity (RC-01) and another one contains the highest binding energy (RC-02), they are considered the control group to validate the newly designed compounds in this work. From the docking results, the top five designed ligands are selected for further studies (DC-01 to DC-05). The selected designed ligands and their parameters, along with the reference molecules, are shown in Table 2.

**Figure 3:**
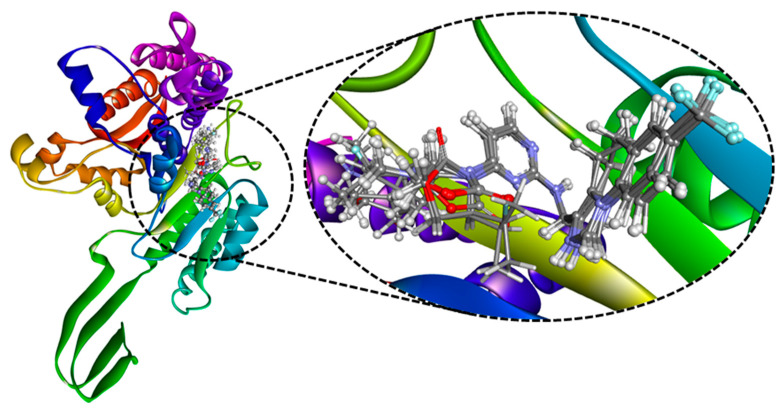
The cumulative picture representation of reference compound (stick model) and designed compounds (ball and stick model).

Based on the FB-3D-QSAR modeling, the specific steric active modification, especially in the fourth position of the oxazolidine moiety, results in the enhanced predicted biological activity which improves the binding affinity of the ligand–receptor interaction. The pyrrolidine, piperidine, and butyl functional groups enhance the steric nature (highlighted in pink), especially in DC-01 (*S*-3-fluoro-3-methylpyrrolidine), DC-02 (*R*-3-methylpyrrolidin-3-ol), DC-03 (3,3-dimethylpyrrolidine), DC-04 (piperidin-4-ol), and DC-05 (*S*-2,3-dimethylbutan-1-aminium). From the molecular docking results, it is very clear that the reference and designed ligands are perfectly fitted in the allosteric binding site and are shown in Figure 3. The 2D interactions of receptor–ligands obtained from the molecular docking results are shown in Figure S1A–G in Supplementary Materials.

From the molecular docking results, it is deduced that the functional node modification using R-group enumeration is the potential target to augment the hydrophobic interactions via pi–pi and alkyl–alkyl interactions due to the steric and hydrophobic functional group modifications. The reference ligands contain the least hydrophobic interaction in the oxazolidine moiety except for one alkyl–alkyl interaction with the VAL-281 residue. The main aim is to improve the activity of the designed compound without disturbing the existing interactions in the reference compounds via the FB-3D-QSAR approach. Thus, the modified ligands showcase the reference ligand interactions along with the enhanced interactions, especially in the oxazolidine site; this is one of the more robust predictions of the rational computational approach. The designed ligands are well enriched with hydrophobic and steric interactions in the preferable site to increase the activity and affinity. The molecular docking results of receptor–ligand interactions with specific residues of reference compounds along with designed compounds are shown in Table S5 in Supplementary Materials. The strong hydrophobic connectivity of selective residues ILE-113, ILE-117, TYR-285, MET-290, and CYS-379 enhance the alkyl–alkyl and pi–alkyl interactions to improve the binding affinity of the receptor–ligand complex. Similarly, the ILE-112, ILE-117, and ILE-118 residues involve a strong hydrogen bond, halogen-induced hydrogen bond (Fluorine-HB), and non-classical hydrogen bond interaction. Based on the molecular docking results, one can conclude that the high binding affinity molecules show higher interaction with their target protein, which is the potential key for robust drug delivery.

### 2.3. MD Simulation

Molecular dynamics (MD) simulation is the foremost method used and it creates a paradigm in computer-assisted drug discovery pathways to conclude if the designed compounds are biologically active or inactive. The fundamental principle of MD simulation is to analyze the physical movement of atoms in the molecule by using intermolecular interactions under dynamic conditions over a time period to provide abundant information such as enzymatic favorable reactions, chemical pathways, thermodynamic and kinetic stability, and so on. For the flair drug discovery identification, the designed compounds must fulfill all the conditions in the MD simulation study which allow interaction with its target protein under the biological condition over a period to achieve results confirming whether the specific compound is biologically stable or unstable [50]. The selected designed compounds DC (01 to 05) and reference compounds are further subjected to an MD-simulation study [51] to identify the stability of the receptor–ligand complex in the biological environment. The results depict the ligand stability through the root-mean-square deviation (RMSD), root-mean-square fluctuation (RMSF), the radius of gyration (Rg), and hydrogen bond (HB) plots, which all state that the designed compounds have better stability for 50 ns compared to the reference compounds.

#### 2.3.1. Root-Mean-Square Deviation (RMSD)

**Figure 4:**
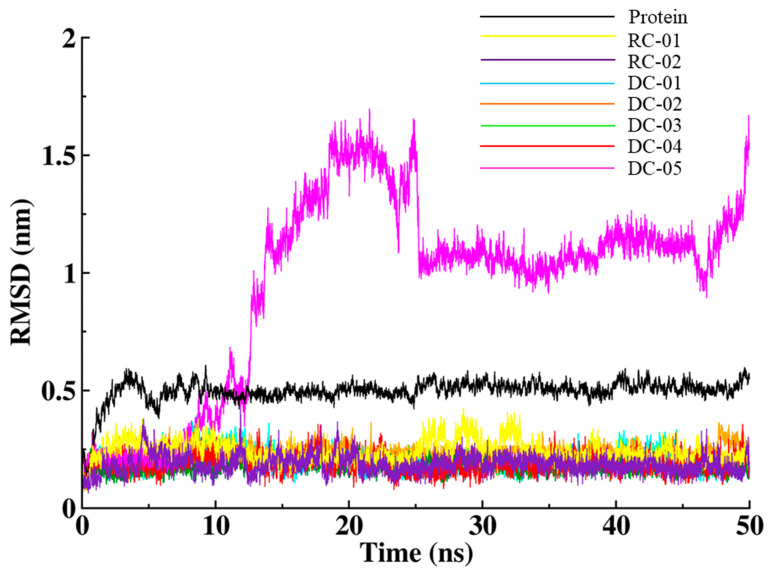
The overall RMSD analysis of protein and ligands compounds for 50 ns.

The RMSD of carbon alpha (C-alpha) of all the investigated systems was calculated to determine the convergence, i.e., stability of the trajectory. The average RMSD values of the designed compounds DC-03 (0.18 nm), and DC-04 (0.19 nm) are slightly lower than the reference compounds RC-01 (0.25 nm) and RC-02 (0.20 nm), which indicates that the designed complex is comparably more stable than the reference complex which is shown in Figure 4. However, the average RMSD value of DC-01 (0.20 nm) displayed similar stability compared to RC-02 and DC-02 (0.24 nm) and displayed higher stability than RC-01 and lower stability than RC-02. While the apo-protein average RMSD value is (0.50 nm) and the designed compound DC-05 (0.90 nm) shows an extremely high value, which indicates that it is unstable in the protein–ligand environment. As the DC-05 complex system is unstable, it is excluded from further studies.

#### 2.3.2. Root-Mean-Square Fluctuation (RMSF)

**Figure 5:**
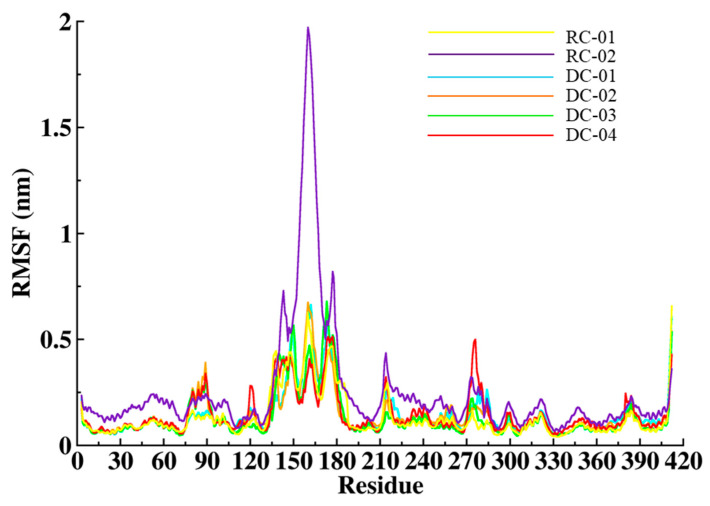
The overall RMSF analysis of designed and reference compounds.

The inherent fluctuation of the receptor–ligand is frequently linked to its function. The residue-wise protein fluctuation factor is determined, which is directly related to the biological function of the specific ligand. In Figure 5, the overall RMSF value of the designed compound DC-01 shows a slightly high fluctuation in two specific regions (140–180 and 280–290) compared to the reference compound RC-01. Whereas, DC-02 shows identical fluctuation compared to RC-01, except it is in a different region (70–90). The overall RMSF value of DC-03 was considerably high in the zones of 70–90, 140–150, and 160–180 as compared to the RC-01. Similarly, DC-04 also shows an acceptable high fluctuation in specific regions (70–90, 110–120, and 270–280) as compared to the RC-01 where the average RMSF value of the RC-02 is highest among all the complex systems. This indicates that all of the designed compounds (DC-01 to DC-04) have comparable stability to the reference compounds RC-01 and RC-02.

#### 2.3.3. Radius of Gyration (Rg)

**Figure 6:**
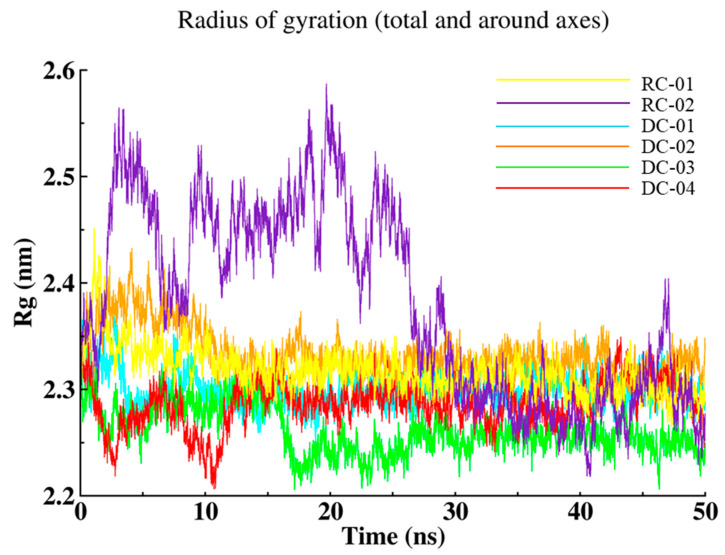
The overall Rg plot for designed and reference compounds for 50 ns.

The Rg is a very important parameter for measuring the structural compactness, overall folding, and shape of the protein. Figure 6 exhibits the Rg plot for all the complexes (DC and RC). The average Rg values of DC-01 (2.30 nm), DC-03 (2.26 nm), and DC-04 (2.28 nm) are lower than RC-01 (2.32 nm), which indicates that they have higher compactness, and DC-02 (2.33 nm) compactness is nearly similar to RC-01. The average Rg value of RC-02 is (2.36 nm) shows the least compactness in comparison to all other complexes, which designates that the designed compounds, DC-01 to DC-04, are much more stable and active in the biological environment compared to the reference compounds RC-01 and RC-02.

#### 2.3.4. Hydrogen Bond (HB) Interaction

**Figure 7:**
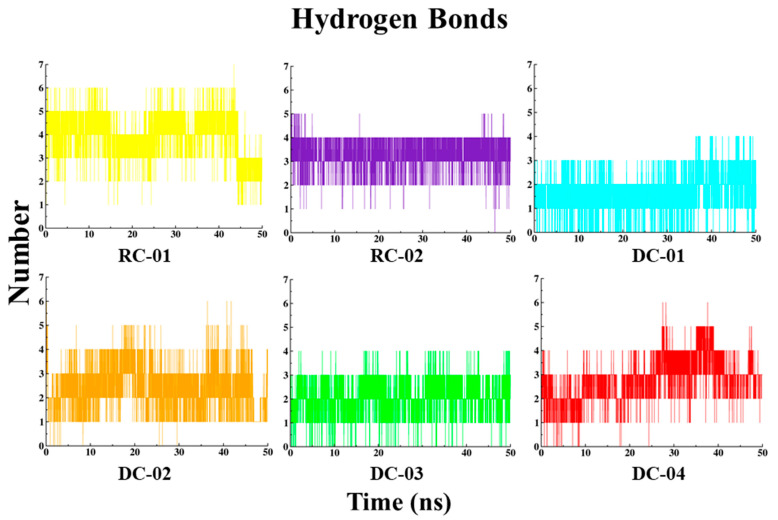
The HB interaction plot of the designed and reference compounds for 50 ns.

The HB interaction is very essential to determine the receptor–ligand interaction in dynamic conditions over time. Figure 7 shows the HB plot for all the investigated systems including reference and designed compounds. The average number of HB interactions for DC-01(1.833), DC-02(2.428), DC-03(2.164), and DC-04(2.901) are lower compare to RC-01(4.059) and RC-02(3.191) up to 50 ns. Comparably DC-02, DC-03, and DC-04 have acceptable HB average values but are not higher compared to the reference molecules. To overcome this consequence, the ligand–receptor interaction is analyzed at the final frame of the overall simulation (50 ns).

**Figure 8:**
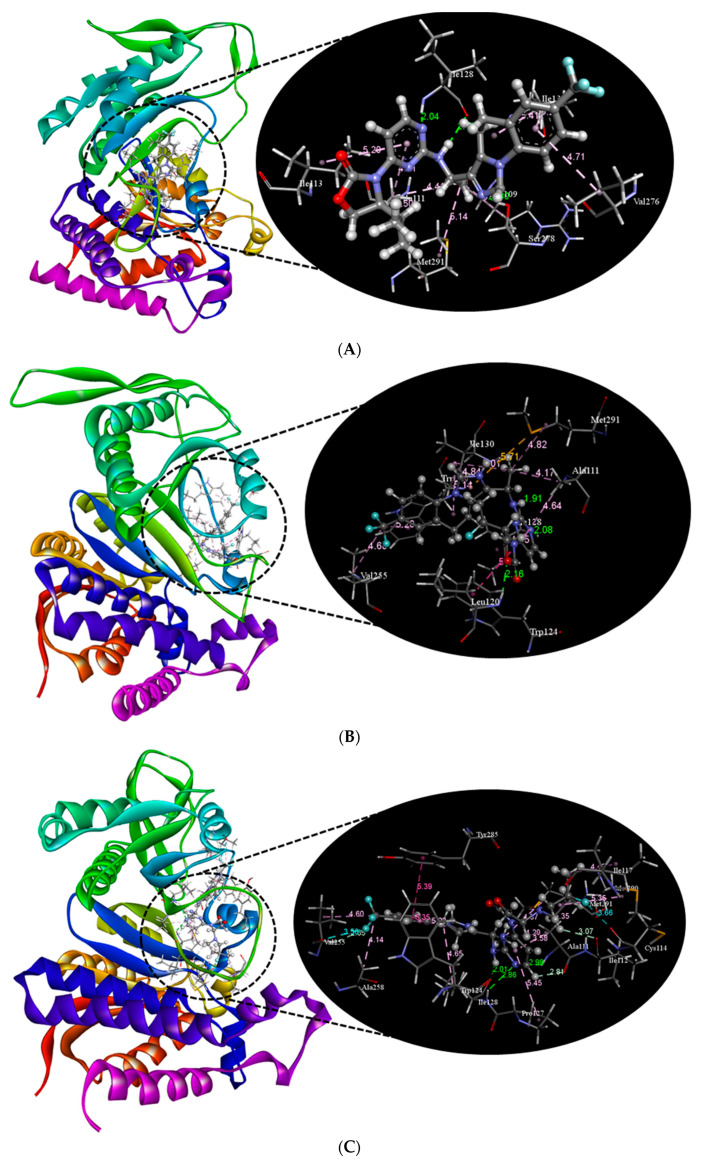

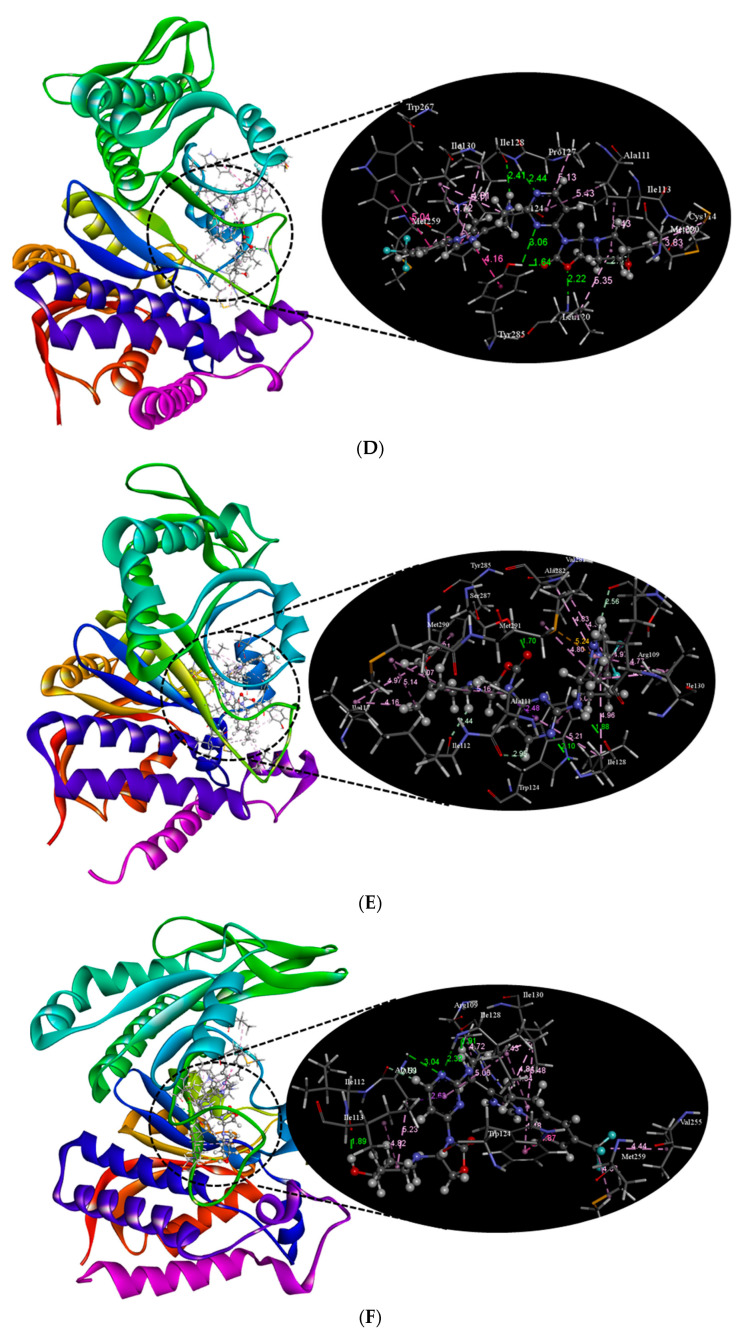
The receptor–ligand 3D interaction of reference compound (**A**) (RC-01), (**B**) (RC-02), and the designed compounds (**C**) (DC-01), (**D**) (DC-02), (**E**) (DC-03) and (**F**) (DC-04) after the simulation at the final frame (50 ns). Green—Classical and non-classical hydrogen bond interactions, Cyan—halogen induced hydrogen bond interactions, Lavender—alkyl-alkyl and pi-alkyl interactions, Magenta—pi-pi interactions.

Figure 8 shows the final frame (50 ns) of receptor–ligand interaction after the MD simulation using the Groningen Machine for Chemical Simulation (GROMACS v2022.4) package [51]. The 2D representation of receptor–ligand interaction after the MD simulation at the final frame (50 ns) is shown in Figure S2A–F in Supplementary Materials.

From the receptor–ligand interaction after the simulation at 50 ns as shown in Figure 8A–F, it is very clear that the designed ligands have better interaction compared to the reference compounds. The total number of residues involved in ligand–receptor interaction after the simulation is shown in Table 3.

Here, the reference compound RC-01 has 12 interactions including 9 hydrophobic (alkyl–alkyl and pi–alkyl) and 3 strong HBs with the help of eight active residues around the ligand. Similarly, RC-02 has a total of 13 interactions which include 8 hydrophobic (alkyl–alkyl, pi–alkyl,) 3 strong HBs, one pi–pi stacking, and pi–sulfur with the help of eight active residues around the ligand. However, the designed compounds contain a higher number of interactions over the reference compounds. DC-01 has a total of 20 interactions which include 3 classical and non-classical HBs, one halogen-induced HB (Fluorine-HB), 2 pi–pi stacking, and 11 hydrophobic interactions (alkyl–alkyl and pi–alkyl) with the help of 12 residues around the ligand pocket site. Similarly, DC-02 has 20 interactions which include 5 strong HBs, 3 pi–pi stacking, and 12 hydrophobic (alkyl–alkyl and pi-alkyl) with the help of 12 active residues. Furthermore, DC-03 contains 24 interactions which include 3 classical and non-classical HBs, one pi–sigma and pi–sulfur, and 16 hydrophobic (alkyl–alkyl and pi–alkyl) with the help of 14 active residues around the ligand pocket. DC-04 contains 18 interactions which include 4 strong classical HBs, one non-classical HB, one pi–sigma and pi–pi, and 11 hydrophobic (alkyl–alkyl and pi–alkyl) with the help of nine active residues. Among every designed compound, DC-03 had the highest number of interactions with 24, DC-01 and DC-02 had the same number of interactions with 20, and DC-04 had the lowest number of interactions with 18. Thus, all the designed compounds have a higher number of interactions compared to the reference compounds, RC-01(12) and RC-02(13). This dictates that our designed compounds are much more stable in the dynamic condition up to 50 ns.

### 2.4. ADME

A fundamental segment of drug discovery and development is addressing the pharmacokinetics and metabolism of the designed drug, which is often denoted as ADME properties [40]. The therapeutic effect of an effective drug is not only dependent on its biological activity, but also has to have proper ADME properties. Thus, it is very important to address the pharmacokinetic properties of the designed compound using in silico modeling to validate it as a therapeutic candidate. The ADME study in CADD tools is an essential and effective method to reduce the number of animal models in in vivo tests. The biologically active small molecules or drug candidates must follow the specific set of parameters which are widely denoted as pharmacokinetic properties. These biologically active designed molecules are called preclinical candidates and it is must have the affinity to be absorbed into the specific target surface, completely distributed to its target binding site, appropriately metabolized in the liver, and excreted after functioning. For this activity, a potent drug compound must follow Lipinski’s rule of five (Ro5) for excellent drug-likeness and absorption activity. The physicochemical properties on which Lipinski’s based drug-likeness comprise the number of H-bond donors (must be ≤5) and acceptors (must be ≤10), molecular weight (not more than 500 Da), and partition coefficient or lipophilicity as denoted as Log P (must be ≤5). Ligands, phytochemicals, and bioactive compounds derived from natural or synthetic sources or designed using CADD tools that would violate more than one of these rules are considered to have poor absorption [52]. The online free SwissADME server [53] is used to predict the drug bioavailability via the ADME property and confirm the designed drug molecules which obey Lipinski’s rule of five [54] in the biological environment. Overall, the ADME properties of our designed drugs along with reported drugs are shown in Table 4 with the required parameters.

From Table 4, one can conclude that our designed compounds have passed 90% in the drug-likeness test, and all have acceptable ranges except the MW, which is allowed in the drug discovery pathway [55]. Based on the results, the designed compound, DC-04 has highest solubility and PSA value, followed by DC-02, compared to all the other compounds. Similarly, DC-01 and DC-03 have higher PSA values and lower solubility compared to the reference compounds.

## 3. Conclusions

Abnormal activity in the mutated metabolic pathway leads to oncometabolite formation, which is the hallmark of cancers. Identifying potential drug candidates in cancer treatment is a challenging task for ongoing cancer research. The mIDH enzyme, especially, has gained huge attention in recent years to treat various cancers and other disease pathways. Specifically, the cytosolic mIDH1 enzyme gains the abnormal activity to be involved in the non-favorable reaction that produces the enantiomeric selective metabolite *D*-2HG. This metabolite is projected as a distinguished oncometabolite primarily in gliomas, glioblastomas, medulloblastomas, acute myeloid leukemia, melanoma and sporadically in melanoma, intrahepatic cholangiocarcinoma, angioimmunoblastic T cell lymphoma, and chondrosarcoma, and accountably in prostate, thyroid, breast, stomach, and pancreatic cancers and diseases including Ollier and Maffucci syndromes. The mutations in IDH enzymes are heterozygous somatic and recurrent modifications that take place in specific arginine residues to change the catalytic activity and endorse the abnormal activity involving the oncogene pathway. The R132H is the most accepted variation in the mIDH1 enzyme and it acts as a fuel to stimulate the oncogene pathway in more than 89% of human cancers and diseases. To date, the drug discovery pathway of the mIDH1 enzyme is quite complicated and only two inhibitors have been identified as potential candidates, namely, AG-120 and IDH-305. The AG-120 is the only inhibitor that successfully cleared phase III trials and is an accepted drug candidate for Cholangiocarcinoma, Leukemia, Acute Myeloid Cancer, and solid tumors; however, IDH-305 failed in phase III and the further developmental process is ongoing in IDH-305 modification. Examination of the combination of 3D-QSAR, molecular docking, MD simulation, and ADME studies yields the potential, safe, and efficient inhibitors for drug discovery pathways. This present work with the aid of CADD strategies aimed to identify the potential inhibitor for mIDH1, especially in the allosteric site to suppress the overproduction of *D*-2HG. Based on this direction, the Schrodinger suite 2022-3 was used to identify 62 allosteric site-reported inhibitors to build better FB-3D-QSAR modeling which obeys the required statistical parameters. From the FB-3D-QSAR method, the positive functional nodes are recognized and modified using the R-group enumeration method to design the robust derivatives of a highly active reference compound (RC-01) to predict biological activity. The designed compounds show high predicted biological activity compared to the reference compounds and selected designed compounds were further tested to understand the receptor–ligand interaction via molecular docking analysis to calculate the binding energy using the Glide package. The designed compounds DC-01 (−13.336 kcal/mol), DC-02 (−13.175 kcal/mol), DC-03 (−13.159 kcal/mol), DC-04 (−13.131 kcal/mol), and DC-05 (−12.952 kcal/mol) show high binding affinity compared to the reference compounds RC-01 (−11.800 kcal/mol) and RC-02 (−12.403 kcal/mol). These compounds were further tested to understand the stability via the MD simulation using the GROMACS v2022.4 package. From the MD simulation results, DC-01 to DC-04 show the highest stability compared to the reference compounds. The average RMSD value of DC-03 is 0.18 nm and DC-04 is 0.19 nm, which is a very low deviation compared to the reference compounds; RC-01 is 0.25 nm and RC-02 is 0.20 nm. Similarly, DC-01 has a value of 0.20 nm and DC-02 is 0.24 nm, which are an acceptable deviation as per the references. The designed compound DC-05 shows a high deviation in the RMSD of 0.90 nm compared to the apo-protein, which exhibits 0.50 nm. The RMSF plot of the designed compounds (except DC-05) shows acceptable fluctuation compared to the reference compound RC-01, where RC-02 shows higher fluctuation compared to all the other investigated systems. Similarly, the Rg plot shows very good compactness of designed compounds compared to the reference compounds. DC-01 has a value of 2.30 nm, DC-03 is 2.26 nm, and DC-04 is 2.28 nm and these are a higher compactness of ligands in the receptor region compared to RC-01 (2.32 nm) and RC-02 (2.36 nm). DC-02 has a value of 2.33 nm, which represents having similar and higher compactness compared to RC-01 and RC-02. The hydrogen bonding interaction analysis reveals that the average number of HBs is lower in all the designed compounds compared to the reference compounds. However, newly designed compounds are much augmented with hydrophobic and steric functional nodes. Thus, the types of interactions available in every compound after the simulation are explored, and surprisingly, our designed compounds have a higher number of interactions compared to the reference compounds, which drives the high stability over the reference compounds. The highest number of interactions observed for the investigated compounds after the simulation up to 50 ns is 24 in DC-03, followed by 20 in DC-01 and DC-02, 18 in DC-04, 12 in RC-01, and 13 in RC-02. The pharmacokinetic properties (ADME and Lipinski’s rule) are well correlated (except MW) to our designed compounds along with reference compounds. They exhibit pharmacokinetic, bioavailability, and drug-likeness properties. The results obtained from the rational computational approaches for the designed compounds show better stability, activity, bioavailability, and binding affinity compared to the reference compounds. This delineates that our designed compounds with in silico approaches using CADD tools could be potential candidates for an mIDH1 enzyme drug at the allosteric site to control the overproduction of *D*-2HG. The present work delineates the new strategies that would minimize the experimental cost involved in drug design and screening in laboratory research. This work provides new insights for researchers to synthesize the proposed class of molecules in the laboratory. Further, biochemical experimental investigations would be required to validate the designed compounds that could be therapeutic drug candidates for mIDH1 enzymes in various cancers.

## 4. Methodology

### 4.1. Data Set

The 62 specific allosteric binding inhibitors with similar molecular nuclei were taken from the existing literature and used in the present work. The 62 molecules showed a similar assay technique with a substantial disparity in their geometry and potency. The values of half maximal inhibitory concentration (IC_50_) varied from 49µM to 1nM, which were converted into pIC_50_ values using the following equation and converted into relatable values from 5.000 to 9.000.

$${\mathrm{pIC}}_{50}=-\log_{10}\left[{\mathrm{IC}}_{50}\right]$$

All the computational calculations were conducted using the Schrodinger suite 2022-3, which we acquired from www.schrodinger.com and used from 6 September 2022 to 27 September 2022 as a trial package, and the 3D geometries of the existing compounds were generated via the builder panel Maestro 13.3 [56] and consequently neutralized using Ligand preparation with Wizard segment [48]. We limited the reported ligand size to 500 atoms with default options such as desalt, determined the chirality from the 3D structure and disabled the tautomer generation, and executed the energy neutralization using the OPLS-2005 force field [57] for better results.

### 4.2. 3D-QSAR

The optimized compounds were treated in fingerprint analysis using Canvas similarities [45] and clustered to identify the mismatched molecular nucleus using Tanimoto analysis [44] to build a better 3D-QSAR model. We used the Field-Based (FB) 3D-QSAR techniques [42] to incorporate the 62 reported compounds into 70:30 ratios in the test set and training set for better results. We built the FB-3D-QSAR model using the Gaussian filed style and unchecked the use of input partial charges to generate electrostatic interactions. The pIC_50_ values of mIDH1 were used as the dependent variables and generated 3D molecular field descriptors were used as independent variables. Partial-least-square (PLS) procedure was used to correlate linearly in the FB-3D-QSAR method to limit the maximum PLS factor up to 7 to avoid over-fitting and Leave-one-out (LOO) cross-validation method is used to determine the cross-validation coefficient (R^2^), correlation coefficient (Q^2^), root-mean-square error (RMSE), the standard deviation of the regression (SD), and Pearson coefficient (Pearson-R). From these results, we explored the 5 physicochemical properties namely Gaussian Steric (GS), Gaussian Electrostatic (GE), Gaussian Hydrophobic (GH), Gaussian Hydrogen Bond Acceptor (GHA), and Gaussian Hydrogen Bond Donor (GHD) fields, which were visualized in the QSAR visualization module.

### 4.3. Molecular Docking

The Protein Preparation Wizard [58] in Maestro 13.3 was used to prepare the crystal structure of mIDH1 enzyme complex with IDH-305 ligand (PDB: 6B0Z). This was obtained from the RCSB protein data bank to remove water and heteroatoms, add hydrogen and partial charges, assign protonated state at pH 7.0 ± 2.0 using Epik mode [59], fill the missing loops and side chains using Prime [60], optimize with appropriate pH, and finally, minimize the energy of the complex using OPLS4 force field [61]. Then we created the grid box size 10Å × 10Å × 10Å (x, y, z coordinates 27.16, 25.74, 29.27) to delineate the binding site of mIDH1 using the ligand position using the Receptor Grid Generation module in Maestro13.3 with default settings. All the ligands were optimized with the aforementioned neutralization methodology to generate an ionization state at pH 7.0 ± 2.0 in Epik mode [59] with the OPLS-2005 force field [57]. Finally, we performed molecular docking using Glide version 2022-3 [49] standard precision method with default settings. All the ligand–receptor interactions obtained from molecular docking were visualized with the help of the Discovery Studio—2021 package [62].

### 4.4. MD Simulation

To examine the essential residues interaction in ligand binding, we performed the MD simulation for mIDH1 protein and ligands based on the molecular docking results using the GROMACS v2022.4 package [51]. The highest biological activity and binding energy of reported compounds were taken as the reference compounds to test our designed compounds. The high binding affinity and predicted biological activity of selected protein–ligand complexes from the results were exported. They were then used to create the topology parameters and coordinate files of receptor and hit molecules using the CHARMM36 force field [63] in GROMACS and SwissParam [64], respectively. The docked protein–ligand complex was solvated using a transferable intermolecular potential 3 point (TIP3P) water solvation model [65] enclosed within the dodecahedron box of 1 Å and neutralized via sufficient numbers of Na^+^ and Cl^−^ ions added via the Monte Carlo ion-placing method [66]. Further, the energy minimization was performed by using the steepest descent approach by applying a maximum force of 10 kJ/mol to avoid steric hindrance up to 500 ps. Berendsen coupling [67] and the Parrinello–Rahman method [68,69] were employed to regulate temperature (NVT) and pressure (NPT) at 300 k and 1 bar, respectively, inside the box up to 100 ps. The overall simulation time was fixed as 50 ns for the reference and the designed complexes to determine the ligand–protein stability.

### 4.5. ADME

To address the pharmacokinetics and drug-likeness properties, we utilized the online free SwissADME [53] server to check the ADME properties along with the Lipinski rule of five to confirm the drug ability of our designed and reported compounds.

## Figures and Tables

**Table 1:** Test set and training set compounds with their parameters using FB-3D-QSAR method.

S.NO	Reported Compounds	QSAR Set	Reported Biological Activity	Predicted Biological Activity
RC-01	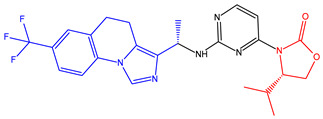	Test	9.000	7.960
RC-02	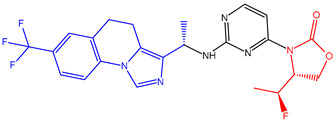	Training	8.398	8.068
RC-03	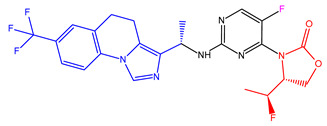	Training	8.678	8.208
RC-04	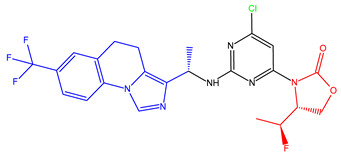	Test	8.674	8.379
RC-05	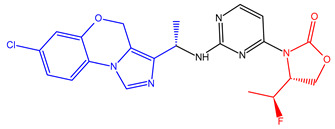	Training	8.658	8.634
RC-06	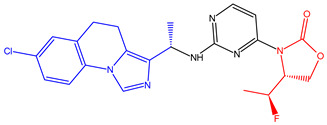	Training	8.638	8.113
RC-07	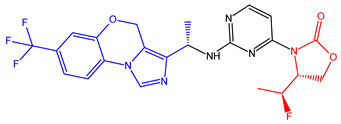	Training	8.569	8.573
RC-08	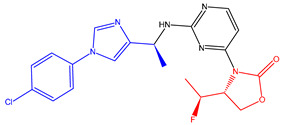	Test	8.398	7.945
RC-09	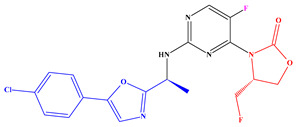	Test	8.301	7.414
RC-10	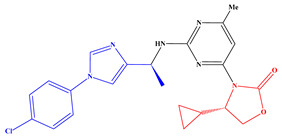	Training	8.097	8.054
RC-11	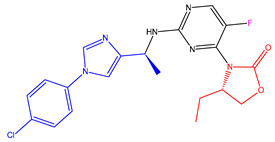	Training	7.886	8.105
RC-12	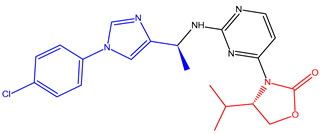	Training	7.824	7.850
RC-13	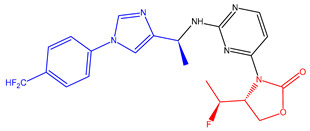	Test	7.824	7.511
RC-14	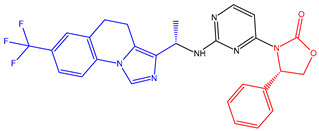	Test	7.810	8.263
RC-15	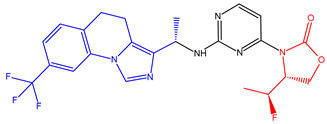	Training	7.735	7.873
RC-16	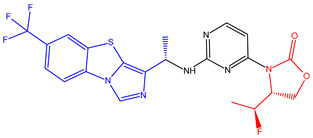	Training	7.717	7.687
RC-17	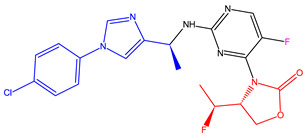	Training	7.658	8.097
RC-18	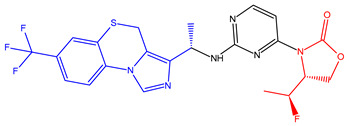	Training	7.623	7.902
RC-19	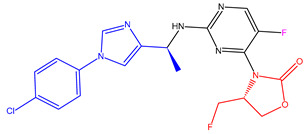	Training	7.620	7.487
RC-20	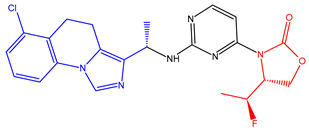	Test	7.618	7.527
RC-21	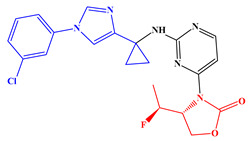	Test	7.602	7.205
RC-22	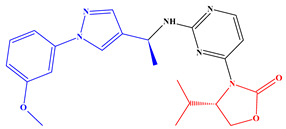	Training	7.585	7.636
RC-23	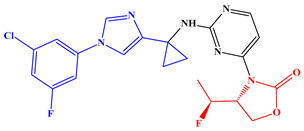	Training	7.569	7.410
RC-24	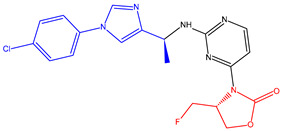	Training	7.523	7.335
RC-25	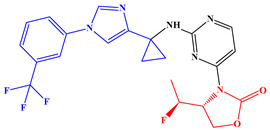	Test	7.495	6.904
RC-26	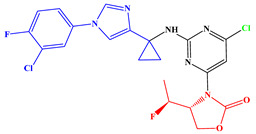	Training	7.495	7.505
RC-27	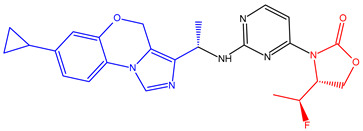	Training	7.476	7.554
RC-28	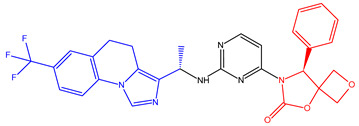	Training	7.471	7.599
RC-29	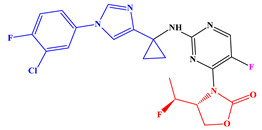	Training	7.456	7.358
RC-30	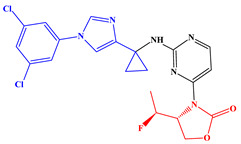	Training	7.444	7.325
RC-31	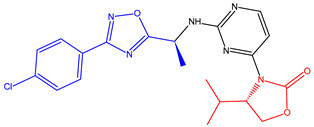	Training	7.444	7.481
RC-32	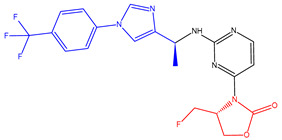	Training	7.409	7.420
RC-33	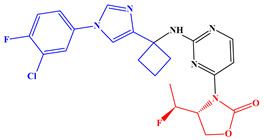	Training	7.398	7.231
RC-34	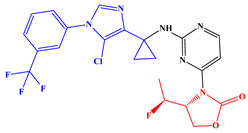	Training	7.387	7.371
RC-35	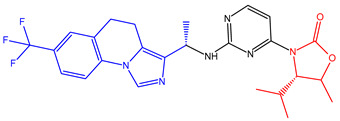	Training	7.377	7.439
RC-36	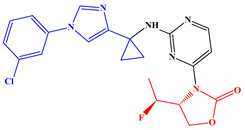	Training	7.377	7.248
RC-37	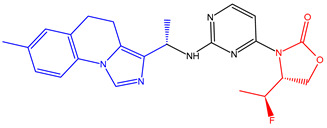	Training	7.370	7.590
RC-38	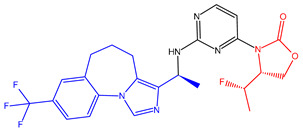	Training	7.354	7.414
RC-39	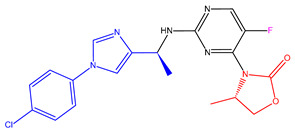	Training	7.328	7.366
RC-40	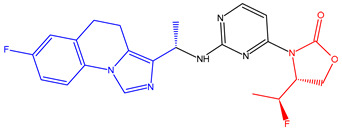	Training	7.321	7.482
RC-41	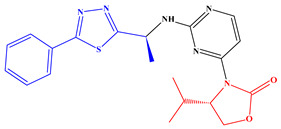	Training	7.310	7.202
RC-42	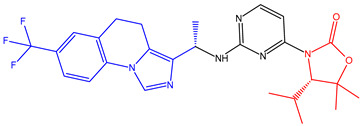	Training	7.294	7.447
RC-43	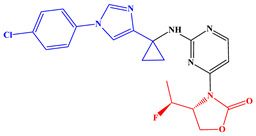	Training	7.268	7.351
RC-44	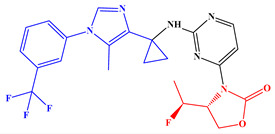	Test	7.252	7.044
RC-45	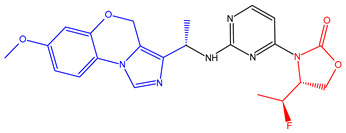	Training	7.208	7.281
RC-46	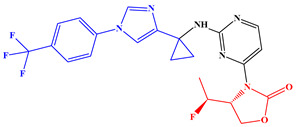	Training	7.187	7.457
RC-47	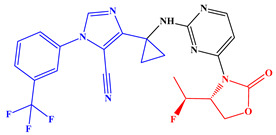	Training	7.071	6.963
RC-48	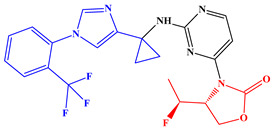	Training	6.951	9.967
RC-49	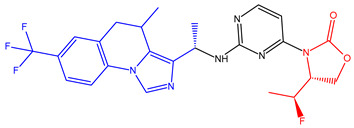	Training	6.922	6.937
RC-50	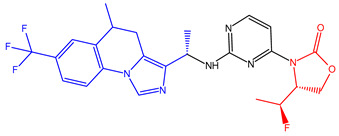	Training	6.891	6.956
RC-51	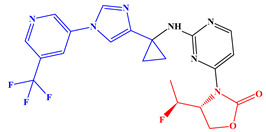	Training	6.818	6.872
RC-52	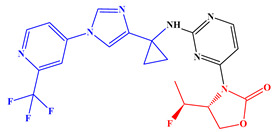	Test	6.658	6.398
RC-53	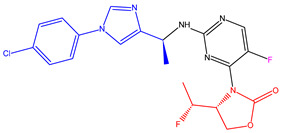	Test	6.620	8.192
RC-54	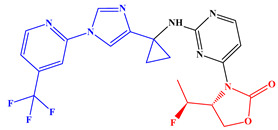	Test	6.602	6.843
RC-55	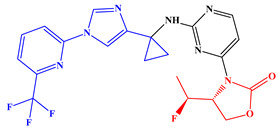	Test	6.575	7.394
RC-56	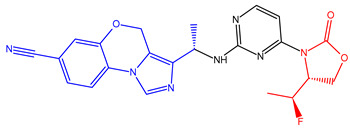	Training	6.320	6.070
RC-57	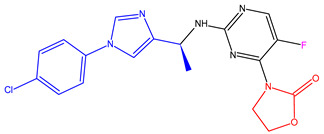	Training	6.252	6.442
RC-58	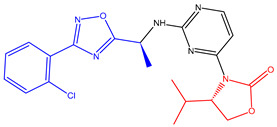	Test	6.215	6.964
RC-59	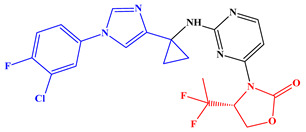	Training	6.142	6.224
RC-60	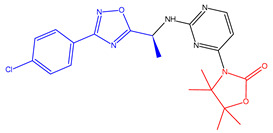	Training	6.086	5.996
RC-61	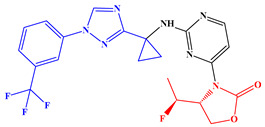	Test	6.080	7.028
RC-62	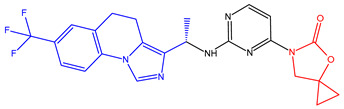	Test	5.000	5.909

**Table 2:** The selected designed and reference compounds along with their biological activity and docking scores.

S.NO	Active Ligands	Biological Activity	Docking Score kcal/mol	Glide Score kcal/mol
RC-01	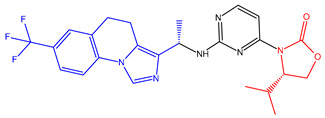	9.000 ^a^	−11.800	−12.988
RC-02	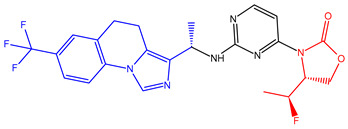	8.398 ^a^	−12.403	−12.497
DC-01	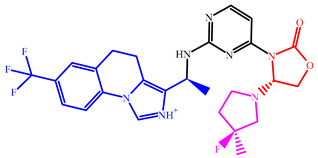	9.272 ^b^	−13.336	−13.424
DC-02	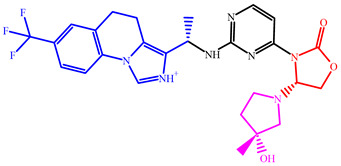	9.180 ^b^	−13.175	−13.270
DC-03	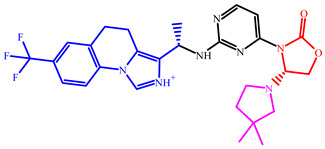	9.675 ^b^	−13.159	−13.256
DC-04	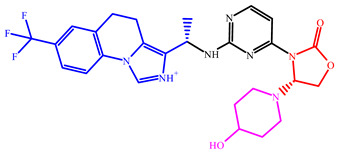	9.065 ^b^	−13.131	−13.218
DC-05	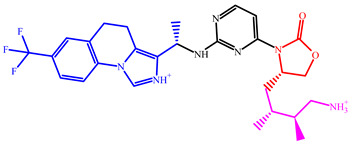	9.154 ^b^	−12.952	−13.045

a—Experimental value. b—Predicted value using FB-3D-QSAR.

**Table 3:** The ligand–receptor interactions of designed and reference compounds after the MD simulation at the final frame.

S.NO	Active Ligands	Hydrophobic Interactions				Hydrogen Bond Interactions			Total Number of Interactions
		alkyl–alkyl and pi–alkyl	pi–pi	pi–sigma	pi–sulfur	Classical	Non-Classical (Carbon–Hydrogen)	Halogen	
1	RC-01	ARG-109, ALA-111, ILE-113, ILE-130, VAL-276, MET-291	Nil	Nil	Nil	ILE-128, SER-278	ILE-128	Nil	12
2	RC-02	ALA-111, ILE-128, ILE-130, VAL-255, TRP-267	TRP-124	Nil	MET-291	LEU-120, ILE-128	Nil	Nil	13
3	DC-01	ALA-111, ILE-117, TRP-124, PRO-127, ILE-128, VAL-255, ALA-258, MET-290, MET-291	TRP-124, TYR-285	Nil	Nil	ALA-111, ILE-128	ALA-111, ILE-112, VAL-255	CYS-114	20
4	DC-02	ALA-111, ILE-113, CYS-114, LEU-120, TRP-124, PRO-127, ILE-128, ILE-130, MET-259, MET-290	TRP-124, TRP-267, TYR-285	Nil	Nil	LEU-120, ILE-128, TYR-285	Nil	Nil	20
5	DC-03	ARG-109, ALA-111, ILE-117, TRP-124, ILE-128, ILE-130, VAL-281, ALA-282, TYR-285, MET-290, MET-291	Nil	ALA-111	MET-291	ILE-128, SER-287,	ALA-111, ILE-112, SER-278	Nil	24
6	DC-04	ARG-109, ALA-111, ILE-113, TRP-124, ILE-128, ILE-130, VAL-255, MET-259	TRP-124	ALA-111	Nil	ALA-111, ILE-112, ILE-128	ALA-111	Nil	18

**Table 4:** ADME properties of the designed drugs along with reference drugs.

S.NO	Active Compounds	Lipinski’s Rule of Five					
		PSA Å	MW g/mol	Log P o/w	NRB	HBA	HBD
1.	RC-01	85.17	486.49	3.73	6	8	1
2.	RC-02	85.17	468.49	3.73	6	8	1
3.	DC-01	89.66	546.54	3.93	6	9	2
4.	DC-02	109.89	544.55	3.57	6	9	3
5.	DC-03	89.66	542.58	3.77	6	8	2
6.	DC-04	109.89	544.55	3.42	6	9	3

## Data Availability

The data presented in this study are available in supplementary material.

## Supplementary Materials

The following supporting information can be downloaded at https://www.mdpi.com/article/10.3390/molecules28052315/s1, Figure S1A: The molecular docking results for reference compound (RC-01) interaction with the receptor; Figure S1B: The molecular docking results for reference compound (RC-02) interaction with the receptor; Figure S1C: The molecular docking results for designed compound (DC-01) interaction with the receptor; Figure S1D: The molecular docking results for designed compound (DC-02) interaction with the receptor; Figure S1E: The molecular docking results for designed compound (DC-03) interaction with the receptor; Figure S1F: The molecular docking results for designed compound (DC-04) interaction with the receptor; Figure S1G: The molecular docking results for designed compound (DC-05) interaction with the receptor; Figure S2A: The MD simulation results for reference compound (RC-01) interaction with the receptor at the final frame (50 ns); Figure S2B: The MD simulation results for reference compound (RC-02) interaction with the receptor at the final frame (50 ns); Figure S2C: The MD simulation results for designed compound (DC-01) interaction with the receptor at the final frame (50 ns); Figure S2D: The MD simulation results for designed compound (DC-02) interaction with the receptor at the final frame (50 ns); Figure S2E: The MD simulation results for designed compound (DC-03) interaction with the receptor at the final frame (50 ns); Figure S2F: The MD simulation results for designed compound (DC-04) interaction with the receptor at the final frame (50 ns); Table S1: The PLS statistical data of the homogenous 3D-QSAR model using the Field-Based method; Table S2: The physiochemical property contribution of reported compounds using the FB-3D-QSAR method; Table S3: The molecular docking results for 62 reported compounds using Glide package in Schrodinger suite; Table S4: The molecular docking results for 229 designed compounds using Glide package in Schrodinger suite; Table S4A: The molecular docking results for 371 designed compounds using Glide package in Schrodinger suite; Table S5: The specific residues involved in the receptor-ligand interaction of reference and designed compounds using molecular docking analysis.

## Sample Availability

Samples of the compounds are not available from the authors.
