# Supplementary material for: Rational Computational Approaches in Drug Discovery: Potential Inhibitors for Allosteric Regulation of Mutant Isocitrate Dehydrogenase-1 Enzyme in Cancers
Source: Molecules. 2023 Mar 2;28(5):2315. doi: 10.3390/molecules28052315 (PMC10005488; doi:10.3390/molecules28052315)
Supplement: Supplementary file 1 [file molecules-28-02315-s001.zip › molecules-2193662-supplementary.pdf]

Supplementary Information Table of Contents

Table of Contents...2-3

List of Figures…... 4-10

List of Tables…... 11-42

1. Figures

Figures No	Figures Title	Page No
S1-A	The molecular docking results for reference compound (RC-01) interaction with the receptor.	4
S1-B	The molecular docking results for reference compound (RC-02) interaction with the receptor.	4
S1-C	The molecular docking results for designed compound (DC-01) interaction with the receptor.	5
S1-D	The molecular docking results for designed compound (DC-02) interaction with the receptor.	6
S1-E	The molecular docking results for designed compound (DC-03) interaction with the receptor.	6

S1-F	The molecular docking results for designed compound (DC-04) interaction with the receptor.	7
S1-G	The molecular docking results for designed compound (DC-05) interaction with the receptor.	7
S2-A	The MD simulation results for reference compound (RC-01) interaction with the receptor at the final frame (50 ns).	8
S2-B	The MD simulation results for reference compound (RC-02) interaction with the receptor at the final frame (50 ns).	8
S2-C	The MD simulation results for designed compound (DC-01) interaction with the receptor at the final frame (50 ns).	9
S2-D	The MD simulation results for designed compound (DC-02) interaction with the receptor at the final frame (50 ns).	9
S2-E	The MD simulation results for designed compound (DC-03) interaction with the receptor at the final frame (50 ns).	10
S2-F	The MD simulation results for designed compound (DC-04) interaction with the receptor at the final frame (50 ns).	10

2. Tables

Tables No	Tables Title	Page No
S1	The PLS statistical data of the homogenous 3D-QSAR model using the Field-Based method.	11
S2	The physiochemical property contribution of reported compounds using the FB-3D-QSAR method.	11
S3	The molecular docking results for 62 reported compounds using Glide package in Schrodinger suite.	11-14

S4	The molecular docking results for 229 designed compounds using Glide package in Schrodinger suite.	14-24
S4-A	The molecular docking results for 371 designed compounds using Glide package in Schrodinger suite.	25-41
S5	The specific residues involved in the receptor-ligand interaction of reference and designed compounds using molecular docking analysis.	41-42

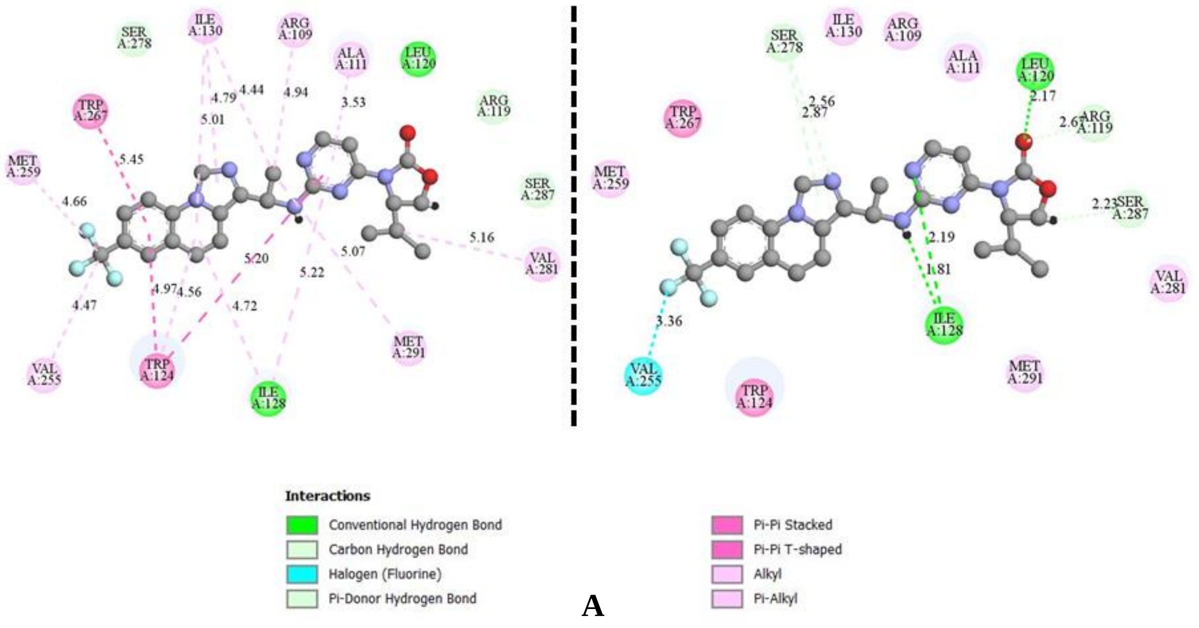

Figure S1-A: The molecular docking results for reference compound (RC-01) interaction with the receptor.

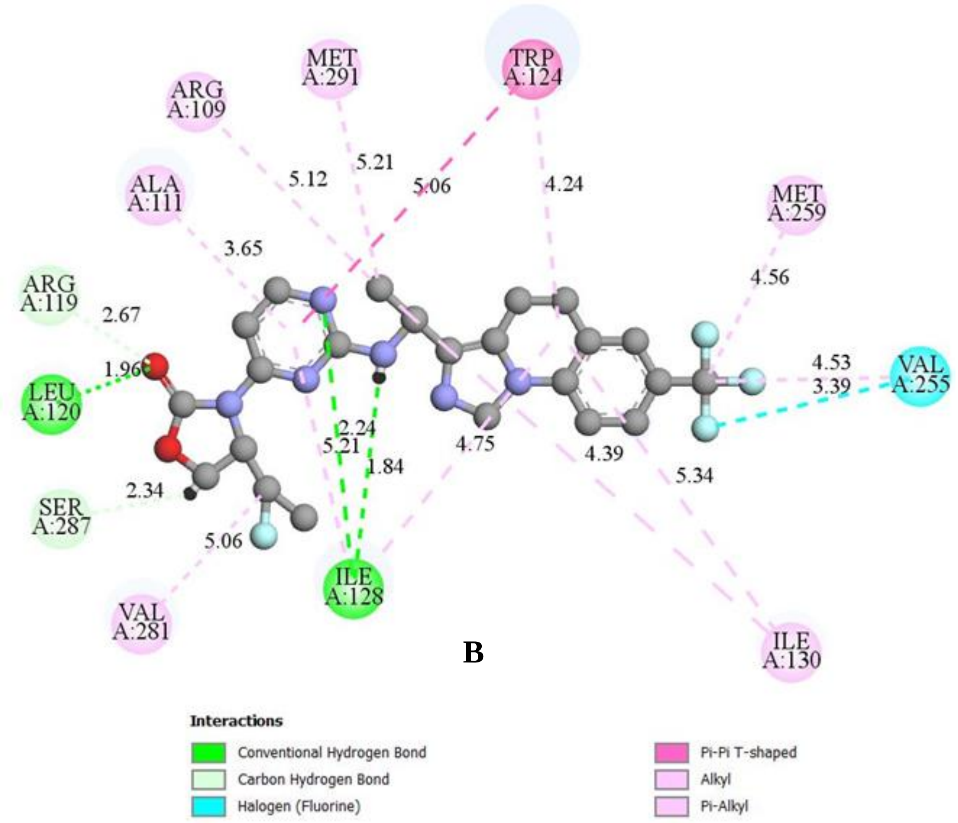

Figure S1-B: The molecular docking results for reference compound (RC-02) interaction with the receptor.

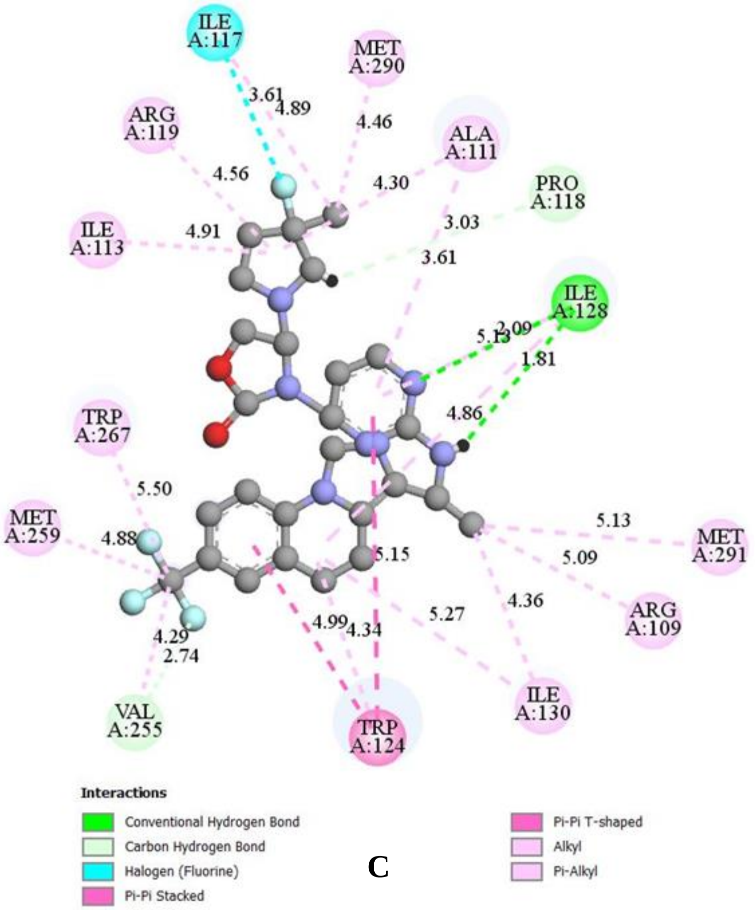

C

Figure S1-C: The molecular docking results for the designed compound (DC-01) interaction with the receptor.

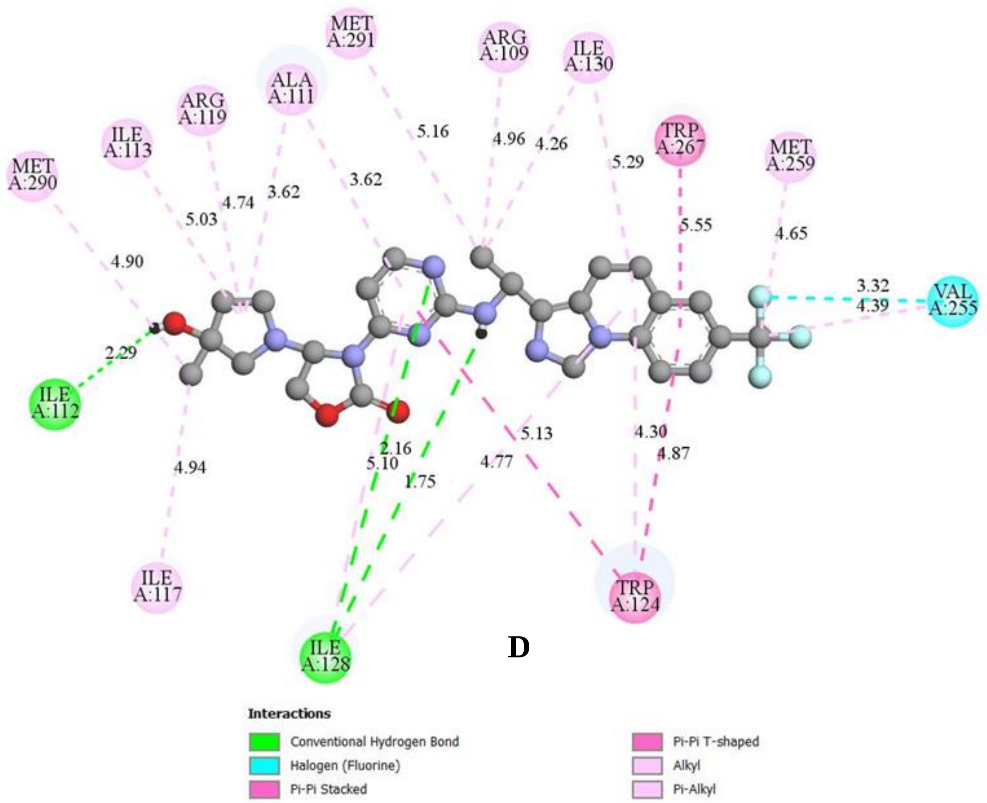

D

Figure S1-D: The molecular docking results for the designed compound (DC-02) interaction with the receptor.

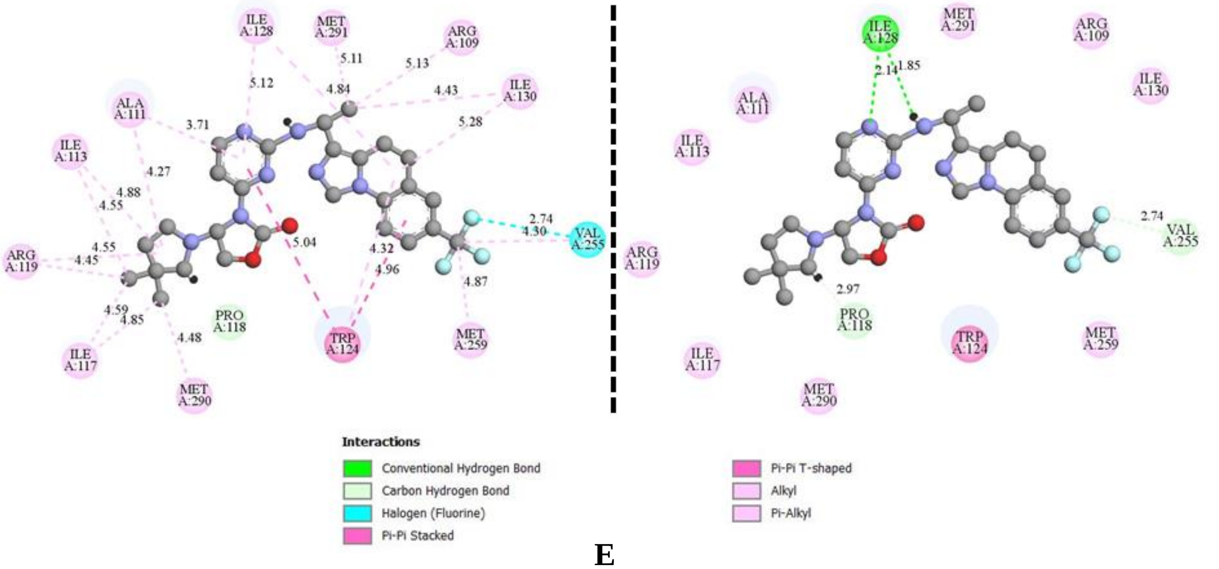

E

Figure S1-E: The molecular docking results for the designed compound (DC-03) interaction with the receptor.

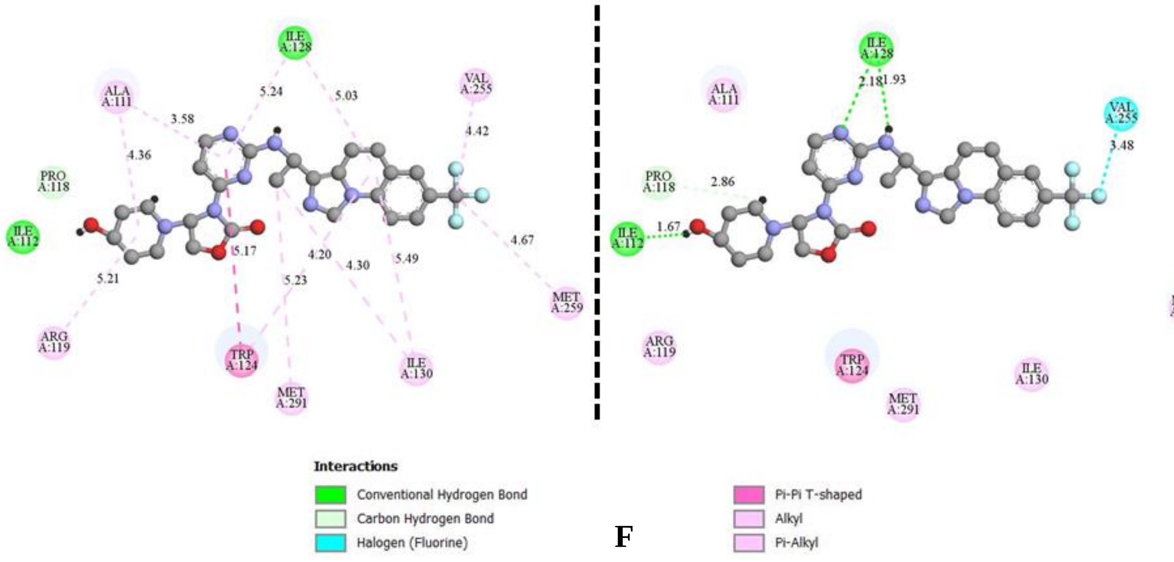

Figure S1-F: The molecular docking results for the designed compound (DC-04) interaction with the receptor.

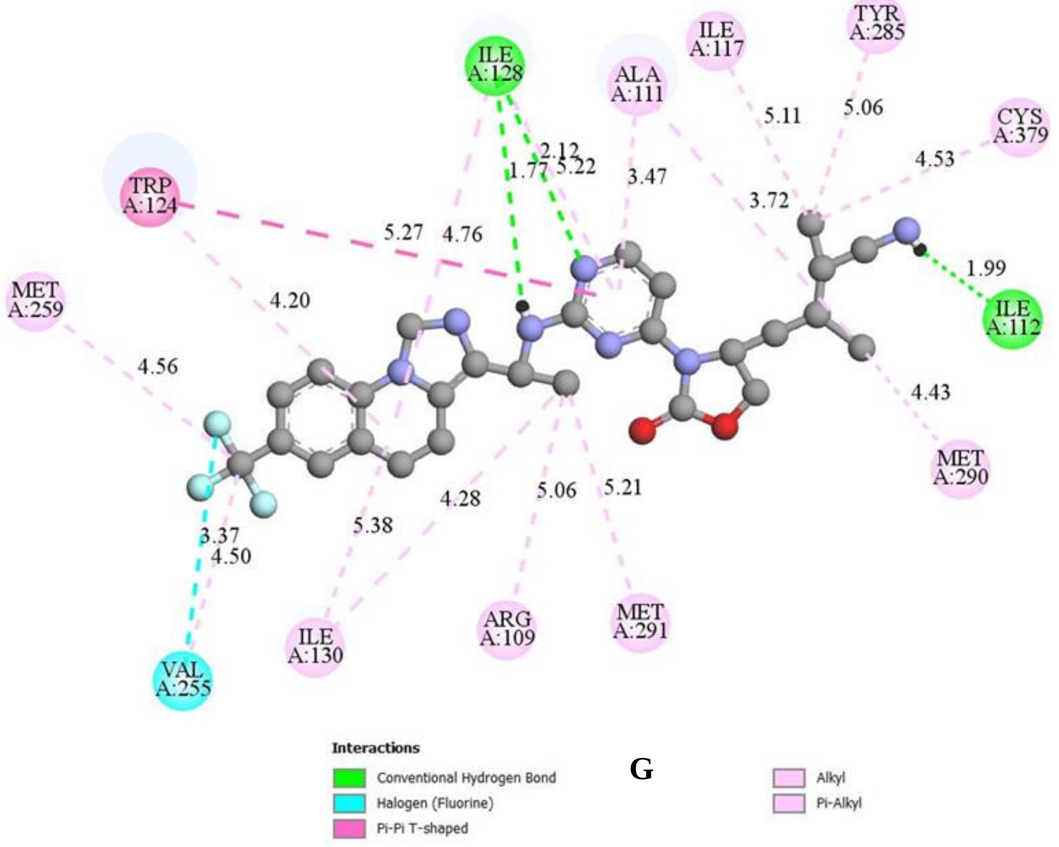

Figure S1-G: The molecular docking results for the designed compound (DC-05) interaction with the receptor.

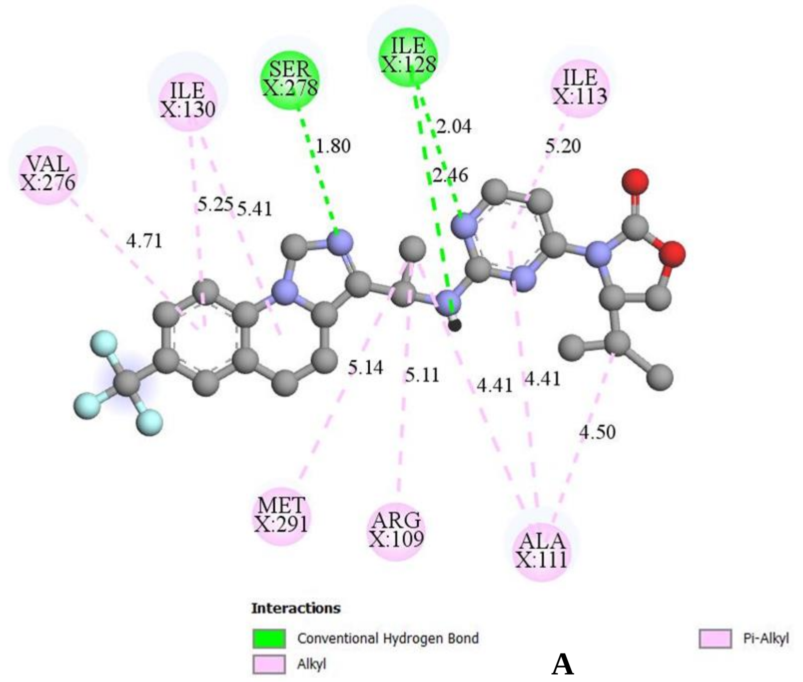

Figure S2-A: The MD simulation results for reference compound (RC-01) interaction with the receptor at the final frame (50 ns).

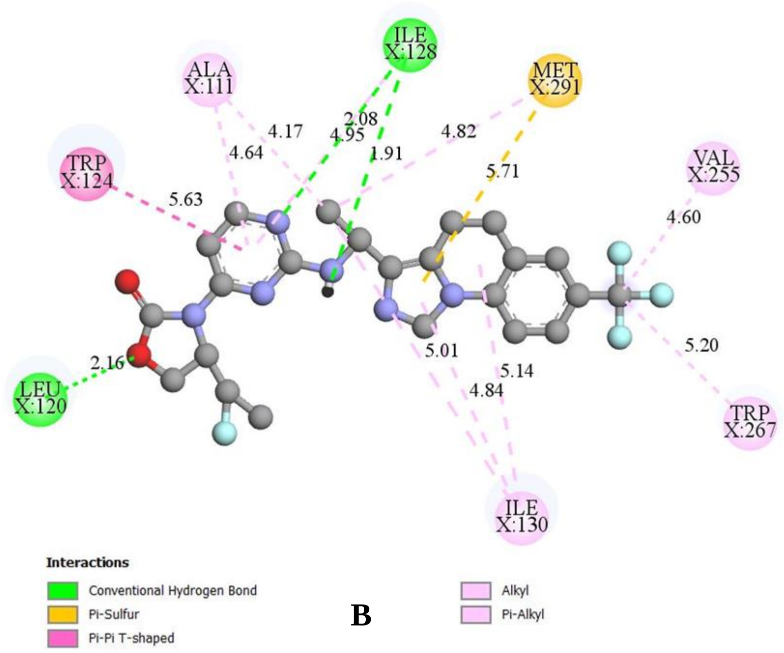

Figure S2 -B: The MD simulation results for reference compound (RC-02) interaction with the receptor at the final frame (50 ns).

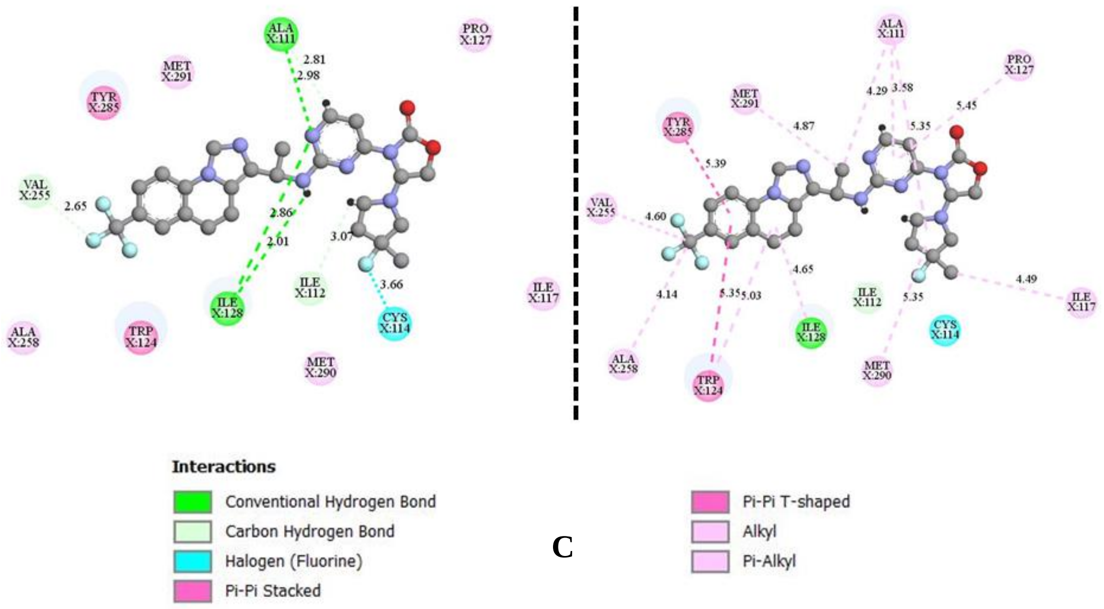

Figure S2-C: The MD simulation results for designed compound (DC-01) interaction with the receptor at the final frame (50 ns).

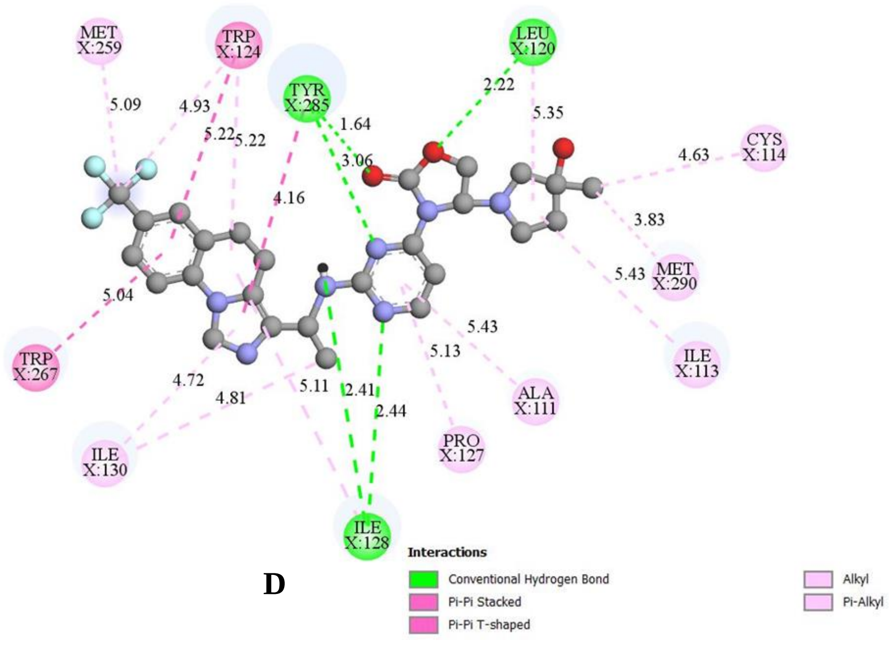

Figure S2-D: The MD simulation results for designed compound (DC-02) interaction with the receptor at the final frame (50 ns).

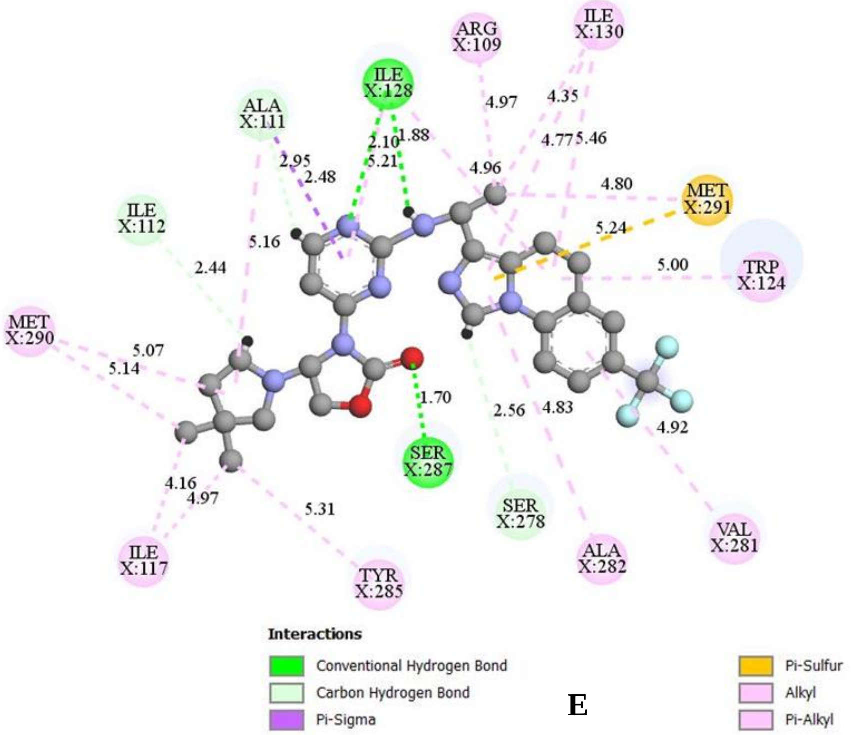

Figure S2-E: The MD simulation results for designed compound (DC-03) interaction with the receptor at the final frame (50 ns).

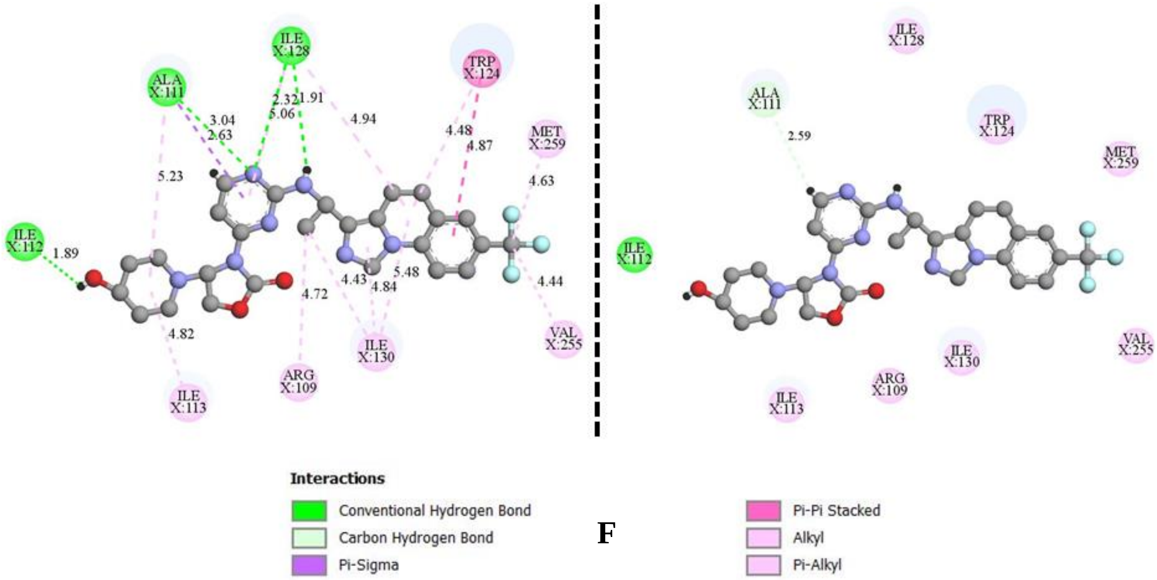

Figure S2-F: The MD simulation results for designed compound (DC-04) interaction with the receptor at the final frame (50 ns).

Table S1: The PLS statistical data of the homogenous 3D-QSAR model using the Field-Based method.

PLS Factors	SD	R^2	F	P	Stability	RMSE	Q^2	Pearson-R
1	0.5453	0.1751	9.1	0.00422	0.893	0.93	0.1641	0.4720
2	0.4141	0.5354	24.2	1.02e-07	0.357	0.72	0.5039	0.7400
3	0.3613	0.6547	25.9	1.46e-09	0.663	0.72	0.4957	0.7150
4	0.3092	0.7533	30.5	1.12e-11	0.652	0.73	0.4794	0.6998
5	0.2534	0.8384	40.5	2.03e-14	0.422	0.73	0.4792	0.6996
6	0.2346	0.8651	40.6	4.73e-15	0.398	0.73	0.4914	0.7096
**7**	**0.1984**	**0.9060**	**51.0**	**4.40e-17**	**0.292**	**0.71**	**0.5123**	**0.7238**

SD: Standard Deviation of regression, R^2: Regression coefficient; F: Ratio of the model variance to the observed activity variance (variance ratio); P: Significance level of variance ratio; RMSE: Root Mean Square Error; Q2: Cross validated correlation coefficient.

Table S2: The physiochemical property contribution of reported compounds using the FB-3D-QSAR method.

PLS Factors	GS	GE	GH	GHA	GHD
1	0.6113	0.0444	0.2500	0.0857	0.0082
2	0.5274	0.0611	0.2861	0.1143	0.0155
3	0.5002	0.0620	0.2861	0.1311	0.0204
4	0.4559	0.0701	0.2945	0.1511	0.0281
5	0.4536	0.0781	0.2966	0.1415	0.0300
6	0.4588	0.0814	0.2873	0.1466	0.0257
**7**	**0.4525**	**0.0936**	**0.2949**	**0.1386**	**0.0201**

Table S3: The Molecular Docking results for 62 reported compounds using Glide package in Schrodinger suite. All the compounds are aligned based on the docking results.

S.NO	Reported compounds	Docking Score kcal/mol	Glide Score kcal/mol	Glide Energy kcal/mol	Biological activity
01	**RC-02**	**-12.40318578**	**-12.49798578**	**-58.60887909**	**8.398**
02	RC-40	-12.21519926	-12.36629926	-56.40876627	7.321
03	RC-37	-12.21033635	-12.30513635	-57.47235823	7.370
04	RC-58	-12.02730268	-12.03210268	-60.42112255	6.215
05	RC-07	-12.0184906	-12.6269906	-63.66662788	8.569
06	RC-41	-11.94412274	-11.94722274	-59.28271866	7.310
07	RC-06	-11.90942478	-12.08072478	-59.04856968	8.638
08	RC-27	-11.84637272	-12.48377272	-62.59935284	7.476
09	RC-18	-11.80124026	-12.20064026	-61.06906796	7.623
10	**RC-01**	**-11.80046427**	**-12.98826427**	**-63.1635437**	**9.000**
11	RC-05	-11.70091038	-12.15221038	-59.23640299	8.658
12	RC-20	-11.65838146	-11.87388146	-56.37018681	7.618
13	RC-39	-11.64871672	-12.16491672	-61.18842983	7.328
14	RC-12	-11.63258244	-12.43048244	-60.32015228	7.824
15	RC-55	-11.62419454	-11.67379454	-58.11397839	6.575
16	RC-19	-11.60019598	-12.11639598	-60.05854416	7.620
17	RC-24	-11.49550681	-12.01170681	-58.28211498	7.523
18	RC-61	-11.4676318	-11.4815318	-55.4534359	6.080
19	RC-15	-11.45380686	-11.56860686	-57.41664314	7.735
20	RC-32	-11.3698159	-11.5505159	-56.02596569	7.409
21	RC-44	-11.36904125	-11.52704125	-55.22951841	7.252

22	RC-10	-11.3640964	-11.9280964	-59.91413975	8.097
23	RC-33	-11.30474686	-11.59564686	-56.48451948	7.398
24	RC-25	-11.28585811	-11.56525811	-54.37228632	7.495
25	RC-13	-11.28509098	-11.81229098	-60.23165703	7.824
26	RC-45	-11.28263235	-11.88163235	-62.34188271	7.208
27	RC-57	-11.27823547	-11.79443547	-59.57751942	6.252
28	RC-08	-11.27605819	-11.79735819	-58.58960819	8.398
29	RC-23	-11.24355768	-11.63265768	-56.14816046	7.569
30	RC-43	-11.1914685	-11.6390685	-57.25476646	7.268
31	RC-21	-11.13683151	-11.48823151	-54.0137372	7.602
32	RC-17	-11.13086443	-11.64706443	-56.51780701	7.658
33	RC-35	-11.06232915	-11.53952915	-54.16304111	7.377
34	RC-59	-11.05034943	-11.39204943	-54.06650257	6.142
35	RC-53	-10.97477142	-11.49097142	-56.54889679	6.620
36	RC-29	-10.96619532	-11.30789532	-53.33992529	7.456
37	RC-11	-10.95076876	-11.46696876	-56.21823978	7.886
38	RC-51	-10.93553125	-11.31873125	-54.81498146	6.818
39	RC-52	-10.93060724	-11.31380724	-55.27937222	6.658
40	RC-46	-10.87551599	-11.10511599	-54.28591919	7.187
41	RC-22	-10.849342	-10.858442	-58.61487389	7.585
42	RC-09	-10.81190734	-10.81190734	-52.78634357	8.301
43	RC-49	-10.79538276	-10.87008276	-54.0499835	6.922
44	RC-48	-10.78823451	-11.17733451	-54.54394579	6.951
45	RC-30	-10.54700017	-11.02420017	-56.28851223	7.444

46	RC-04	-10.02696142	-10.11436142	-54.87780547	8.674
47	RC-34	-9.97525621	-12.19455621	-57.56530237	7.387
48	RC-54	-9.754459832	-11.43715983	-55.98697281	6.602
49	RC-26	-9.057435743	-9.546235743	-48.89270401	7.495
50	RC-28	-8.646313018	-9.823513018	-58.82368296	7.471
51	RC-36	-8.603063268	-10.95946327	-50.44523573	7.377
52	RC-62	-8.586519258	-8.680119258	-52.477736	5.000
53	RC-50	-8.32615672	-8.41205672	-44.42000431	6.891
54	RC-47	-8.23017378	-10.46047378	-58.3135004	7.071
55	RC-16	-8.228324798	-10.8175248	-56.40748596	7.717
56	RC-14	-8.084629328	-8.172029328	-34.85871998	7.810
57	RC-42	-7.811795397	-9.003095397	-43.61901388	7.294
58	RC-03	-7.487671213	-8.664871213	-47.4781667	8.678
59	RC-38	-7.455906451	-10.01620645	-51.48286676	7.354
60	RC-60	-7.241337079	-7.248437079	-50.24009466	6.086
61	RC-56	-7.207464337	-7.533064337	-49.15235996	6.320
62	RC-31	-7.064354773	-7.069154773	-49.39739859	7.444

Table S4A: The Molecular Docking results for 229 designed compounds using Glide package in Schrodinger suite. All the compounds are aligned based on the docking results and functional group modifications at 4th position of oxazolidine moiety especially in hydrophobic and steric nature.

S.NO	Designed Compounds	Core SMARTS	Force Field	Predicted Activity 7	R-Group Modification	Docking Score kcal/mol	Glide Score kcal/mol	Glide Energy kcal/mol
1	DC-01	A=A1AAAA1a2aaaa(a2)AAa(aa3)a(a34)AAa5a4aaaa5	OPLS-2005	9.272	[*]N(C1)CCC1(F)C	-13.337	-13.424	-68.865
2	DC-02	A=A1AAAA1a2aaaa(a2)AAa(aa3)a(a34)AAa5a4aaaa5	OPLS-2005	9.180	[*]N(C1)CCC1(C)O	-13.175	-13.271	-70.037
3	DC-03	A=A1AAAA1a2aaaa(a2)AAa(aa3)a(a34)AAa5a4aaaa5	OPLS-2005	9.675	[*]N(C1)CCC1(C)C	-13.160	-13.256	-70.976
4	DC-04	A=A1AAAA1a2aaaa(a2)AAa(aa3)a(a34)AAa5a4aaaa5	OPLS-2005	9.066	C1CN([*])CCC1O	-13.131	-13.219	-71.142
5	DC-05	A=A1AAAA1a2aaaa(a2)AAa(aa3)a(a34)AAa5a4aaaa5	OPLS-2005	9.154	[*]CC(C)[C@@H](C)CN	-12.952	-13.045	-65.953
6	DC-06	A=A1AAAA1a2aaaa(a2)AAa(aa3)a(a34)AAa5a4aaaa5	OPLS-2005	9.359	[*]N(C1)CC[C@@H]1CC	-12.789	-12.887	-63.641
7	DC-07	A=A1AAAA1a2aaaa(a2)AAa(aa3)a(a34)AAa5a4aaaa5	OPLS-2005	9.132	[*]N(C1)CCC1(C)N	-12.717	-12.804	-69.332
8	DC-08	A=A1AAAA1a2aaaa(a2)AAa(aa3)a(a34)AAa5a4aaaa5	OPLS-2005	9.122	[*]CCC(C)(C)CO	-12.692	-12.786	-66.540
9	DC-09	A=A1AAAA1a2aaaa(a2)AAa(aa3)a(a34)AAa5a4aaaa5	OPLS-2005	9.212	[*]N(C1)CCC1[NH-]	-12.479	-12.631	-70.160
10	DC-10	A=A1AAAA1a2aaaa(a2)AAa(aa3)a(a34)AAa5a4aaaa5	OPLS-2005	9.329	[*]CC1(C)C(C)(C)C1	-12.449	-12.541	-59.467
11	DC-11	A=A1AAAA1a2aaaa(a2)AAa(aa3)a(a34)AAa5a4aaaa5	OPLS-2005	9.066	C1CN([*])CCC1O	-12.407	-13.584	-75.126
12	DC-12	A=A1AAAA1a2aaaa(a2)AAa(aa3)a(a34)AAa5a4aaaa5	OPLS-2005	9.122	[*]CCC(C)(C)CO	-12.348	-13.532	-70.932
13	DC-13	A=A1AAAA1a2aaaa(a2)AAa(aa3)a(a34)AAa5a4aaaa5	OPLS-2005	9.082	[*]C[C@H](F)CCC	-12.296	-12.389	-62.131
14	DC-14	A=A1AAAA1a2aaaa(a2)AAa(aa3)a(a34)AAa5a4aaaa5	OPLS-2005	9.361	[*]N(C1)CC1[C@@H](F)C	-12.250	-12.467	-58.921
15	DC-15	A=A1AAAA1a2aaaa(a2)AAa(aa3)a(a34)AAa5a4aaaa5	OPLS-2005	9.170	[*]OC[C@H](C)C1CC1	-12.229	-12.316	-63.748
16	DC-16	A=A1AAAA1a2aaaa(a2)AAa(aa3)a(a34)AAa5a4aaaa5	OPLS-2005	9.023	[*]N(C1)CC1C#N	-12.126	-12.214	-66.929
17	DC-17	A=A1AAAA1a2aaaa(a2)AAa(aa3)a(a34)AAa5a4aaaa5	OPLS-2005	9.057	[*]C[C@@H](C(C)C)NC	-12.104	-12.191	-64.202
18	DC-18	A=A1AAAA1a2aaaa(a2)AAa(aa3)a(a34)AAa5a4aaaa5	OPLS-2005	9.118	[*]NCC1(F)CCC1	-12.095	-12.197	-62.356

19	DC-19	A=A1AAAA1a2aaaa(a2)AAa(aa3)a(a34)AAa5a4aaaa5	OPLS-2005	9.115	[*]N(C1)CC1(C)CN	-12.094	-12.522	-59.969
20	DC-20	A=A1AAAA1a2aaaa(a2)AAa(aa3)a(a34)AAa5a4aaaa5	OPLS-2005	9.171	O1CCN([*])C[C@@H]1C	-12.054	-12.142	-66.797
21	DC-21	A=A1AAAA1a2aaaa(a2)AAa(aa3)a(a34)AAa5a4aaaa5	OPLS-2005	9.125	[*]N(C)C1(CO)CC1	-11.971	-12.059	-62.701
22	DC-22	A=A1AAAA1a2aaaa(a2)AAa(aa3)a(a34)AAa5a4aaaa5	OPLS-2005	9.261	[*]N(C1)CC1(N)CC	-11.943	-12.630	-62.174
23	DC-23	A=A1AAAA1a2aaaa(a2)AAa(aa3)a(a34)AAa5a4aaaa5	OPLS-2005	9.111	[*]N(C1)CC1CCO	-11.929	-13.114	-68.208
24	DC-24	A=A1AAAA1a2aaaa(a2)AAa(aa3)a(a34)AAa5a4aaaa5	OPLS-2005	9.359	[*]N(C1)CC[C@@H]1CC	-11.850	-13.038	-65.604
25	DC-25	A=A1AAAA1a2aaaa(a2)AAa(aa3)a(a34)AAa5a4aaaa5	OPLS-2005	9.361	[*]N(C1)CC1[C@@H](F)C	-11.850	-12.902	-62.570
26	DC-26	A=A1AAAA1a2aaaa(a2)AAa(aa3)a(a34)AAa5a4aaaa5	OPLS-2005	9.291	[*]CC(C)CCC	-11.800	-11.895	-61.609
27	DC-27	A=A1AAAA1a2aaaa(a2)AAa(aa3)a(a34)AAa5a4aaaa5	OPLS-2005	9.125	[*]N(C)C1(CO)CC1	-11.779	-12.956	-64.924
28	DC-28	A=A1AAAA1a2aaaa(a2)AAa(aa3)a(a34)AAa5a4aaaa5	OPLS-2005	9.118	[*]NCC1(F)CCC1	-11.726	-12.918	-66.453
29	DC-29	A=A1AAAA1a2aaaa(a2)AAa(aa3)a(a34)AAa5a4aaaa5	OPLS-2005	9.033	[*]N(C1)CC1(F)CN	-11.717	-12.940	-68.027
30	DC-30	A=A1AAAA1a2aaaa(a2)AAa(aa3)a(a34)AAa5a4aaaa5	OPLS-2005	9.246	[*]N(C)C(C)(C)CC	-11.715	-11.811	-60.795
31	DC-31	A=A1AAAA1a2aaaa(a2)AAa(aa3)a(a34)AAa5a4aaaa5	OPLS-2005	9.195	[*]N(C)C1(C)CC1	-11.670	-11.757	-60.891
32	DC-32	A=A1AAAA1a2aaaa(a2)AAa(aa3)a(a34)AAa5a4aaaa5	OPLS-2005	9.193	[*]N(C1)CCCC1O	-11.662	-11.759	-58.350
33	DC-33	A=A1AAAA1a2aaaa(a2)AAa(aa3)a(a34)AAa5a4aaaa5	OPLS-2005	9.212	[*]N(C1)CCC1[NH-]	-11.655	-12.896	-72.199
34	DC-34	A=A1AAAA1a2aaaa(a2)AAa(aa3)a(a34)AAa5a4aaaa5	OPLS-2005	9.187	[*]CCC(C)CN	-11.644	-12.826	-66.645
35	DC-35	A=A1AAAA1a2aaaa(a2)AAa(aa3)a(a34)AAa5a4aaaa5	OPLS-2005	9.173	[*]N(C)C(C)CC	-11.625	-11.722	-62.239
36	DC-36	A=A1AAAA1a2aaaa(a2)AAa(aa3)a(a34)AAa5a4aaaa5	OPLS-2005	9.208	[*]N1CCCC1CO	-11.624	-11.712	-61.083
37	DC-37	A=A1AAAA1a2aaaa(a2)AAa(aa3)a(a34)AAa5a4aaaa5	OPLS-2005	9.061	[*]N1CC[C@@H]1CC	-11.622	-11.715	-52.290
38	DC-38	A=A1AAAA1a2aaaa(a2)AAa(aa3)a(a34)AAa5a4aaaa5	OPLS-2005	9.210	[*]N(C1)CC1(F)C	-11.547	-12.588	-59.526
39	DC-39	A=A1AAAA1a2aaaa(a2)AAa(aa3)a(a34)AAa5a4aaaa5	OPLS-2005	9.217	[*]CC(C)(C)CCO	-11.523	-11.617	-61.726
40	DC-40	A=A1AAAA1a2aaaa(a2)AAa(aa3)a(a34)AAa5a4aaaa5	OPLS-2005	9.064	CC(C)N([*])C(C)C	-11.389	-11.483	-58.072
41	DC-41	A=A1AAAA1a2aaaa(a2)AAa(aa3)a(a34)AAa5a4aaaa5	OPLS-2005	9.092	[*]N(C1)CC1C	-11.326	-11.420	-58.496

42	DC-42	A=A1AAAA1a2aaaa(a2)AAa(aa3)a(a34)AAa5a4aaaa5	OPLS-2005	9.195	[*]N(C)C1(C)CC1	-11.305	-12.482	-61.733
43	DC-43	A=A1AAAA1a2aaaa(a2)AAa(aa3)a(a34)AAa5a4aaaa5	OPLS-2005	9.026	[*]N(C1)CC[C@@H]1O	-11.289	-12.466	-70.195
44	DC-44	A=A1AAAA1a2aaaa(a2)AAa(aa3)a(a34)AAa5a4aaaa5	OPLS-2005	9.068	[*]N(C)C1(C)CCC1	-11.278	-11.380	-53.988
45	DC-45	A=A1AAAA1a2aaaa(a2)AAa(aa3)a(a34)AAa5a4aaaa5	OPLS-2005	9.032	[*]N(C)C1(CN)CC1	-11.249	-11.336	-60.047
46	DC-46	A=A1AAAA1a2aaaa(a2)AAa(aa3)a(a34)AAa5a4aaaa5	OPLS-2005	9.035	[*]N1CCC1C	-11.196	-11.289	-57.093
47	DC-47	A=A1AAAA1a2aaaa(a2)AAa(aa3)a(a34)AAa5a4aaaa5	OPLS-2005	9.157	[*]N(C1)CC1(F)C#N	-11.139	-11.227	-64.682
48	DC-48	A=A1AAAA1a2aaaa(a2)AAa(aa3)a(a34)AAa5a4aaaa5	OPLS-2005	9.055	C1CN([*])CCC1N	-11.113	-11.201	-63.100
49	DC-49	A=A1AAAA1a2aaaa(a2)AAa(aa3)a(a34)AAa5a4aaaa5	OPLS-2005	9.121	[*]CC(N)(CC)CC	-11.101	-11.189	-52.628
50	DC-50	A=A1AAAA1a2aaaa(a2)AAa(aa3)a(a34)AAa5a4aaaa5	OPLS-2005	9.167	[*]N(C1)CCC[C@@H]1N	-11.096	-11.184	-60.253
51	DC-51	A=A1AAAA1a2aaaa(a2)AAa(aa3)a(a34)AAa5a4aaaa5	OPLS-2005	9.210	[*]N(C1)CC1(F)C	-11.095	-11.315	-57.689
52	DC-52	A=A1AAAA1a2aaaa(a2)AAa(aa3)a(a34)AAa5a4aaaa5	OPLS-2005	9.212	[*]N(C1)CCC1[NH-]	-11.068	-12.507	-67.600
53	DC-53	A=A1AAAA1a2aaaa(a2)AAa(aa3)a(a34)AAa5a4aaaa5	OPLS-2005	9.487	[*]N(C1)CCC[C@@H]1C	-11.037	-13.116	-64.469
54	DC-54	A=A1AAAA1a2aaaa(a2)AAa(aa3)a(a34)AAa5a4aaaa5	OPLS-2005	9.192	[*]N1CCCC1F	-11.031	-11.118	-61.265
55	DC-55	A=A1AAAA1a2aaaa(a2)AAa(aa3)a(a34)AAa5a4aaaa5	OPLS-2005	9.286	[*]N(C1)CC1(C)OC	-11.028	-11.516	-57.233
56	DC-56	A=A1AAAA1a2aaaa(a2)AAa(aa3)a(a34)AAa5a4aaaa5	OPLS-2005	9.096	[*]N(C1)CC1(C)CO	-10.996	-12.196	-65.871
57	DC-57	A=A1AAAA1a2aaaa(a2)AAa(aa3)a(a34)AAa5a4aaaa5	OPLS-2005	9.407	[*]N(C1)CCC1C	-10.965	-12.151	-56.812
58	DC-58	A=A1AAAA1a2aaaa(a2)AAa(aa3)a(a34)AAa5a4aaaa5	OPLS-2005	9.035	[*]N1CCC1C	-10.941	-12.124	-62.715
59	DC-59	A=A1AAAA1a2aaaa(a2)AAa(aa3)a(a34)AAa5a4aaaa5	OPLS-2005	9.504	[*]CCC1CCCC1	-10.940	-12.124	-60.539
60	DC-60	A=A1AAAA1a2aaaa(a2)AAa(aa3)a(a34)AAa5a4aaaa5	OPLS-2005	9.372	[*]N1CCCC1C	-10.871	-12.058	-55.815
61	DC-61	A=A1AAAA1a2aaaa(a2)AAa(aa3)a(a34)AAa5a4aaaa5	OPLS-2005	9.125	CC(C)N([*])C1CC1	-10.858	-12.035	-48.043
62	DC-62	A=A1AAAA1a2aaaa(a2)AAa(aa3)a(a34)AAa5a4aaaa5	OPLS-2005	9.084	[*]CCC[C@@H](F)C	-10.846	-10.940	-57.378
63	DC-63	A=A1AAAA1a2aaaa(a2)AAa(aa3)a(a34)AAa5a4aaaa5	OPLS-2005	9.121	[*]CC(N)(CC)CC	-10.828	-12.005	-60.943
64	DC-64	A=A1AAAA1a2aaaa(a2)AAa(aa3)a(a34)AAa5a4aaaa5	OPLS-2005	9.211	[*]N1CCCC1C#N	-10.806	-11.983	-55.923

65	DC-65	A=A1AAAA1a2aaaa(a2)AAa(aa3)a(a34)AAa5a4aaaa5	OPLS-2005	9.046	[*]c1c(C)cccc1	-10.795	-10.882	-57.258
66	DC-66	A=A1AAAA1a2aaaa(a2)AAa(aa3)a(a34)AAa5a4aaaa5	OPLS-2005	9.261	[*]N(C1)CC1(N)CC	-10.735	-12.512	-65.177
67	DC-67	A=A1AAAA1a2aaaa(a2)AAa(aa3)a(a34)AAa5a4aaaa5	OPLS-2005	9.063	[*]c1ccc(C)n1C	-10.644	-10.731	-54.239
68	DC-68	A=A1AAAA1a2aaaa(a2)AAa(aa3)a(a34)AAa5a4aaaa5	OPLS-2005	9.009	[*]N1CCN(C)CC1	-10.641	-11.936	-62.996
69	DC-69	A=A1AAAA1a2aaaa(a2)AAa(aa3)a(a34)AAa5a4aaaa5	OPLS-2005	9.044	[*]CC(C)C[C@H](C)O	-10.567	-10.661	-54.325
70	DC-70	A=A1AAAA1a2aaaa(a2)AAa(aa3)a(a34)AAa5a4aaaa5	OPLS-2005	9.132	[*]N(C1)CCC1(C)N	-10.524	-11.701	-63.896
71	DC-71	A=A1AAAA1a2aaaa(a2)AAa(aa3)a(a34)AAa5a4aaaa5	OPLS-2005	9.170	[*]N(C1)CC[C@@H]1CO	-10.470	-11.652	-64.204
72	DC-72	A=A1AAAA1a2aaaa(a2)AAa(aa3)a(a34)AAa5a4aaaa5	OPLS-2005	9.180	[*]N(C1)CCC1(C)O	-10.463	-12.978	-67.820
73	DC-73	A=A1AAAA1a2aaaa(a2)AAa(aa3)a(a34)AAa5a4aaaa5	OPLS-2005	9.057	[*]N(C1)CC1[C@H](C)O	-10.443	-10.539	-58.524
74	DC-74	A=A1AAAA1a2aaaa(a2)AAa(aa3)a(a34)AAa5a4aaaa5	OPLS-2005	9.096	[*]N(C1)CC1(C)CO	-10.423	-12.379	-64.764
75	DC-75	A=A1AAAA1a2aaaa(a2)AAa(aa3)a(a34)AAa5a4aaaa5	OPLS-2005	9.193	[*]N(C1)CCCC1O	-10.356	-12.789	-65.438
76	DC-76	A=A1AAAA1a2aaaa(a2)AAa(aa3)a(a34)AAa5a4aaaa5	OPLS-2005	9.143	[*]N(C1)CC1(F)F	-10.279	-11.456	-55.694
77	DC-77	A=A1AAAA1a2aaaa(a2)AAa(aa3)a(a34)AAa5a4aaaa5	OPLS-2005	9.407	[*]N(C1)CCC1C	-10.089	-12.566	-65.029
78	DC-78	A=A1AAAA1a2aaaa(a2)AAa(aa3)a(a34)AAa5a4aaaa5	OPLS-2005	9.033	[*]N(C1)CC1(F)CN	-9.950	-11.573	-57.012
79	DC-79	A=A1AAAA1a2aaaa(a2)AAa(aa3)a(a34)AAa5a4aaaa5	OPLS-2005	9.261	[*]N(C1)CC1(N)CC	-9.783	-12.251	-59.586
80	DC-80	A=A1AAAA1a2aaaa(a2)AAa(aa3)a(a34)AAa5a4aaaa5	OPLS-2005	9.211	[*]N1CCCC1C#N	-9.627	-9.714	-39.561
81	DC-81	A=A1AAAA1a2aaaa(a2)AAa(aa3)a(a34)AAa5a4aaaa5	OPLS-2005	9.228	CCN([*])C(C)(C)C	-9.401	-12.055	-62.506
82	DC-82	A=A1AAAA1a2aaaa(a2)AAa(aa3)a(a34)AAa5a4aaaa5	OPLS-2005	9.279	[*]N(C1)CC1CCN	-9.396	-11.174	-59.200
83	DC-83	A=A1AAAA1a2aaaa(a2)AAa(aa3)a(a34)AAa5a4aaaa5	OPLS-2005	9.115	[*]N(C1)CC1(C)CN	-9.334	-11.003	-58.611
84	DC-84	A=A1AAAA1a2aaaa(a2)AAa(aa3)a(a34)AAa5a4aaaa5	OPLS-2005	9.170	[*]N(C1)CC[C@@H]1CN	-9.325	-9.412	-53.968
85	DC-85	A=A1AAAA1a2aaaa(a2)AAa(aa3)a(a34)AAa5a4aaaa5	OPLS-2005	9.261	[*]N(C1)CC1(N)CC	-9.322	-10.782	-58.587
86	DC-86	A=A1AAAA1a2aaaa(a2)AAa(aa3)a(a34)AAa5a4aaaa5	OPLS-2005	9.407	[*]N(C1)CC1(F)CC	-9.295	-10.209	-39.302
87	DC-87	A=A1AAAA1a2aaaa(a2)AAa(aa3)a(a34)AAa5a4aaaa5	OPLS-2005	9.261	[*]N(C1)CC[C@@H]1C#N	-9.289	-9.376	-39.147

88	DC-88	A=A1AAAA1a2aaaa(a2)AAa(aa3)a(a34)AAa5a4aaaa5	OPLS-2005	9.175	[*]N1CCC[C@@H]1CN	-9.271	-9.358	-57.009
89	DC-89	A=A1AAAA1a2aaaa(a2)AAa(aa3)a(a34)AAa5a4aaaa5	OPLS-2005	9.223	[*]N(C1)CC1(O)CC	-9.249	-9.336	-51.888
90	DC-90	A=A1AAAA1a2aaaa(a2)AAa(aa3)a(a34)AAa5a4aaaa5	OPLS-2005	9.487	[*]N(C1)CCC[C@@H]1C	-9.205	-9.311	-55.005
91	DC-91	A=A1AAAA1a2aaaa(a2)AAa(aa3)a(a34)AAa5a4aaaa5	OPLS-2005	9.033	[*]N(C1)CC1(F)CN	-9.205	-11.917	-61.222
92	DC-92	A=A1AAAA1a2aaaa(a2)AAa(aa3)a(a34)AAa5a4aaaa5	OPLS-2005	9.292	[*]N1CCCCC1F	-9.196	-9.284	-50.409
93	DC-93	A=A1AAAA1a2aaaa(a2)AAa(aa3)a(a34)AAa5a4aaaa5	OPLS-2005	9.372	[*]N1CCCC1C	-9.124	-11.600	-54.342
94	DC-94	A=A1AAAA1a2aaaa(a2)AAa(aa3)a(a34)AAa5a4aaaa5	OPLS-2005	9.173	[*]N(C)C(C)CC	-9.107	-11.575	-61.354
95	DC-95	A=A1AAAA1a2aaaa(a2)AAa(aa3)a(a34)AAa5a4aaaa5	OPLS-2005	9.212	[*]N(C1)CCC1[NH-]	-9.043	-11.572	-55.816
96	DC-96	A=A1AAAA1a2aaaa(a2)AAa(aa3)a(a34)AAa5a4aaaa5	OPLS-2005	9.154	[*]CC(C)[C@@H](C)CN	-9.032	-10.215	-57.438
97	DC-97	A=A1AAAA1a2aaaa(a2)AAa(aa3)a(a34)AAa5a4aaaa5	OPLS-2005	9.138	[*]CCC1(F)CNC1	-8.967	-10.161	-53.878
98	DC-98	A=A1AAAA1a2aaaa(a2)AAa(aa3)a(a34)AAa5a4aaaa5	OPLS-2005	9.286	[*]N(C1)CC1(C)OC	-8.847	-10.445	-59.044
99	DC-99	A=A1AAAA1a2aaaa(a2)AAa(aa3)a(a34)AAa5a4aaaa5	OPLS-2005	9.046	[*]c1c(C)cccc1	-8.839	-10.017	-55.731
100	DC-100	A=A1AAAA1a2aaaa(a2)AAa(aa3)a(a34)AAa5a4aaaa5	OPLS-2005	9.175	[*]N1CCC[C@@H]1CN	-8.807	-9.985	-51.940
101	DC-101	A=A1AAAA1a2aaaa(a2)AAa(aa3)a(a34)AAa5a4aaaa5	OPLS-2005	9.513	[*]N(C1)CC1C(C)C	-8.799	-8.886	-51.634
102	DC-102	A=A1AAAA1a2aaaa(a2)AAa(aa3)a(a34)AAa5a4aaaa5	OPLS-2005	9.261	[*]N(C1)CC1(N)CC	-8.724	-9.094	-50.694
103	DC-103	A=A1AAAA1a2aaaa(a2)AAa(aa3)a(a34)AAa5a4aaaa5	OPLS-2005	9.092	[*]N(C1)CC1C	-8.600	-11.323	-47.846
104	DC-104	A=A1AAAA1a2aaaa(a2)AAa(aa3)a(a34)AAa5a4aaaa5	OPLS-2005	9.167	[*]N(C1)CCC[C@@H]1N	-8.520	-9.697	-57.696
105	DC-105	A=A1AAAA1a2aaaa(a2)AAa(aa3)a(a34)AAa5a4aaaa5	OPLS-2005	9.675	[*]N(C1)CCC1(C)C	-8.489	-9.675	-58.878
106	DC-106	A=A1AAAA1a2aaaa(a2)AAa(aa3)a(a34)AAa5a4aaaa5	OPLS-2005	9.170	[*]N(C1)CC[C@@H]1CO	-8.485	-8.578	-47.879
107	DC-107	A=A1AAAA1a2aaaa(a2)AAa(aa3)a(a34)AAa5a4aaaa5	OPLS-2005	9.059	[*]CCCC1CNC1	-8.472	-8.560	-48.890
108	DC-108	A=A1AAAA1a2aaaa(a2)AAa(aa3)a(a34)AAa5a4aaaa5	OPLS-2005	9.111	[*]N(C1)CC1CCO	-8.437	-8.533	-44.661
109	DC-109	A=A1AAAA1a2aaaa(a2)AAa(aa3)a(a34)AAa5a4aaaa5	OPLS-2005	9.187	[*]CCC(C)CN	-8.339	-8.432	-52.696
110	DC-110	A=A1AAAA1a2aaaa(a2)AAa(aa3)a(a34)AAa5a4aaaa5	OPLS-2005	9.675	[*]N(C1)CCC1(C)C	-8.282	-10.758	-50.126

111	DC-111	A=A1AAAA1a2aaaa(a2)AAa(aa3)a(a34)AAa5a4aaaa5	OPLS-2005	9.279	[*]N(C1)CC1CCN	-8.188	-8.312	-50.161
112	DC-112	A=A1AAAA1a2aaaa(a2)AAa(aa3)a(a34)AAa5a4aaaa5	OPLS-2005	9.237	[*]N1CC[C@@H]1CCC	-8.181	-8.274	-39.164
113	DC-113	A=A1AAAA1a2aaaa(a2)AAa(aa3)a(a34)AAa5a4aaaa5	OPLS-2005	9.044	[*]CC(C)C[C@H](C)O	-8.147	-10.792	-59.728
114	DC-114	A=A1AAAA1a2aaaa(a2)AAa(aa3)a(a34)AAa5a4aaaa5	OPLS-2005	9.618	[*]N(C1)CC1(C)CC	-8.111	-8.203	-33.524
115	DC-115	A=A1AAAA1a2aaaa(a2)AAa(aa3)a(a34)AAa5a4aaaa5	OPLS-2005	9.115	[*]N(C1)CC1(C)CN	-8.090	-8.668	-47.445
116	DC-116	A=A1AAAA1a2aaaa(a2)AAa(aa3)a(a34)AAa5a4aaaa5	OPLS-2005	9.017	[*]CCCC(C)(C)N	-8.078	-9.255	-57.408
117	DC-117	A=A1AAAA1a2aaaa(a2)AAa(aa3)a(a34)AAa5a4aaaa5	OPLS-2005	9.268	C1CN([*])CCC1F	-8.069	-9.246	-57.391
118	DC-118	A=A1AAAA1a2aaaa(a2)AAa(aa3)a(a34)AAa5a4aaaa5	OPLS-2005	9.023	[*]N(C1)CC1C#N	-8.065	-9.242	-57.060
119	DC-119	A=A1AAAA1a2aaaa(a2)AAa(aa3)a(a34)AAa5a4aaaa5	OPLS-2005	9.345	[*]N(C1)CC1(C)C	-8.058	-9.241	-53.814
120	DC-120	A=A1AAAA1a2aaaa(a2)AAa(aa3)a(a34)AAa5a4aaaa5	OPLS-2005	9.017	[*]CCCC(C)(C)N	-8.032	-8.119	-48.057
121	DC-121	A=A1AAAA1a2aaaa(a2)AAa(aa3)a(a34)AAa5a4aaaa5	OPLS-2005	9.504	[*]CCC1CCCC1	-8.026	-10.697	-45.068
122	DC-122	A=A1AAAA1a2aaaa(a2)AAa(aa3)a(a34)AAa5a4aaaa5	OPLS-2005	9.025	[*]CC(C)(O)CCC	-8.019	-10.536	-58.038
123	DC-123	A=A1AAAA1a2aaaa(a2)AAa(aa3)a(a34)AAa5a4aaaa5	OPLS-2005	9.044	[*]CC(C)C[C@H](C)O	-7.960	-9.144	-55.218
124	DC-124	A=A1AAAA1a2aaaa(a2)AAa(aa3)a(a34)AAa5a4aaaa5	OPLS-2005	9.040	[*]CC(O)(CC)CC	-7.881	-10.394	-56.802
125	DC-125	A=A1AAAA1a2aaaa(a2)AAa(aa3)a(a34)AAa5a4aaaa5	OPLS-2005	9.177	[*]CC[C@@H]1C[C@@H](C1)N	-7.857	-10.536	-58.245
126	DC-126	A=A1AAAA1a2aaaa(a2)AAa(aa3)a(a34)AAa5a4aaaa5	OPLS-2005	9.279	[*]N(C1)CC1CCN	-7.821	-9.034	-53.723
127	DC-127	A=A1AAAA1a2aaaa(a2)AAa(aa3)a(a34)AAa5a4aaaa5	OPLS-2005	9.143	[*]N(C1)CC1(F)F	-7.789	-7.876	-50.294
128	DC-128	A=A1AAAA1a2aaaa(a2)AAa(aa3)a(a34)AAa5a4aaaa5	OPLS-2005	9.032	[*]N(C)C1(CN)CC1	-7.786	-8.963	-54.453
129	DC-129	A=A1AAAA1a2aaaa(a2)AAa(aa3)a(a34)AAa5a4aaaa5	OPLS-2005	9.261	[*]N(C1)CC[C@@H]1C#N	-7.780	-8.957	-54.981
130	DC-130	A=A1AAAA1a2aaaa(a2)AAa(aa3)a(a34)AAa5a4aaaa5	OPLS-2005	9.084	[*]CCC[C@@H](F)C	-7.771	-10.456	-58.151
131	DC-131	A=A1AAAA1a2aaaa(a2)AAa(aa3)a(a34)AAa5a4aaaa5	OPLS-2005	9.061	[*]N1CC[C@@H]1CC	-7.767	-8.950	-44.009
132	DC-132	A=A1AAAA1a2aaaa(a2)AAa(aa3)a(a34)AAa5a4aaaa5	OPLS-2005	9.407	[*]N(C1)CC1(F)CC	-7.739	-7.995	-49.324
133	DC-133	A=A1AAAA1a2aaaa(a2)AAa(aa3)a(a34)AAa5a4aaaa5	OPLS-2005	9.040	[*]CC(O)(CC)CC	-7.732	-8.918	-52.632

134	DC-134	A=A1AAAA1a2aaaa(a2)AAa(aa3)a(a34)AAa5a4aaaa5	OPLS-2005	9.217	[*]CC(C)(C)CCO	-7.701	-8.886	-52.926
135	DC-135	A=A1AAAA1a2aaaa(a2)AAa(aa3)a(a34)AAa5a4aaaa5	OPLS-2005	9.180	[*]N(C1)CCC1(C)O	-7.668	-8.854	-52.881
136	DC-136	A=A1AAAA1a2aaaa(a2)AAa(aa3)a(a34)AAa5a4aaaa5	OPLS-2005	9.064	CC(C)N([*])C(C)C	-7.609	-8.793	-58.191
137	DC-137	A=A1AAAA1a2aaaa(a2)AAa(aa3)a(a34)AAa5a4aaaa5	OPLS-2005	9.021	[*]CC1(C)[C@H](O)CC1	-7.585	-8.769	-54.037
138	DC-138	A=A1AAAA1a2aaaa(a2)AAa(aa3)a(a34)AAa5a4aaaa5	OPLS-2005	9.192	[*]N1CCCC1F	-7.580	-8.757	-51.936
139	DC-139	A=A1AAAA1a2aaaa(a2)AAa(aa3)a(a34)AAa5a4aaaa5	OPLS-2005	9.504	[*]CCC1CCCC1	-7.562	-7.656	-45.210
140	DC-140	A=A1AAAA1a2aaaa(a2)AAa(aa3)a(a34)AAa5a4aaaa5	OPLS-2005	9.422	[*]N(C1)CCC[C@@H]1F	-7.561	-7.648	-49.841
141	DC-141	A=A1AAAA1a2aaaa(a2)AAa(aa3)a(a34)AAa5a4aaaa5	OPLS-2005	9.268	C1CN([*])CCC1F	-7.546	-7.633	-48.616
142	DC-142	A=A1AAAA1a2aaaa(a2)AAa(aa3)a(a34)AAa5a4aaaa5	OPLS-2005	9.057	[*]C[C@@H](C(C)C)NC	-7.529	-8.706	-51.995
143	DC-143	A=A1AAAA1a2aaaa(a2)AAa(aa3)a(a34)AAa5a4aaaa5	OPLS-2005	9.094	[*]CCC1(O)CCC1	-7.507	-7.600	-46.000
144	DC-144	A=A1AAAA1a2aaaa(a2)AAa(aa3)a(a34)AAa5a4aaaa5	OPLS-2005	9.115	[*]N(C1)CC1(C)CN	-7.467	-8.984	-52.033
145	DC-145	A=A1AAAA1a2aaaa(a2)AAa(aa3)a(a34)AAa5a4aaaa5	OPLS-2005	9.025	[*]CC(C)(O)CCC	-7.464	-7.560	-44.591
146	DC-146	A=A1AAAA1a2aaaa(a2)AAa(aa3)a(a34)AAa5a4aaaa5	OPLS-2005	9.389	[*]N(C1)CC1CCC	-7.440	-7.528	-44.831
147	DC-147	A=A1AAAA1a2aaaa(a2)AAa(aa3)a(a34)AAa5a4aaaa5	OPLS-2005	9.358	[*]N1CCCC1(C)C	-7.428	-8.614	-57.076
148	DC-148	A=A1AAAA1a2aaaa(a2)AAa(aa3)a(a34)AAa5a4aaaa5	OPLS-2005	9.389	[*]N(C1)CC1CCC	-7.427	-8.604	-47.589
149	DC-149	A=A1AAAA1a2aaaa(a2)AAa(aa3)a(a34)AAa5a4aaaa5	OPLS-2005	9.358	[*]N1CCCC1(C)C	-7.403	-7.499	-57.615
150	DC-150	A=A1AAAA1a2aaaa(a2)AAa(aa3)a(a34)AAa5a4aaaa5	OPLS-2005	9.026	[*]N(C1)CC[C@@H]1O	-7.401	-7.488	-47.172
151	DC-151	A=A1AAAA1a2aaaa(a2)AAa(aa3)a(a34)AAa5a4aaaa5	OPLS-2005	9.123	[*]CCC(F)(F)F	-7.389	-7.477	-43.366
152	DC-152	A=A1AAAA1a2aaaa(a2)AAa(aa3)a(a34)AAa5a4aaaa5	OPLS-2005	9.515	[*]N1CCCC1CC	-7.373	-7.473	-50.217
153	DC-153	A=A1AAAA1a2aaaa(a2)AAa(aa3)a(a34)AAa5a4aaaa5	OPLS-2005	9.361	[*]N(C1)CC1[C@@H](F)C	-7.334	-8.641	-50.685
154	DC-154	A=A1AAAA1a2aaaa(a2)AAa(aa3)a(a34)AAa5a4aaaa5	OPLS-2005	9.228	CCN([*])C(C)(C)C	-7.321	-7.415	-36.097
155	DC-155	A=A1AAAA1a2aaaa(a2)AAa(aa3)a(a34)AAa5a4aaaa5	OPLS-2005	9.170	[*]N(C1)CC[C@@H]1CN	-7.277	-8.455	-56.161
156	DC-156	A=A1AAAA1a2aaaa(a2)AAa(aa3)a(a34)AAa5a4aaaa5	OPLS-2005	9.255	[*]N(C1)CC1N(C)C	-7.268	-8.579	-52.390

157	DC-157	A=A1AAAA1a2aaaa(a2)AAa(aa3)a(a34)AAa5a4aaaa5	OPLS-2005	9.033	[*]N(C1)CC1(F)CN	-7.194	-7.327	-51.380
158	DC-158	A=A1AAAA1a2aaaa(a2)AAa(aa3)a(a34)AAa5a4aaaa5	OPLS-2005	9.177	[*]CC[C@@H]1C[C@@H](C1)N	-7.175	-7.269	-40.844
159	DC-159	A=A1AAAA1a2aaaa(a2)AAa(aa3)a(a34)AAa5a4aaaa5	OPLS-2005	9.057	[*]N(C1)CC1[C@H](C)O	-7.129	-8.314	-53.166
160	DC-160	A=A1AAAA1a2aaaa(a2)AAa(aa3)a(a34)AAa5a4aaaa5	OPLS-2005	9.021	[*]CC1(C)[C@H](O)CC1	-7.118	-7.212	-51.353
161	DC-161	A=A1AAAA1a2aaaa(a2)AAa(aa3)a(a34)AAa5a4aaaa5	OPLS-2005	9.210	[*]N(C1)CC1(F)C	-7.115	-8.424	-45.724
162	DC-162	A=A1AAAA1a2aaaa(a2)AAa(aa3)a(a34)AAa5a4aaaa5	OPLS-2005	9.246	[*]N(C)C(C)(C)CC	-7.113	-8.300	-52.015
163	DC-163	A=A1AAAA1a2aaaa(a2)AAa(aa3)a(a34)AAa5a4aaaa5	OPLS-2005	9.137	[*]N(C1)CCC1F	-7.107	-7.195	-46.074
164	DC-164	A=A1AAAA1a2aaaa(a2)AAa(aa3)a(a34)AAa5a4aaaa5	OPLS-2005	9.618	[*]N(C1)CC1(C)CC	-7.085	-8.267	-48.916
165	DC-165	A=A1AAAA1a2aaaa(a2)AAa(aa3)a(a34)AAa5a4aaaa5	OPLS-2005	9.170	[*]OC[C@H](C)C1CC1	-7.075	-8.252	-49.673
166	DC-166	A=A1AAAA1a2aaaa(a2)AAa(aa3)a(a34)AAa5a4aaaa5	OPLS-2005	9.323	[*]N(C1)[C@@H](C)C1(C)C	-7.071	-7.164	-43.002
167	DC-167	A=A1AAAA1a2aaaa(a2)AAa(aa3)a(a34)AAa5a4aaaa5	OPLS-2005	9.235	[*]N(C1)CC1CC	-7.053	-7.145	-42.746
168	DC-168	A=A1AAAA1a2aaaa(a2)AAa(aa3)a(a34)AAa5a4aaaa5	OPLS-2005	9.035	[*]N1CCC1C	-7.053	-9.776	-39.104
169	DC-169	A=A1AAAA1a2aaaa(a2)AAa(aa3)a(a34)AAa5a4aaaa5	OPLS-2005	9.345	[*]N(C1)CC1(C)C	-7.015	-7.108	-43.479
170	DC-170	A=A1AAAA1a2aaaa(a2)AAa(aa3)a(a34)AAa5a4aaaa5	OPLS-2005	9.223	[*]N(C1)CC1(O)CC	-6.995	-8.172	-57.095
171	DC-171	A=A1AAAA1a2aaaa(a2)AAa(aa3)a(a34)AAa5a4aaaa5	OPLS-2005	9.487	[*]N(C1)CCC[C@@H]1C	-6.920	-8.116	-49.340
172	DC-172	A=A1AAAA1a2aaaa(a2)AAa(aa3)a(a34)AAa5a4aaaa5	OPLS-2005	9.228	CCN([*])C(C)(C)C	-6.907	-8.091	-46.113
173	DC-173	A=A1AAAA1a2aaaa(a2)AAa(aa3)a(a34)AAa5a4aaaa5	OPLS-2005	9.407	[*]N(C1)CCC1C	-6.864	-6.961	-45.584
174	DC-174	A=A1AAAA1a2aaaa(a2)AAa(aa3)a(a34)AAa5a4aaaa5	OPLS-2005	9.292	[*]N1CCCCC1F	-6.850	-8.027	-52.521
175	DC-175	A=A1AAAA1a2aaaa(a2)AAa(aa3)a(a34)AAa5a4aaaa5	OPLS-2005	9.009	[*]N1CCN(C)CC1	-6.850	-7.055	-44.456
176	DC-176	A=A1AAAA1a2aaaa(a2)AAa(aa3)a(a34)AAa5a4aaaa5	OPLS-2005	9.059	[*]CCCC1CNC1	-6.843	-8.021	-54.697
177	DC-177	A=A1AAAA1a2aaaa(a2)AAa(aa3)a(a34)AAa5a4aaaa5	OPLS-2005	9.323	[*]N(C1)[C@@H](C)C1(C)C	-6.840	-8.023	-46.549
178	DC-178	A=A1AAAA1a2aaaa(a2)AAa(aa3)a(a34)AAa5a4aaaa5	OPLS-2005	9.025	[*]CC(C)(O)CCC	-6.837	-8.023	-57.744
179	DC-179	A=A1AAAA1a2aaaa(a2)AAa(aa3)a(a34)AAa5a4aaaa5	OPLS-2005	9.237	[*]N1CC[C@@H]1CCC	-6.800	-7.983	-46.228

180	DC-180	A=A1AAAA1a2aaaa(a2)AAa(aa3)a(a34)AAa5a4aaaa5	OPLS-2005	9.137	[*]C[C@H](O)CC(C)C	-6.800	-7.983	-53.704
181	DC-181	A=A1AAAA1a2aaaa(a2)AAa(aa3)a(a34)AAa5a4aaaa5	OPLS-2005	9.175	[*]CC[C@H]1CCNC1	-6.799	-6.891	-47.316
182	DC-182	A=A1AAAA1a2aaaa(a2)AAa(aa3)a(a34)AAa5a4aaaa5	OPLS-2005	9.286	[*]N(C1)CC1(C)OC	-6.724	-7.233	-49.074
183	DC-183	A=A1AAAA1a2aaaa(a2)AAa(aa3)a(a34)AAa5a4aaaa5	OPLS-2005	9.092	[*]N(C1)CC1C	-6.709	-7.892	-43.785
184	DC-184	A=A1AAAA1a2aaaa(a2)AAa(aa3)a(a34)AAa5a4aaaa5	OPLS-2005	9.328	[*]N(C1)CC1(C#N)C	-6.684	-6.772	-47.497
185	DC-185	A=A1AAAA1a2aaaa(a2)AAa(aa3)a(a34)AAa5a4aaaa5	OPLS-2005	9.329	[*]CC1(C)C(C)(C)C1	-6.641	-7.823	-55.008
186	DC-186	A=A1AAAA1a2aaaa(a2)AAa(aa3)a(a34)AAa5a4aaaa5	OPLS-2005	9.359	[*]N(C1)CC[C@@H]1CC	-6.625	-9.030	-52.242
187	DC-187	A=A1AAAA1a2aaaa(a2)AAa(aa3)a(a34)AAa5a4aaaa5	OPLS-2005	9.407	[*]N(C1)CC1(F)CC	-6.557	-8.561	-47.077
188	DC-188	A=A1AAAA1a2aaaa(a2)AAa(aa3)a(a34)AAa5a4aaaa5	OPLS-2005	9.192	[*]CCCC(C)C	-6.546	-7.723	-50.618
189	DC-189	A=A1AAAA1a2aaaa(a2)AAa(aa3)a(a34)AAa5a4aaaa5	OPLS-2005	9.343	CCN([*])C1(C)CC1	-6.515	-6.602	-45.372
190	DC-190	A=A1AAAA1a2aaaa(a2)AAa(aa3)a(a34)AAa5a4aaaa5	OPLS-2005	9.515	[*]N1CCCC1CC	-6.476	-7.666	-54.552
191	DC-191	A=A1AAAA1a2aaaa(a2)AAa(aa3)a(a34)AAa5a4aaaa5	OPLS-2005	9.192	[*]CCCC(C)C	-6.464	-6.551	-44.360
192	DC-192	A=A1AAAA1a2aaaa(a2)AAa(aa3)a(a34)AAa5a4aaaa5	OPLS-2005	9.055	C1CN([*])CCC1N	-6.457	-7.634	-53.068
193	DC-193	A=A1AAAA1a2aaaa(a2)AAa(aa3)a(a34)AAa5a4aaaa5	OPLS-2005	9.111	[*]N(C1)CC1CCO	-6.453	-8.974	-50.114
194	DC-194	A=A1AAAA1a2aaaa(a2)AAa(aa3)a(a34)AAa5a4aaaa5	OPLS-2005	9.361	[*]N(C1)CC1[C@@H](F)C	-6.425	-8.567	-50.055
195	DC-195	A=A1AAAA1a2aaaa(a2)AAa(aa3)a(a34)AAa5a4aaaa5	OPLS-2005	9.255	[*]N(C1)CC1N(C)C	-6.378	-7.471	-37.006
196	DC-196	A=A1AAAA1a2aaaa(a2)AAa(aa3)a(a34)AAa5a4aaaa5	OPLS-2005	9.255	[*]N(C1)CC1N(C)C	-6.377	-8.707	-49.751
197	DC-197	A=A1AAAA1a2aaaa(a2)AAa(aa3)a(a34)AAa5a4aaaa5	OPLS-2005	9.235	[*]N(C1)CC1CC	-6.374	-7.556	-38.016
198	DC-198	A=A1AAAA1a2aaaa(a2)AAa(aa3)a(a34)AAa5a4aaaa5	OPLS-2005	9.323	[*]N(C1)[C@@H](C)C1(C)C	-6.369	-9.091	-53.860
199	DC-199	A=A1AAAA1a2aaaa(a2)AAa(aa3)a(a34)AAa5a4aaaa5	OPLS-2005	9.208	[*]N1CCCC1CO	-6.352	-7.529	-53.420
200	DC-200	A=A1AAAA1a2aaaa(a2)AAa(aa3)a(a34)AAa5a4aaaa5	OPLS-2005	9.063	[*]c1ccc(C)n1C	-6.230	-7.407	-54.034
201	DC-201	A=A1AAAA1a2aaaa(a2)AAa(aa3)a(a34)AAa5a4aaaa5	OPLS-2005	9.291	[*]CC(C)CCC	-6.166	-7.350	-53.424
202	DC-202	A=A1AAAA1a2aaaa(a2)AAa(aa3)a(a34)AAa5a4aaaa5	OPLS-2005	9.068	[*]N(C)C1(C)CCC1	-6.116	-7.308	-40.607

203	DC-203	A=A1AAAA1a2aaaa(a2)AAa(aa3)a(a34)AAa5a4aaaa5	OPLS-2005	9.513	[*]N(C1)CC1C(C)C	-6.078	-7.255	-45.552
204	DC-204	A=A1AAAA1a2aaaa(a2)AAa(aa3)a(a34)AAa5a4aaaa5	OPLS-2005	9.138	[*]CCC1(F)CNC1	-6.009	-6.114	-40.800
205	DC-205	A=A1AAAA1a2aaaa(a2)AAa(aa3)a(a34)AAa5a4aaaa5	OPLS-2005	9.286	[*]N(C1)CC1(C)OC	-5.939	-7.517	-40.598
206	DC-206	A=A1AAAA1a2aaaa(a2)AAa(aa3)a(a34)AAa5a4aaaa5	OPLS-2005	9.215	[*]N(C1)CC[C@@H]1OC	-5.918	-6.005	-22.978
207	DC-207	A=A1AAAA1a2aaaa(a2)AAa(aa3)a(a34)AAa5a4aaaa5	OPLS-2005	9.137	[*]C[C@H](O)CC(C)C	-5.858	-5.950	-36.057
208	DC-208	A=A1AAAA1a2aaaa(a2)AAa(aa3)a(a34)AAa5a4aaaa5	OPLS-2005	9.193	[*]N(C1)CCCC1O	-5.633	-6.820	-45.382
209	DC-209	A=A1AAAA1a2aaaa(a2)AAa(aa3)a(a34)AAa5a4aaaa5	OPLS-2005	9.255	[*]N(C1)CC1N(C)C	-5.602	-5.823	-42.692
210	DC-210	A=A1AAAA1a2aaaa(a2)AAa(aa3)a(a34)AAa5a4aaaa5	OPLS-2005	9.255	[*]N(C1)CC1N(C)C	-5.433	-7.616	-47.453
211	DC-211	A=A1AAAA1a2aaaa(a2)AAa(aa3)a(a34)AAa5a4aaaa5	OPLS-2005	9.175	[*]CC[C@H]1CCNC1	-5.334	-6.517	-52.457
212	DC-212	A=A1AAAA1a2aaaa(a2)AAa(aa3)a(a34)AAa5a4aaaa5	OPLS-2005	9.021	[*]CC1(C)[C@H](O)CC1	-5.332	-7.994	-47.483
213	DC-213	A=A1AAAA1a2aaaa(a2)AAa(aa3)a(a34)AAa5a4aaaa5	OPLS-2005	9.096	[*]N(C1)CC1(C)CO	-5.295	-5.405	-44.363
214	DC-214	A=A1AAAA1a2aaaa(a2)AAa(aa3)a(a34)AAa5a4aaaa5	OPLS-2005	9.009	[*]N1CCN(C)CC1	-5.294	-6.397	-38.575
215	DC-215	A=A1AAAA1a2aaaa(a2)AAa(aa3)a(a34)AAa5a4aaaa5	OPLS-2005	9.118	[*]NCC1(F)CCC1	-5.285	-7.775	-49.254
216	DC-216	A=A1AAAA1a2aaaa(a2)AAa(aa3)a(a34)AAa5a4aaaa5	OPLS-2005	9.171	O1CCN([*])C[C@@H]1C	-5.236	-6.413	-52.765
217	DC-217	A=A1AAAA1a2aaaa(a2)AAa(aa3)a(a34)AAa5a4aaaa5	OPLS-2005	9.177	[*]CC[C@@H]1C[C@@H](C1)N	-5.233	-6.417	-42.962
218	DC-218	A=A1AAAA1a2aaaa(a2)AAa(aa3)a(a34)AAa5a4aaaa5	OPLS-2005	9.345	[*]N(C1)CC1(C)C	-5.101	-7.824	-47.170
219	DC-219	A=A1AAAA1a2aaaa(a2)AAa(aa3)a(a34)AAa5a4aaaa5	OPLS-2005	9.515	[*]N1CCCC1CC	-5.085	-7.362	-47.161
220	DC-220	A=A1AAAA1a2aaaa(a2)AAa(aa3)a(a34)AAa5a4aaaa5	OPLS-2005	9.372	[*]N1CCCC1C	-5.036	-5.132	-33.415
221	DC-221	A=A1AAAA1a2aaaa(a2)AAa(aa3)a(a34)AAa5a4aaaa5	OPLS-2005	9.210	[*]N(C1)CC1(F)C	-4.973	-7.103	-47.146
222	DC-222	A=A1AAAA1a2aaaa(a2)AAa(aa3)a(a34)AAa5a4aaaa5	OPLS-2005	9.343	CCN([*])C1(C)CC1	-4.694	-5.871	-40.636
223	DC-223	A=A1AAAA1a2aaaa(a2)AAa(aa3)a(a34)AAa5a4aaaa5	OPLS-2005	9.138	[*]CCC1(F)CNC1	-3.971	-6.291	-38.024
224	DC-224	A=A1AAAA1a2aaaa(a2)AAa(aa3)a(a34)AAa5a4aaaa5	OPLS-2005	9.057	[*]N(C1)CC1[C@H](C)O	-3.874	-6.403	-43.889
225	DC-225	A=A1AAAA1a2aaaa(a2)AAa(aa3)a(a34)AAa5a4aaaa5	OPLS-2005	9.217	[*]CC(C)(C)CCO	-3.735	-6.341	-42.174

226	DC-226	A=A1AAAA1a2aaaa(a2)AAa(aa3)a(a34)AAa5a4aaaa5	OPLS-2005	9.291	[*]CC(C)CCC	-3.528	-6.172	-39.683
227	DC-227	A=A1AAAA1a2aaaa(a2)AAa(aa3)a(a34)AAa5a4aaaa5	OPLS-2005	9.358	[*]N1CCCC1(C)C	-2.840	-5.316	-34.975
228	DC-228	A=A1AAAA1a2aaaa(a2)AAa(aa3)a(a34)AAa5a4aaaa5	OPLS-2005	9.328	[*]N(C1)CC1(C#N)C	-2.788	-3.966	-36.273
229	DC-229	A=A1AAAA1a2aaaa(a2)AAa(aa3)a(a34)AAa5a4aaaa5	OPLS-2005	9.068	[*]N(C)C1(C)CCC1	-2.636	-4.843	-37.445

Table S4B: The molecular docking results for 371 designed compounds using Glide package in Schrodinger suite. All the compounds are aligned based on the docking results and functional group modifications at 6th position of Pyrimidine moiety especially in hydrophobic and steric nature.

S.NO	Designed Compounds	Core SMARTS	Force Field	Predicted Activity 7	R-Group Modification	Docking Score kcal/mol	Glide Score kcal/mol	Glide Energy kcal/mol
1	DC-230	A=A1AAAA1a2aaaa(a2)AAa(aa3)a(a34)AAa5a4aaaa5	OPLS-2005	9.023	[*]CNC1(CN)CC1	-10.008	-10.127	-57.480
2	DC-231	A=A1AAAA1a2aaaa(a2)AAa(aa3)a(a34)AAa5a4aaaa5	OPLS-2005	9.065	[*]N(C1)CC1CN	-9.768	-10.052	-56.382
3	DC-232	A=A1AAAA1a2aaaa(a2)AAa(aa3)a(a34)AAa5a4aaaa5	OPLS-2005	9.031	[*]CN(C)CC(C)O	-9.733	-9.951	-58.377
4	DC-233	A=A1AAAA1a2aaaa(a2)AAa(aa3)a(a34)AAa5a4aaaa5	OPLS-2005	9.115	[*]N(C1)CC1(O)CC	-9.682	-10.050	-51.031
5	DC-234	A=A1AAAA1a2aaaa(a2)AAa(aa3)a(a34)AAa5a4aaaa5	OPLS-2005	9.148	[*]OC1(CC)COC1	-9.276	-9.403	-51.702
6	DC-235	A=A1AAAA1a2aaaa(a2)AAa(aa3)a(a34)AAa5a4aaaa5	OPLS-2005	9.103	[*]CC1(CN)CCC1	-9.264	-9.387	-54.858
7	DC-236	A=A1AAAA1a2aaaa(a2)AAa(aa3)a(a34)AAa5a4aaaa5	OPLS-2005	9.262	C1CN([*])CCC1N	-9.255	-9.465	-54.710
8	DC-237	A=A1AAAA1a2aaaa(a2)AAa(aa3)a(a34)AAa5a4aaaa5	OPLS-2005	9.055	[*]CC1(N)CCOC1	-9.190	-10.397	-57.609
9	DC-238	A=A1AAAA1a2aaaa(a2)AAa(aa3)a(a34)AAa5a4aaaa5	OPLS-2005	9.086	[*]CC(F)(C)CN	-9.176	-9.311	-54.866
10	DC-239	A=A1AAAA1a2aaaa(a2)AAa(aa3)a(a34)AAa5a4aaaa5	OPLS-2005	9.150	[*]c1c(C)csc1	-9.111	-9.217	-42.262
11	DC-240	A=A1AAAA1a2aaaa(a2)AAa(aa3)a(a34)AAa5a4aaaa5	OPLS-2005	9.342	[*]N(C)C(C)CN	-9.055	-9.339	-55.444
12	DC-241	A=A1AAAA1a2aaaa(a2)AAa(aa3)a(a34)AA	OPLS-2005	9.202	[*]N1CC[C@@H]1CN	-9.041	-9.277	-50.074

		a5a4aaaa5						
13	DC-242	A=A1AAAA1a2aaaa(a2)AAa(aa3)a(a34)AAa5a4aaaa5	OPLS-2005	9.102	[*]OC1([C@H](C)N)CC1	-9.041	-9.543	-56.119
14	DC-243	A=A1AAAA1a2aaaa(a2)AAa(aa3)a(a34)AAa5a4aaaa5	OPLS-2005	9.004	[*]OC(C)CCN	-9.018	-9.847	-54.211
15	DC-244	A=A1AAAA1a2aaaa(a2)AAa(aa3)a(a34)AAa5a4aaaa5	OPLS-2005	9.026	[*]CC(F)(C)CO	-8.975	-9.172	-39.046
16	DC-245	A=A1AAAA1a2aaaa(a2)AAa(aa3)a(a34)AAa5a4aaaa5	OPLS-2005	9.001	[*]OCCC1(N)CC1	-8.906	-9.252	-52.535
17	DC-246	A=A1AAAA1a2aaaa(a2)AAa(aa3)a(a34)AAa5a4aaaa5	OPLS-2005	9.166	[*]c1c(C)ncnc1	-8.901	-8.988	-55.187
18	DC-247	A=A1AAAA1a2aaaa(a2)AAa(aa3)a(a34)AAa5a4aaaa5	OPLS-2005	9.166	[*]N1CCC1C	-8.857	-11.515	-52.982
19	DC-248	A=A1AAAA1a2aaaa(a2)AAa(aa3)a(a34)AAa5a4aaaa5	OPLS-2005	9.147	[*]c1c(C)scc1	-8.828	-8.934	-51.976
20	DC-249	A=A1AAAA1a2aaaa(a2)AAa(aa3)a(a34)AAa5a4aaaa5	OPLS-2005	9.086	[*]CC(F)(C)CN	-8.797	-10.022	-58.299
21	DC-250	A=A1AAAA1a2aaaa(a2)AAa(aa3)a(a34)AAa5a4aaaa5	OPLS-2005	9.102	[*]OC1([C@H](C)N)CC1	-8.739	-9.961	-55.325
22	DC-251	A=A1AAAA1a2aaaa(a2)AAa(aa3)a(a34)AAa5a4aaaa5	OPLS-2005	9.090	[*]CC(N)C(C)(C)O	-8.732	-8.885	-53.584
23	DC-252	A=A1AAAA1a2aaaa(a2)AAa(aa3)a(a34)AAa5a4aaaa5	OPLS-2005	9.031	[*]n(c1)ccc1C#N	-8.655	-8.742	-40.003
24	DC-253	A=A1AAAA1a2aaaa(a2)AAa(aa3)a(a34)AAa5a4aaaa5	OPLS-2005	9.101	[*]c1c(O)nncc1	-8.652	-8.755	-38.111
25	DC-254	A=A1AAAA1a2aaaa(a2)AAa(aa3)a(a34)AAa5a4aaaa5	OPLS-2005	9.103	[*]CC1(CN)CCC1	-8.649	-9.859	-58.966
26	DC-255	A=A1AAAA1a2aaaa(a2)AAa(aa3)a(a34)AAa5a4aaaa5	OPLS-2005	9.203	[*]OC(C)(CC)CN	-8.613	-9.101	-53.103
27	DC-256	A=A1AAAA1a2aaaa(a2)AAa(aa3)a(a34)AAa5a4aaaa5	OPLS-2005	9.131	[*]c1cc(N)ncc1	-8.612	-8.708	-38.435
28	DC-257	A=A1AAAA1a2aaaa(a2)AAa(aa3)a(a34)AAa5a4aaaa5	OPLS-2005	9.204	[*]N(C1)CC[C@@H]1CN	-8.586	-8.802	-40.928
29	DC-258	A=A1AAAA1a2aaaa(a2)AAa(aa3)a(a34)AAa5a4aaaa5	OPLS-2005	9.066	[*]OCC1(C)CCC1	-8.575	-9.598	-55.196
30	DC-259	A=A1AAAA1a2aaaa(a2)AAa(aa3)a(a34)AAa5a4aaaa5	OPLS-2005	9.096	[*]CC1(C(C)N)CC1	-8.546	-9.751	-56.081
31	DC-260	A=A1AAAA1a2aaaa(a2)AAa(aa3)a(a34)AAa5a4aaaa5	OPLS-2005	9.123	[*]CC1(NC)CCC1	-8.526	-9.703	-56.736
32	DC-261	A=A1AAAA1a2aaaa(a2)AAa(aa3)a(a34)AAa5a4aaaa5	OPLS-2005	9.028	[*]CC(C)CNC	-8.520	-8.632	-33.223
33	DC-262	A=A1AAAA1a2aaaa(a2)AAa(aa3)a(a34)AAa5a4aaaa5	OPLS-2005	9.096	[*]CC1(C(C)N)CC1	-8.474	-8.589	-45.740
34	DC-263	A=A1AAAA1a2aaaa(a2)AAa(aa3)a(a34)AAa5a4aaaa5	OPLS-2005	9.149	[*]CC(C)(CN)CO	-8.471	-8.609	-51.124

35	DC-264	A=A1AAAA1a2aaaa(a2)AAa(aa3)a(a34)AAa5a4aaaa5	OPLS-2005	9.261	[*]c1c(C)ccn1C	-8.461	-8.557	-49.260
36	DC-265	A=A1AAAA1a2aaaa(a2)AAa(aa3)a(a34)AAa5a4aaaa5	OPLS-2005	9.229	[*]c1cccn1CC	-8.430	-8.527	-50.035
37	DC-266	A=A1AAAA1a2aaaa(a2)AAa(aa3)a(a34)AAa5a4aaaa5	OPLS-2005	9.224	[*]n1c(C)ncc1C	-8.425	-9.628	-58.083
38	DC-267	A=A1AAAA1a2aaaa(a2)AAa(aa3)a(a34)AAa5a4aaaa5	OPLS-2005	9.091	[*]c1c(CO)[nH]nc1	-8.422	-9.609	-52.300
39	DC-268	A=A1AAAA1a2aaaa(a2)AAa(aa3)a(a34)AAa5a4aaaa5	OPLS-2005	9.394	[*]N1CCCC1CC	-8.411	-8.696	-46.282
40	DC-269	A=A1AAAA1a2aaaa(a2)AAa(aa3)a(a34)AAa5a4aaaa5	OPLS-2005	9.064	[*]c1c(O)scc1	-8.381	-8.707	-43.912
41	DC-270	A=A1AAAA1a2aaaa(a2)AAa(aa3)a(a34)AAa5a4aaaa5	OPLS-2005	9.128	[*]N1CCC1CCO	-8.362	-9.345	-51.611
42	DC-271	A=A1AAAA1a2aaaa(a2)AAa(aa3)a(a34)AAa5a4aaaa5	OPLS-2005	9.261	[*]c1c(C)ccn1C	-8.346	-9.533	-56.363
43	DC-272	A=A1AAAA1a2aaaa(a2)AAa(aa3)a(a34)AAa5a4aaaa5	OPLS-2005	9.039	[*]CC1(O)CCC1	-8.302	-8.661	-51.540
44	DC-273	A=A1AAAA1a2aaaa(a2)AAa(aa3)a(a34)AAa5a4aaaa5	OPLS-2005	9.214	[*]c1c(F)cccc1	-8.272	-8.367	-40.669
45	DC-274	A=A1AAAA1a2aaaa(a2)AAa(aa3)a(a34)AAa5a4aaaa5	OPLS-2005	9.008	[*]CC(C)NC	-8.246	-8.333	-37.878
46	DC-275	A=A1AAAA1a2aaaa(a2)AAa(aa3)a(a34)AAa5a4aaaa5	OPLS-2005	9.188	[*]c1cc(N)ccc1	-8.237	-8.335	-28.615
47	DC-276	A=A1AAAA1a2aaaa(a2)AAa(aa3)a(a34)AAa5a4aaaa5	OPLS-2005	9.148	[*]CC(C)(CN)NC	-8.214	-8.891	-54.646
48	DC-277	A=A1AAAA1a2aaaa(a2)AAa(aa3)a(a34)AAa5a4aaaa5	OPLS-2005	9.090	[*]O[C@@H](C)C1CCC1	-8.116	-8.271	-46.670
49	DC-278	A=A1AAAA1a2aaaa(a2)AAa(aa3)a(a34)AAa5a4aaaa5	OPLS-2005	9.068	[*]N(C1)CC1C	-8.109	-8.471	-47.722
50	DC-279	A=A1AAAA1a2aaaa(a2)AAa(aa3)a(a34)AAa5a4aaaa5	OPLS-2005	9.182	[*]CC(C)(O)C(C)C	-8.047	-8.406	-55.340
51	DC-280	A=A1AAAA1a2aaaa(a2)AAa(aa3)a(a34)AAa5a4aaaa5	OPLS-2005	9.037	[*]n1cccc1O	-8.005	-8.207	-51.616
52	DC-281	A=A1AAAA1a2aaaa(a2)AAa(aa3)a(a34)AAa5a4aaaa5	OPLS-2005	9.003	[*]NC1(C)CC(C1)O	-7.961	-8.334	-50.180
53	DC-282	A=A1AAAA1a2aaaa(a2)AAa(aa3)a(a34)AAa5a4aaaa5	OPLS-2005	9.193	[*]N1C[C@@H](O)C1(C)C	-7.946	-8.918	-37.085
54	DC-283	A=A1AAAA1a2aaaa(a2)AAa(aa3)a(a34)AAa5a4aaaa5	OPLS-2005	9.247	[*]N(C)C1(C)CC1	-7.929	-8.264	-45.671
55	DC-284	A=A1AAAA1a2aaaa(a2)AAa(aa3)a(a34)AAa5a4aaaa5	OPLS-2005	9.055	[*]N(C)C1CC1	-7.911	-9.343	-54.757
56	DC-285	A=A1AAAA1a2aaaa(a2)AAa(aa3)a(a34)AAa5a4aaaa5	OPLS-2005	9.149	[*]NC1(C(C)C)CC1	-7.881	-8.764	-50.196
57	DC-286	A=A1AAAA1a2aaaa(a2)AAa(aa3)a(a34)AAa5a4aaaa5	OPLS-2005	9.021	[*]N(C)CC1CCC1	-7.877	-8.955	-36.989

58	DC-287	A=A1AAAA1a2aaaa(a2)AAa(aa3)a(a34)AAa5a4aaaa5	OPLS-2005	9.043	[*]c1c(O)n(C)cc1	-7.844	-9.043	-35.405
59	DC-288	A=A1AAAA1a2aaaa(a2)AAa(aa3)a(a34)AAa5a4aaaa5	OPLS-2005	9.255	[*]N(C)C(C)CC	-7.826	-9.269	-48.030
60	DC-289	A=A1AAAA1a2aaaa(a2)AAa(aa3)a(a34)AAa5a4aaaa5	OPLS-2005	9.149	[*]CC(C)(CN)CO	-7.823	-9.049	-52.099
61	DC-290	A=A1AAAA1a2aaaa(a2)AAa(aa3)a(a34)AAa5a4aaaa5	OPLS-2005	9.202	[*]N1CC[C@@H]1CN	-7.817	-9.143	-52.387
62	DC-291	A=A1AAAA1a2aaaa(a2)AAa(aa3)a(a34)AAa5a4aaaa5	OPLS-2005	9.260	[*]OC(C)(C)C(C)N	-7.787	-8.439	-53.560
63	DC-292	A=A1AAAA1a2aaaa(a2)AAa(aa3)a(a34)AAa5a4aaaa5	OPLS-2005	9.132	[*]c1cccc(n1)O	-7.777	-7.864	-40.766
64	DC-293	A=A1AAAA1a2aaaa(a2)AAa(aa3)a(a34)AAa5a4aaaa5	OPLS-2005	9.184	[*]N(C)[C@H](C)C1CC1	-7.756	-8.148	-50.459
65	DC-294	A=A1AAAA1a2aaaa(a2)AAa(aa3)a(a34)AAa5a4aaaa5	OPLS-2005	9.136	[*]c1cnc(C)n1C	-7.744	-9.636	-40.706
66	DC-295	A=A1AAAA1a2aaaa(a2)AAa(aa3)a(a34)AAa5a4aaaa5	OPLS-2005	9.023	[*]CC(C)(O)CCN	-7.737	-9.535	-54.292
67	DC-296	A=A1AAAA1a2aaaa(a2)AAa(aa3)a(a34)AAa5a4aaaa5	OPLS-2005	9.150	[*]c1c(C)csc1	-7.698	-8.894	-52.470
68	DC-297	A=A1AAAA1a2aaaa(a2)AAa(aa3)a(a34)AAa5a4aaaa5	OPLS-2005	9.209	[*]N(C1)CC[C@@H]1OC	-7.661	-10.361	-35.674
69	DC-298	A=A1AAAA1a2aaaa(a2)AAa(aa3)a(a34)AAa5a4aaaa5	OPLS-2005	9.076	[*]N(C1)CC1CCN	-7.655	-10.026	-56.002
70	DC-299	A=A1AAAA1a2aaaa(a2)AAa(aa3)a(a34)AAa5a4aaaa5	OPLS-2005	9.146	[*]c1cccn1C	-7.634	-8.820	-53.870
71	DC-300	A=A1AAAA1a2aaaa(a2)AAa(aa3)a(a34)AAa5a4aaaa5	OPLS-2005	9.039	[*]CC1(O)CCC1	-7.619	-9.359	-52.821
72	DC-301	A=A1AAAA1a2aaaa(a2)AAa(aa3)a(a34)AAa5a4aaaa5	OPLS-2005	9.157	[*]CC(C)(CN)OC	-7.613	-8.851	-53.944
73	DC-302	A=A1AAAA1a2aaaa(a2)AAa(aa3)a(a34)AAa5a4aaaa5	OPLS-2005	9.367	[*]N(C)C(C)(C)CN	-7.599	-7.868	-49.093
74	DC-303	A=A1AAAA1a2aaaa(a2)AAa(aa3)a(a34)AAa5a4aaaa5	OPLS-2005	9.220	[*]OC(C(C)C)CN	-7.590	-8.881	-48.005
75	DC-304	A=A1AAAA1a2aaaa(a2)AAa(aa3)a(a34)AAa5a4aaaa5	OPLS-2005	9.103	[*]CC1(CN)CCC1	-7.577	-9.354	-52.727
76	DC-305	A=A1AAAA1a2aaaa(a2)AAa(aa3)a(a34)AAa5a4aaaa5	OPLS-2005	9.036	[*]NC1(C)CCC1	-7.562	-9.627	-53.544
77	DC-306	A=A1AAAA1a2aaaa(a2)AAa(aa3)a(a34)AAa5a4aaaa5	OPLS-2005	9.023	[*]CC(C)(O)CCN	-7.548	-8.653	-44.385
78	DC-307	A=A1AAAA1a2aaaa(a2)AAa(aa3)a(a34)AAa5a4aaaa5	OPLS-2005	9.152	[*]c1c(C)c[nH]c1	-7.532	-8.719	-38.642
79	DC-308	A=A1AAAA1a2aaaa(a2)AAa(aa3)a(a34)AAa5a4aaaa5	OPLS-2005	9.230	O1CCN([*])C[C@@H]1C	-7.526	-7.640	-12.461
80	DC-309	A=A1AAAA1a2aaaa(a2)AAa(aa3)a(a34)AAa5a4aaaa5	OPLS-2005	9.254	[*]c1c(C)nccc1	-7.509	-8.693	-36.433

81	DC-310	A=A1AAAA1a2aaaa(a2)AAa(aa3)a(a34)AAa5a4aaaa5	OPLS-2005	9.158	[*]N(C)CC(C)(C)O	-7.496	-8.398	-51.406
82	DC-311	A=A1AAAA1a2aaaa(a2)AAa(aa3)a(a34)AAa5a4aaaa5	OPLS-2005	9.096	[*]NC1(CC1)CCC	-7.421	-8.880	-50.871
83	DC-312	A=A1AAAA1a2aaaa(a2)AAa(aa3)a(a34)AAa5a4aaaa5	OPLS-2005	9.055	[*]N(C)C1CC1	-7.417	-7.765	-43.032
84	DC-313	A=A1AAAA1a2aaaa(a2)AAa(aa3)a(a34)AAa5a4aaaa5	OPLS-2005	9.155	CCN([*])CC	-7.415	-7.716	-35.843
85	DC-314	A=A1AAAA1a2aaaa(a2)AAa(aa3)a(a34)AAa5a4aaaa5	OPLS-2005	9.011	[*]CC1(C)C(C1)N	-7.393	-8.631	-53.638
86	DC-315	A=A1AAAA1a2aaaa(a2)AAa(aa3)a(a34)AAa5a4aaaa5	OPLS-2005	9.001	[*]OC(C)CC1CC1	-7.378	-8.632	-51.010
87	DC-316	A=A1AAAA1a2aaaa(a2)AAa(aa3)a(a34)AAa5a4aaaa5	OPLS-2005	9.253	[*]N(C)CC1(N)CC1	-7.371	-8.269	-53.104
88	DC-317	A=A1AAAA1a2aaaa(a2)AAa(aa3)a(a34)AAa5a4aaaa5	OPLS-2005	9.096	[*]c1c(C)[nH]cn1	-7.362	-8.714	-39.428
89	DC-318	A=A1AAAA1a2aaaa(a2)AAa(aa3)a(a34)AAa5a4aaaa5	OPLS-2005	9.055	[*]N(C)C1CC1	-7.361	-8.265	-45.961
90	DC-319	A=A1AAAA1a2aaaa(a2)AAa(aa3)a(a34)AAa5a4aaaa5	OPLS-2005	9.174	[*]NC(C)(C)CCC	-7.342	-8.294	-48.719
91	DC-320	A=A1AAAA1a2aaaa(a2)AAa(aa3)a(a34)AAa5a4aaaa5	OPLS-2005	9.139	[*]N(C)C1CCC1	-7.337	-7.733	-42.572
92	DC-321	A=A1AAAA1a2aaaa(a2)AAa(aa3)a(a34)AAa5a4aaaa5	OPLS-2005	9.148	[*]CC(C)(CN)NC	-7.335	-9.082	-53.135
93	DC-322	A=A1AAAA1a2aaaa(a2)AAa(aa3)a(a34)AAa5a4aaaa5	OPLS-2005	9.151	c1nn([*])c(C)c1C	-7.330	-8.507	-23.802
94	DC-323	A=A1AAAA1a2aaaa(a2)AAa(aa3)a(a34)AAa5a4aaaa5	OPLS-2005	9.040	[*]N(C)CCO	-7.321	-7.659	-39.345
95	DC-324	A=A1AAAA1a2aaaa(a2)AAa(aa3)a(a34)AAa5a4aaaa5	OPLS-2005	9.023	[*]CC(F)(C)C	-7.314	-8.550	-52.490
96	DC-325	A=A1AAAA1a2aaaa(a2)AAa(aa3)a(a34)AAa5a4aaaa5	OPLS-2005	9.016	[*]c1ccsn1	-7.288	-8.482	-53.073
97	DC-326	A=A1AAAA1a2aaaa(a2)AAa(aa3)a(a34)AAa5a4aaaa5	OPLS-2005	9.155	CCN([*])CC	-7.268	-8.660	-50.466
98	DC-327	A=A1AAAA1a2aaaa(a2)AAa(aa3)a(a34)AAa5a4aaaa5	OPLS-2005	9.107	[*]N(C)C1CC(C1)O	-7.263	-8.348	-33.240
99	DC-328	A=A1AAAA1a2aaaa(a2)AAa(aa3)a(a34)AAa5a4aaaa5	OPLS-2005	9.166	[*]N1CCC1C	-7.255	-8.236	-49.004
100	DC-329	A=A1AAAA1a2aaaa(a2)AAa(aa3)a(a34)AAa5a4aaaa5	OPLS-2005	9.104	[*]N(C)C1COC1	-7.254	-7.585	-29.873
101	DC-330	A=A1AAAA1a2aaaa(a2)AAa(aa3)a(a34)AAa5a4aaaa5	OPLS-2005	9.213	[*]N(C1)CC[C@@H]1C#N	-7.252	-8.251	-48.339
102	DC-331	A=A1AAAA1a2aaaa(a2)AAa(aa3)a(a34)AAa5a4aaaa5	OPLS-2005	9.183	[*]CC(F)(C)CC	-7.251	-7.407	-39.997
103	DC-332	A=A1AAAA1a2aaaa(a2)AAa(aa3)a(a34)AAa5a4aaaa5	OPLS-2005	9.047	[*]N(C1)CC1N	-7.240	-9.545	-56.378

104	DC-333	A=A1AAAA1a2aaaa(a2)AAa(aa3)a(a34)AAa5a4aaaa5	OPLS-2005	9.005	[*][N-]C1CCNC1	-7.235	-7.730	-31.081
105	DC-334	A=A1AAAA1a2aaaa(a2)AAa(aa3)a(a34)AAa5a4aaaa5	OPLS-2005	9.047	[*]N(C1)CC1N	-7.196	-7.623	-38.089
106	DC-335	A=A1AAAA1a2aaaa(a2)AAa(aa3)a(a34)AAa5a4aaaa5	OPLS-2005	9.297	[*]N(C)C1(CN)CC1	-7.186	-8.422	-51.568
107	DC-336	A=A1AAAA1a2aaaa(a2)AAa(aa3)a(a34)AAa5a4aaaa5	OPLS-2005	9.167	[*]N1CCCCC1=O	-7.184	-7.276	-18.575
108	DC-337	A=A1AAAA1a2aaaa(a2)AAa(aa3)a(a34)AAa5a4aaaa5	OPLS-2005	9.082	[*]N[C@@H](C)C1CCC1	-7.171	-7.553	-47.167
109	DC-338	A=A1AAAA1a2aaaa(a2)AAa(aa3)a(a34)AAa5a4aaaa5	OPLS-2005	9.166	[*]N1CCC1C	-7.123	-7.424	-43.694
110	DC-339	A=A1AAAA1a2aaaa(a2)AAa(aa3)a(a34)AAa5a4aaaa5	OPLS-2005	9.011	[*]N(C)CC[NH-]	-7.115	-8.001	-35.928
111	DC-340	A=A1AAAA1a2aaaa(a2)AAa(aa3)a(a34)AAa5a4aaaa5	OPLS-2005	9.065	[*]NC(C)CC	-7.109	-9.108	-52.793
112	DC-341	A=A1AAAA1a2aaaa(a2)AAa(aa3)a(a34)AAa5a4aaaa5	OPLS-2005	9.255	C1CN([*])CCC1O	-7.090	-7.364	-17.044
113	DC-342	A=A1AAAA1a2aaaa(a2)AAa(aa3)a(a34)AAa5a4aaaa5	OPLS-2005	9.285	[*]N1CC[C@@H]1C(C)C	-7.088	-8.471	-48.580
114	DC-343	A=A1AAAA1a2aaaa(a2)AAa(aa3)a(a34)AAa5a4aaaa5	OPLS-2005	9.086	[*]CC(F)(C)CN	-7.078	-9.060	-53.611
115	DC-344	A=A1AAAA1a2aaaa(a2)AAa(aa3)a(a34)AAa5a4aaaa5	OPLS-2005	9.170	[*]c1ccccc1	-7.073	-7.168	-49.469
116	DC-345	A=A1AAAA1a2aaaa(a2)AAa(aa3)a(a34)AAa5a4aaaa5	OPLS-2005	9.181	[*]n1cccc1CC	-7.066	-8.243	-49.828
117	DC-346	A=A1AAAA1a2aaaa(a2)AAa(aa3)a(a34)AAa5a4aaaa5	OPLS-2005	9.043	[*]N[C@@H](C)CCO	-7.055	-9.048	-54.427
118	DC-347	A=A1AAAA1a2aaaa(a2)AAa(aa3)a(a34)AAa5a4aaaa5	OPLS-2005	9.012	[*]CC(CC)NC	-7.053	-8.230	-51.582
119	DC-348	A=A1AAAA1a2aaaa(a2)AAa(aa3)a(a34)AAa5a4aaaa5	OPLS-2005	9.174	[*]N1CCOCC1	-7.046	-7.157	-19.644
120	DC-349	A=A1AAAA1a2aaaa(a2)AAa(aa3)a(a34)AAa5a4aaaa5	OPLS-2005	9.512	[*]N1CCCC1(C)C	-7.031	-8.403	-41.467
121	DC-350	A=A1AAAA1a2aaaa(a2)AAa(aa3)a(a34)AAa5a4aaaa5	OPLS-2005	9.297	[*]N(C)C1(CN)CC1	-7.018	-9.504	-52.871
122	DC-351	A=A1AAAA1a2aaaa(a2)AAa(aa3)a(a34)AAa5a4aaaa5	OPLS-2005	9.118	[*]OC1(CC)CCC1	-7.014	-8.246	-48.809
123	DC-352	A=A1AAAA1a2aaaa(a2)AAa(aa3)a(a34)AAa5a4aaaa5	OPLS-2005	9.067	[*]NC(C)(C)CO	-7.011	-9.013	-52.757
124	DC-353	A=A1AAAA1a2aaaa(a2)AAa(aa3)a(a34)AAa5a4aaaa5	OPLS-2005	9.023	[*]CNC1(CN)CC1	-6.998	-9.192	-33.322
125	DC-354	A=A1AAAA1a2aaaa(a2)AAa(aa3)a(a34)AAa5a4aaaa5	OPLS-2005	9.030	[*]CC(C)(O)CC	-6.970	-8.656	-35.737
126	DC-355	A=A1AAAA1a2aaaa(a2)AAa(aa3)a(a34)AAa5a4aaaa5	OPLS-2005	9.164	[*]N(C)CC(C)(C)N	-6.934	-8.158	-48.162

127	DC-356	A=A1AAAA1a2aaaa(a2)AAa(aa3)a(a34)AAa5a4aaaa5	OPLS-2005	9.131	[*]N(C)CC(C)N	-6.930	-8.152	-49.902
128	DC-357	A=A1AAAA1a2aaaa(a2)AAa(aa3)a(a34)AAa5a4aaaa5	OPLS-2005	9.072	[*]NC1(CC)CC1	-6.903	-7.791	-45.522
129	DC-358	A=A1AAAA1a2aaaa(a2)AAa(aa3)a(a34)AAa5a4aaaa5	OPLS-2005	9.193	[*]N1C[C@@H](O)C1(C)C	-6.900	-8.266	-47.494
130	DC-359	A=A1AAAA1a2aaaa(a2)AAa(aa3)a(a34)AAa5a4aaaa5	OPLS-2005	9.119	[*]c1c(C)csn1	-6.899	-9.025	-47.667
131	DC-360	A=A1AAAA1a2aaaa(a2)AAa(aa3)a(a34)AAa5a4aaaa5	OPLS-2005	9.147	[*]N1CCC1C#N	-6.892	-7.278	-38.933
132	DC-361	A=A1AAAA1a2aaaa(a2)AAa(aa3)a(a34)AAa5a4aaaa5	OPLS-2005	9.190	[*]c1c(C#N)cccn1	-6.884	-6.978	-49.874
133	DC-362	A=A1AAAA1a2aaaa(a2)AAa(aa3)a(a34)AAa5a4aaaa5	OPLS-2005	9.080	[*]N(C)C(=O)C(C)C	-6.873	-6.960	-36.236
134	DC-363	A=A1AAAA1a2aaaa(a2)AAa(aa3)a(a34)AAa5a4aaaa5	OPLS-2005	9.209	[*]N(C)[C@H](C1)C[C@H]1C	-6.864	-7.252	-44.215
135	DC-364	A=A1AAAA1a2aaaa(a2)AAa(aa3)a(a34)AAa5a4aaaa5	OPLS-2005	9.046	[*]N(C)CC(F)F	-6.799	-7.758	-33.882
136	DC-365	A=A1AAAA1a2aaaa(a2)AAa(aa3)a(a34)AAa5a4aaaa5	OPLS-2005	9.054	[*]CC(C)(C)N	-6.796	-6.884	-42.360
137	DC-366	A=A1AAAA1a2aaaa(a2)AAa(aa3)a(a34)AAa5a4aaaa5	OPLS-2005	9.112	[*]CC1(CN)CNC1	-6.787	-7.986	-48.577
138	DC-367	A=A1AAAA1a2aaaa(a2)AAa(aa3)a(a34)AAa5a4aaaa5	OPLS-2005	9.119	[*]c1c(C)csn1	-6.745	-6.849	-31.221
139	DC-368	A=A1AAAA1a2aaaa(a2)AAa(aa3)a(a34)AAa5a4aaaa5	OPLS-2005	9.224	[*]n1c(C)ncc1C	-6.724	-6.837	-51.968
140	DC-369	A=A1AAAA1a2aaaa(a2)AAa(aa3)a(a34)AAa5a4aaaa5	OPLS-2005	9.034	[*]C[C@H]1C[C@@H](C1)CN	-6.720	-7.936	-42.455
141	DC-370	A=A1AAAA1a2aaaa(a2)AAa(aa3)a(a34)AAa5a4aaaa5	OPLS-2005	9.036	[*]NC1(C)CCC1	-6.704	-7.813	-48.631
142	DC-371	A=A1AAAA1a2aaaa(a2)AAa(aa3)a(a34)AAa5a4aaaa5	OPLS-2005	9.148	[*]c1c(C)[nH]cc1	-6.702	-7.889	-33.067
143	DC-372	A=A1AAAA1a2aaaa(a2)AAa(aa3)a(a34)AAa5a4aaaa5	OPLS-2005	9.201	[*]N1C[C@@H](N)C1(C)C	-6.664	-8.864	-54.134
144	DC-373	A=A1AAAA1a2aaaa(a2)AAa(aa3)a(a34)AAa5a4aaaa5	OPLS-2005	9.137	[*]CN(C)C(C)CN	-6.661	-6.763	-47.619
145	DC-374	A=A1AAAA1a2aaaa(a2)AAa(aa3)a(a34)AAa5a4aaaa5	OPLS-2005	9.005	[*][N-]C1CCNC1	-6.654	-8.845	-52.445
146	DC-375	A=A1AAAA1a2aaaa(a2)AAa(aa3)a(a34)AAa5a4aaaa5	OPLS-2005	9.182	[*]CC(C)(O)C(C)C	-6.650	-8.272	-36.398
147	DC-376	A=A1AAAA1a2aaaa(a2)AAa(aa3)a(a34)AAa5a4aaaa5	OPLS-2005	9.030	[*]N(C1)CC1O	-6.637	-8.640	-48.139
148	DC-377	A=A1AAAA1a2aaaa(a2)AAa(aa3)a(a34)AAa5a4aaaa5	OPLS-2005	9.011	[*]N(C)CC[NH-]	-6.633	-8.063	-46.598
149	DC-378	A=A1AAAA1a2aaaa(a2)AAa(aa3)a(a34)AAa5a4aaaa5	OPLS-2005	9.046	[*]NC1(CC1)CCO	-6.606	-7.499	-43.538

150	DC-379	A=A1AAAA1a2aaaa(a2)AAa(aa3)a(a34)AAa5a4aaaa5	OPLS-2005	9.287	[*]N(C)[C@@H](C1)C1(C)C	-6.599	-7.908	-45.074
151	DC-380	A=A1AAAA1a2aaaa(a2)AAa(aa3)a(a34)AAa5a4aaaa5	OPLS-2005	9.126	[*]NC(CC)C1CC1	-6.595	-7.567	-46.514
152	DC-381	A=A1AAAA1a2aaaa(a2)AAa(aa3)a(a34)AAa5a4aaaa5	OPLS-2005	9.115	[*]CC1(C)CC(C1)N	-6.564	-6.674	-43.506
153	DC-382	A=A1AAAA1a2aaaa(a2)AAa(aa3)a(a34)AAa5a4aaaa5	OPLS-2005	9.030	[*]N(C1)CC1O	-6.527	-6.865	-30.929
154	DC-383	A=A1AAAA1a2aaaa(a2)AAa(aa3)a(a34)AAa5a4aaaa5	OPLS-2005	9.166	[*]N1CCC1C	-6.519	-8.089	-37.362
155	DC-384	A=A1AAAA1a2aaaa(a2)AAa(aa3)a(a34)AAa5a4aaaa5	OPLS-2005	9.050	[*]NC1(C)CCCC1	-6.510	-7.738	-48.724
156	DC-385	A=A1AAAA1a2aaaa(a2)AAa(aa3)a(a34)AAa5a4aaaa5	OPLS-2005	9.057	[*]c1c(O)cco1	-6.497	-7.809	-26.705
157	DC-386	A=A1AAAA1a2aaaa(a2)AAa(aa3)a(a34)AAa5a4aaaa5	OPLS-2005	9.233	[*]N(C)CC(C)C	-6.486	-7.848	-30.625
158	DC-387	A=A1AAAA1a2aaaa(a2)AAa(aa3)a(a34)AAa5a4aaaa5	OPLS-2005	9.262	C1CN([*])CCC1N	-6.476	-7.996	-35.499
159	DC-388	A=A1AAAA1a2aaaa(a2)AAa(aa3)a(a34)AAa5a4aaaa5	OPLS-2005	9.212	[*]c1c(O)cccc1	-6.461	-6.556	-46.072
160	DC-389	A=A1AAAA1a2aaaa(a2)AAa(aa3)a(a34)AAa5a4aaaa5	OPLS-2005	9.131	[*]N(C)CC(C)N	-6.461	-7.693	-33.920
161	DC-390	A=A1AAAA1a2aaaa(a2)AAa(aa3)a(a34)AAa5a4aaaa5	OPLS-2005	9.070	c1[nH]n([*])c(=O)c1C	-6.440	-6.603	-47.038
162	DC-391	A=A1AAAA1a2aaaa(a2)AAa(aa3)a(a34)AAa5a4aaaa5	OPLS-2005	9.131	[*]N(C)CC(C)N	-6.434	-8.893	-56.015
163	DC-392	A=A1AAAA1a2aaaa(a2)AAa(aa3)a(a34)AAa5a4aaaa5	OPLS-2005	9.195	[*]N(C)C(C)C	-6.429	-7.338	-31.584
164	DC-393	A=A1AAAA1a2aaaa(a2)AAa(aa3)a(a34)AAa5a4aaaa5	OPLS-2005	9.063	[*]c1c(O)ccs1	-6.407	-9.024	-47.477
165	DC-394	A=A1AAAA1a2aaaa(a2)AAa(aa3)a(a34)AAa5a4aaaa5	OPLS-2005	9.104	[*]N(C)C1COC1	-6.385	-8.383	-48.238
166	DC-395	A=A1AAAA1a2aaaa(a2)AAa(aa3)a(a34)AAa5a4aaaa5	OPLS-2005	9.024	[*]c1cc(O)sc1	-6.385	-6.828	-52.067
167	DC-396	A=A1AAAA1a2aaaa(a2)AAa(aa3)a(a34)AAa5a4aaaa5	OPLS-2005	9.237	[*]c1c(N)cccc1	-6.375	-6.474	-42.274
168	DC-397	A=A1AAAA1a2aaaa(a2)AAa(aa3)a(a34)AAa5a4aaaa5	OPLS-2005	9.203	[*]OC(C)(CC)CN	-6.344	-8.227	-51.000
169	DC-398	A=A1AAAA1a2aaaa(a2)AAa(aa3)a(a34)AAa5a4aaaa5	OPLS-2005	9.189	[*]N1CCC1(C)C	-6.331	-7.302	-42.660
170	DC-399	A=A1AAAA1a2aaaa(a2)AAa(aa3)a(a34)AAa5a4aaaa5	OPLS-2005	9.061	[*]CC(C)C(=O)NC	-6.320	-8.060	-49.213
171	DC-400	A=A1AAAA1a2aaaa(a2)AAa(aa3)a(a34)AAa5a4aaaa5	OPLS-2005	9.034	[*]C[C@H]1C[C@@H](C1)CN	-6.305	-7.994	-24.898
172	DC-401	A=A1AAAA1a2aaaa(a2)AAa(aa3)a(a34)AAa5a4aaaa5	OPLS-2005	9.166	[*]N(C1)CC1(F)CN	-6.300	-6.597	-27.744

173	DC-402	A=A1AAAA1a2aaaa(a2)AAa(aa3)a(a34)AAa5a4aaaa5	OPLS-2005	9.247	[*]N(C)C1(C)CC1	-6.273	-7.163	-39.237
174	DC-403	A=A1AAAA1a2aaaa(a2)AAa(aa3)a(a34)AAa5a4aaaa5	OPLS-2005	9.287	[*]N(C)[C@@H](C1)C1(C)C	-6.247	-8.585	-54.262
175	DC-404	A=A1AAAA1a2aaaa(a2)AAa(aa3)a(a34)AAa5a4aaaa5	OPLS-2005	9.281	[*]CC(C)(CC)CN	-6.239	-8.078	-49.100
176	DC-405	A=A1AAAA1a2aaaa(a2)AAa(aa3)a(a34)AAa5a4aaaa5	OPLS-2005	9.133	[*]O[C@@H](C)C(C)(C)N	-6.229	-6.797	-38.984
177	DC-406	A=A1AAAA1a2aaaa(a2)AAa(aa3)a(a34)AAa5a4aaaa5	OPLS-2005	9.156	[*]c1c(O)cncc1	-6.223	-6.628	-52.405
178	DC-407	A=A1AAAA1a2aaaa(a2)AAa(aa3)a(a34)AAa5a4aaaa5	OPLS-2005	9.053	[*]c1cc(CN)c[nH]1	-6.209	-6.306	-45.833
179	DC-408	A=A1AAAA1a2aaaa(a2)AAa(aa3)a(a34)AAa5a4aaaa5	OPLS-2005	9.139	[*]N(C)C1CCC1	-6.195	-7.232	-38.257
180	DC-409	A=A1AAAA1a2aaaa(a2)AAa(aa3)a(a34)AAa5a4aaaa5	OPLS-2005	9.120	[*]NC(C)(C1)C1(C)C	-6.193	-7.668	-47.144
181	DC-410	A=A1AAAA1a2aaaa(a2)AAa(aa3)a(a34)AAa5a4aaaa5	OPLS-2005	9.155	CCN([*])CC	-6.167	-7.149	-29.745
182	DC-411	A=A1AAAA1a2aaaa(a2)AAa(aa3)a(a34)AAa5a4aaaa5	OPLS-2005	9.148	[*]CC(C)(CN)NC	-6.159	-8.604	-54.327
183	DC-412	A=A1AAAA1a2aaaa(a2)AAa(aa3)a(a34)AAa5a4aaaa5	OPLS-2005	9.088	[H]\N=c1\n([*])ccn1C	-6.157	-6.245	-47.055
184	DC-413	A=A1AAAA1a2aaaa(a2)AAa(aa3)a(a34)AAa5a4aaaa5	OPLS-2005	9.219	[*]NC1(CC)CCC1	-6.133	-8.195	-49.225
185	DC-414	A=A1AAAA1a2aaaa(a2)AAa(aa3)a(a34)AAa5a4aaaa5	OPLS-2005	9.072	[*]NC1(CC)CC1	-6.120	-7.545	-45.990
186	DC-415	A=A1AAAA1a2aaaa(a2)AAa(aa3)a(a34)AAa5a4aaaa5	OPLS-2005	9.140	[*]c1c(CN)occ1	-6.103	-7.940	-46.200
187	DC-416	A=A1AAAA1a2aaaa(a2)AAa(aa3)a(a34)AAa5a4aaaa5	OPLS-2005	9.095	[*]c1cc(N)cnc1	-6.097	-6.193	-2.438
188	DC-417	A=A1AAAA1a2aaaa(a2)AAa(aa3)a(a34)AAa5a4aaaa5	OPLS-2005	9.151	[*]c1c(CC)cc[nH]1	-6.038	-6.135	-25.782
189	DC-418	A=A1AAAA1a2aaaa(a2)AAa(aa3)a(a34)AAa5a4aaaa5	OPLS-2005	9.068	[*]n1ccnc1C	-6.025	-7.215	-36.720
190	DC-419	A=A1AAAA1a2aaaa(a2)AAa(aa3)a(a34)AAa5a4aaaa5	OPLS-2005	9.285	[*]N1CC[C@@H]1C(C)C	-5.992	-6.965	-45.214
191	DC-420	A=A1AAAA1a2aaaa(a2)AAa(aa3)a(a34)AAa5a4aaaa5	OPLS-2005	9.342	[*]N(C)C(C)CN	-5.991	-7.211	-47.905
192	DC-421	A=A1AAAA1a2aaaa(a2)AAa(aa3)a(a34)AAa5a4aaaa5	OPLS-2005	9.093	[*]c1c(F)cccn1	-5.949	-6.043	-48.692
193	DC-422	A=A1AAAA1a2aaaa(a2)AAa(aa3)a(a34)AAa5a4aaaa5	OPLS-2005	9.155	CCN([*])CC	-5.945	-8.603	-48.463
194	DC-423	A=A1AAAA1a2aaaa(a2)AAa(aa3)a(a34)AAa5a4aaaa5	OPLS-2005	9.026	[*]n1nccc1OC	-5.917	-6.005	-46.823
195	DC-424	A=A1AAAA1a2aaaa(a2)AAa(aa3)a(a34)AAa5a4aaaa5	OPLS-2005	9.030	[*]N(C1)CC1O	-5.914	-6.826	-25.482

196	DC-425	A=A1AAAA1a2aaaa(a2)AAa(aa3)a(a34)AAa5a4aaaa5	OPLS-2005	9.070	O=C1N([*])CCN1	-5.912	-6.307	-31.129
197	DC-426	A=A1AAAA1a2aaaa(a2)AAa(aa3)a(a34)AAa5a4aaaa5	OPLS-2005	9.048	[*]N[C@@H](C(C)C)CO	-5.899	-6.813	-42.085
198	DC-427	A=A1AAAA1a2aaaa(a2)AAa(aa3)a(a34)AAa5a4aaaa5	OPLS-2005	9.253	[*]N(C)CC1(N)CC1	-5.888	-7.169	-50.748
199	DC-428	A=A1AAAA1a2aaaa(a2)AAa(aa3)a(a34)AAa5a4aaaa5	OPLS-2005	9.078	[*]c1c(O)cn(n1)C	-5.874	-6.367	-47.382
200	DC-429	A=A1AAAA1a2aaaa(a2)AAa(aa3)a(a34)AAa5a4aaaa5	OPLS-2005	9.169	[*]OC1(C)CCNC1	-5.834	-6.899	-35.642
201	DC-430	A=A1AAAA1a2aaaa(a2)AAa(aa3)a(a34)AAa5a4aaaa5	OPLS-2005	9.186	[*]OC1(C)CCOC1	-5.831	-5.962	-27.117
202	DC-431	A=A1AAAA1a2aaaa(a2)AAa(aa3)a(a34)AAa5a4aaaa5	OPLS-2005	9.297	[*]N(C)C1(CN)CC1	-5.810	-7.057	-40.438
203	DC-432	A=A1AAAA1a2aaaa(a2)AAa(aa3)a(a34)AAa5a4aaaa5	OPLS-2005	9.044	[*]c1cn(cn1)CC	-5.799	-5.893	-43.196
204	DC-433	A=A1AAAA1a2aaaa(a2)AAa(aa3)a(a34)AAa5a4aaaa5	OPLS-2005	9.219	[*]NC1(CC)CCC1	-5.784	-6.174	-43.586
205	DC-434	A=A1AAAA1a2aaaa(a2)AAa(aa3)a(a34)AAa5a4aaaa5	OPLS-2005	9.329	[*]N(C)C1(CC)CC1	-5.783	-8.444	-54.375
206	DC-435	A=A1AAAA1a2aaaa(a2)AAa(aa3)a(a34)AAa5a4aaaa5	OPLS-2005	9.201	[*]OC(C)C1(C)CC1	-5.781	-5.930	-36.992
207	DC-436	A=A1AAAA1a2aaaa(a2)AAa(aa3)a(a34)AAa5a4aaaa5	OPLS-2005	9.146	[*]c1cccn1C	-5.773	-8.225	-46.232
208	DC-437	A=A1AAAA1a2aaaa(a2)AAa(aa3)a(a34)AAa5a4aaaa5	OPLS-2005	9.242	[*]c1c(C)ccnc1	-5.764	-5.858	-43.721
209	DC-438	A=A1AAAA1a2aaaa(a2)AAa(aa3)a(a34)AAa5a4aaaa5	OPLS-2005	9.345	CC(C)N([*])C1CC1	-5.749	-6.709	-48.854
210	DC-439	A=A1AAAA1a2aaaa(a2)AAa(aa3)a(a34)AAa5a4aaaa5	OPLS-2005	9.011	[*]c1c(O)cn[nH]1	-5.727	-6.323	-31.142
211	DC-440	A=A1AAAA1a2aaaa(a2)AAa(aa3)a(a34)AAa5a4aaaa5	OPLS-2005	9.244	[*]c1c(N)ccn1C	-5.702	-6.890	-44.051
212	DC-441	A=A1AAAA1a2aaaa(a2)AAa(aa3)a(a34)AAa5a4aaaa5	OPLS-2005	9.064	[*]c1c(O)scc1	-5.699	-7.765	-43.958
213	DC-442	A=A1AAAA1a2aaaa(a2)AAa(aa3)a(a34)AAa5a4aaaa5	OPLS-2005	9.128	[*]N1CCC1CCO	-5.679	-7.245	-33.465
214	DC-443	A=A1AAAA1a2aaaa(a2)AAa(aa3)a(a34)AAa5a4aaaa5	OPLS-2005	9.251	[*]c1c(C)cccn1	-5.655	-5.749	-43.013
215	DC-444	A=A1AAAA1a2aaaa(a2)AAa(aa3)a(a34)AAa5a4aaaa5	OPLS-2005	9.481	[*]N1[C@@H](C)CC1(C)C	-5.655	-7.368	-35.498
216	DC-445	A=A1AAAA1a2aaaa(a2)AAa(aa3)a(a34)AAa5a4aaaa5	OPLS-2005	9.050	[*]NC1(C)CCCC1	-5.638	-7.954	-52.006
217	DC-446	A=A1AAAA1a2aaaa(a2)AAa(aa3)a(a34)AAa5a4aaaa5	OPLS-2005	9.107	[*]N(C)C1CC(C1)O	-5.638	-7.841	-52.280
218	DC-447	A=A1AAAA1a2aaaa(a2)AAa(aa3)a(a34)AAa5a4aaaa5	OPLS-2005	9.006	[*]c1c(=O)n(C)[nH]c1	-5.602	-5.968	-42.788

219	DC-448	A=A1AAAA1a2aaaa(a2)AAa(aa3)a(a34)AAa5a4aaaa5	OPLS-2005	9.030	[*]N(C1)CC1O	-5.600	-7.029	-42.498
220	DC-449	A=A1AAAA1a2aaaa(a2)AAa(aa3)a(a34)AAa5a4aaaa5	OPLS-2005	9.028	[*]CC1(C)[C@H](C1)O	-5.598	-5.755	-48.091
221	DC-450	A=A1AAAA1a2aaaa(a2)AAa(aa3)a(a34)AAa5a4aaaa5	OPLS-2005	9.114	[*]NC1(CC)CNC1	-5.590	-6.823	-29.032
222	DC-451	A=A1AAAA1a2aaaa(a2)AAa(aa3)a(a34)AAa5a4aaaa5	OPLS-2005	9.147	[*]N1CCC1C#N	-5.573	-6.421	-44.166
223	DC-452	A=A1AAAA1a2aaaa(a2)AAa(aa3)a(a34)AAa5a4aaaa5	OPLS-2005	9.028	[*]CC(C)CNC	-5.563	-7.447	-30.784
224	DC-453	A=A1AAAA1a2aaaa(a2)AAa(aa3)a(a34)AAa5a4aaaa5	OPLS-2005	9.199	[*]c1c(C)cn(C)c1	-5.560	-5.657	-40.168
225	DC-454	A=A1AAAA1a2aaaa(a2)AAa(aa3)a(a34)AAa5a4aaaa5	OPLS-2005	9.123	[*]N(C1)CC1C(C)C	-5.557	-5.949	-41.152
226	DC-455	A=A1AAAA1a2aaaa(a2)AAa(aa3)a(a34)AAa5a4aaaa5	OPLS-2005	9.065	[*]NC(C)CC	-5.513	-7.994	-53.690
227	DC-456	A=A1AAAA1a2aaaa(a2)AAa(aa3)a(a34)AAa5a4aaaa5	OPLS-2005	9.082	[*]c1cncn1C	-5.497	-8.122	-50.630
228	DC-457	A=A1AAAA1a2aaaa(a2)AAa(aa3)a(a34)AAa5a4aaaa5	OPLS-2005	9.060	[*]NC(C)(C)C	-5.488	-7.474	-46.348
229	DC-458	A=A1AAAA1a2aaaa(a2)AAa(aa3)a(a34)AAa5a4aaaa5	OPLS-2005	9.032	[*]CCC(C)(O)CN	-5.407	-6.444	-40.039
230	DC-459	A=A1AAAA1a2aaaa(a2)AAa(aa3)a(a34)AAa5a4aaaa5	OPLS-2005	9.232	[*]N1CCCOCC1	-5.404	-6.382	-34.740
231	DC-460	A=A1AAAA1a2aaaa(a2)AAa(aa3)a(a34)AAa5a4aaaa5	OPLS-2005	9.300	[*]N(C1)CCC1(C)C	-5.400	-8.100	-50.767
232	DC-461	A=A1AAAA1a2aaaa(a2)AAa(aa3)a(a34)AAa5a4aaaa5	OPLS-2005	9.029	[*]OC(CC)CC	-5.370	-7.854	-51.745
233	DC-462	A=A1AAAA1a2aaaa(a2)AAa(aa3)a(a34)AAa5a4aaaa5	OPLS-2005	9.291	[*]N1CC[C@@H]1CC	-5.364	-7.432	-34.520
234	DC-463	A=A1AAAA1a2aaaa(a2)AAa(aa3)a(a34)AAa5a4aaaa5	OPLS-2005	9.074	[*]n1nccc1CO	-5.363	-6.540	-40.121
235	DC-464	A=A1AAAA1a2aaaa(a2)AAa(aa3)a(a34)AAa5a4aaaa5	OPLS-2005	9.182	[*]N(C1)CC1(C)C	-5.348	-6.711	-47.611
236	DC-465	A=A1AAAA1a2aaaa(a2)AAa(aa3)a(a34)AAa5a4aaaa5	OPLS-2005	9.357	[*]N(C)C(C)(C)C	-5.336	-6.232	-30.545
237	DC-466	A=A1AAAA1a2aaaa(a2)AAa(aa3)a(a34)AAa5a4aaaa5	OPLS-2005	9.213	[*]c1c(C)coc1O	-5.324	-5.465	-44.572
238	DC-467	A=A1AAAA1a2aaaa(a2)AAa(aa3)a(a34)AAa5a4aaaa5	OPLS-2005	9.047	[*]N(C1)CC1N	-5.317	-7.522	-38.219
239	DC-468	A=A1AAAA1a2aaaa(a2)AAa(aa3)a(a34)AAa5a4aaaa5	OPLS-2005	9.058	[*]CCC1(CN)CC1	-5.313	-6.563	-42.133
240	DC-469	A=A1AAAA1a2aaaa(a2)AAa(aa3)a(a34)AAa5a4aaaa5	OPLS-2005	9.248	[*]N(C)C1CCCC1	-5.240	-6.357	-49.928
241	DC-470	A=A1AAAA1a2aaaa(a2)AAa(aa3)a(a34)AAa5a4aaaa5	OPLS-2005	9.015	[*]CC1(O)CN(C)C1	-5.238	-7.911	-48.770

242	DC-471	A=A1AAAA1a2aaaa(a2)AAa(aa3)a(a34)AAa5a4aaaa5	OPLS-2005	9.404	[*]N(C)[C@@H](C)C(C)C	-5.235	-5.577	-40.829
243	DC-472	A=A1AAAA1a2aaaa(a2)AAa(aa3)a(a34)AAa5a4aaaa5	OPLS-2005	9.062	[*]OC1(CC)CNC1	-5.223	-6.655	-28.789
244	DC-473	A=A1AAAA1a2aaaa(a2)AAa(aa3)a(a34)AAa5a4aaaa5	OPLS-2005	9.121	[*]N(C1)CC1(C)N	-5.221	-6.490	-41.481
245	DC-474	A=A1AAAA1a2aaaa(a2)AAa(aa3)a(a34)AAa5a4aaaa5	OPLS-2005	9.158	[*]N(C)CC(C)(C)O	-5.203	-5.531	-37.007
246	DC-475	A=A1AAAA1a2aaaa(a2)AAa(aa3)a(a34)AAa5a4aaaa5	OPLS-2005	9.090	[*]O[C@@H](C)C1CCC1	-5.195	-6.599	-32.198
247	DC-476	A=A1AAAA1a2aaaa(a2)AAa(aa3)a(a34)AAa5a4aaaa5	OPLS-2005	9.061	[*]O[C@@H](C1(C)C)CN1	-5.165	-6.551	-41.159
248	DC-477	A=A1AAAA1a2aaaa(a2)AAa(aa3)a(a34)AAa5a4aaaa5	OPLS-2005	9.026	[*]N(C)CC1CC1	-5.157	-6.194	-45.332
249	DC-478	A=A1AAAA1a2aaaa(a2)AAa(aa3)a(a34)AAa5a4aaaa5	OPLS-2005	9.453	[*]N1CCC1(C)CC	-5.130	-7.188	-46.314
250	DC-479	A=A1AAAA1a2aaaa(a2)AAa(aa3)a(a34)AAa5a4aaaa5	OPLS-2005	9.349	[*]c1c(C)cccc1	-5.126	-7.714	-27.110
251	DC-480	A=A1AAAA1a2aaaa(a2)AAa(aa3)a(a34)AAa5a4aaaa5	OPLS-2005	9.025	[*]c1c(O)occ1	-5.125	-5.260	-43.865
252	DC-481	A=A1AAAA1a2aaaa(a2)AAa(aa3)a(a34)AAa5a4aaaa5	OPLS-2005	9.001	[*]OCCC1(N)CC1	-5.122	-6.582	-46.579
253	DC-482	A=A1AAAA1a2aaaa(a2)AAa(aa3)a(a34)AAa5a4aaaa5	OPLS-2005	9.002	[*]c1nncn1CC	-5.121	-5.225	-27.297
254	DC-483	A=A1AAAA1a2aaaa(a2)AAa(aa3)a(a34)AAa5a4aaaa5	OPLS-2005	9.030	[*]N(C1)CC1O	-5.115	-7.673	-28.707
255	DC-484	A=A1AAAA1a2aaaa(a2)AAa(aa3)a(a34)AAa5a4aaaa5	OPLS-2005	9.112	[*]CC1(CN)CNC1	-5.110	-5.220	-20.438
256	DC-485	A=A1AAAA1a2aaaa(a2)AAa(aa3)a(a34)AAa5a4aaaa5	OPLS-2005	9.001	[*]c1c(O)c[nH]n1	-5.071	-5.687	-33.458
257	DC-486	A=A1AAAA1a2aaaa(a2)AAa(aa3)a(a34)AAa5a4aaaa5	OPLS-2005	9.047	[*]n(c1)ccc1CN	-5.059	-5.151	-40.007
258	DC-487	A=A1AAAA1a2aaaa(a2)AAa(aa3)a(a34)AAa5a4aaaa5	OPLS-2005	9.146	[*]CC1(C)[C@H](O)CC1	-5.048	-6.276	-43.058
259	DC-488	A=A1AAAA1a2aaaa(a2)AAa(aa3)a(a34)AAa5a4aaaa5	OPLS-2005	9.212	[*]N(C1)CC1(C)CC	-5.023	-6.332	-5.559
260	DC-489	A=A1AAAA1a2aaaa(a2)AAa(aa3)a(a34)AAa5a4aaaa5	OPLS-2005	9.204	[*]N(C1)CC[C@@H]1CN	-4.994	-6.226	-38.338
261	DC-490	A=A1AAAA1a2aaaa(a2)AAa(aa3)a(a34)AAa5a4aaaa5	OPLS-2005	9.087	[*]C[C@@H](CO)C1CC1	-4.984	-6.187	-26.834
262	DC-491	A=A1AAAA1a2aaaa(a2)AAa(aa3)a(a34)AAa5a4aaaa5	OPLS-2005	9.116	[*]N(C1)CC1CC	-4.958	-5.995	-44.006
263	DC-492	A=A1AAAA1a2aaaa(a2)AAa(aa3)a(a34)AAa5a4aaaa5	OPLS-2005	9.233	[*]N(C)CC(C)C	-4.939	-6.942	-13.982
264	DC-493	A=A1AAAA1a2aaaa(a2)AAa(aa3)a(a34)AAa5a4aaaa5	OPLS-2005	9.232	[*]N1CCCOCC1	-4.930	-5.214	-37.385

265	DC-494	A=A1AAAA1a2aaaa(a2)AAa(aa3)a(a34)AAa5a4aaaa5	OPLS-2005	9.026	[*]N(C)CC1CC1	-4.924	-6.015	-26.619
266	DC-495	A=A1AAAA1a2aaaa(a2)AAa(aa3)a(a34)AAa5a4aaaa5	OPLS-2005	9.204	[*]N(C1)CC[C@@H]1CN	-4.918	-6.493	-45.514
267	DC-496	A=A1AAAA1a2aaaa(a2)AAa(aa3)a(a34)AAa5a4aaaa5	OPLS-2005	9.148	[*]c1c(N)cccn1	-4.875	-6.061	-45.498
268	DC-497	A=A1AAAA1a2aaaa(a2)AAa(aa3)a(a34)AAa5a4aaaa5	OPLS-2005	9.055	[*]N(C)C1CC1	-4.869	-6.863	-34.921
269	DC-498	A=A1AAAA1a2aaaa(a2)AAa(aa3)a(a34)AAa5a4aaaa5	OPLS-2005	9.481	[*]N1[C@@H](C)CC1(C)C	-4.856	-5.146	-37.889
270	DC-499	A=A1AAAA1a2aaaa(a2)AAa(aa3)a(a34)AAa5a4aaaa5	OPLS-2005	9.103	[*]c1nc(C)cn1C	-4.806	-6.731	-44.544
271	DC-500	A=A1AAAA1a2aaaa(a2)AAa(aa3)a(a34)AAa5a4aaaa5	OPLS-2005	9.023	[*]CNC1(CN)CC1	-4.787	-6.121	-20.954
272	DC-501	A=A1AAAA1a2aaaa(a2)AAa(aa3)a(a34)AAa5a4aaaa5	OPLS-2005	9.082	[*]N[C@@H](C)C1CCC1	-4.780	-5.942	-46.130
273	DC-502	A=A1AAAA1a2aaaa(a2)AAa(aa3)a(a34)AAa5a4aaaa5	OPLS-2005	9.082	[*]c1cncn1C	-4.731	-6.266	-40.670
274	DC-503	A=A1AAAA1a2aaaa(a2)AAa(aa3)a(a34)AAa5a4aaaa5	OPLS-2005	9.356	[*]NC(C)(C)C(C)C	-4.718	-6.231	-47.081
275	DC-504	A=A1AAAA1a2aaaa(a2)AAa(aa3)a(a34)AAa5a4aaaa5	OPLS-2005	9.201	[*]N1C[C@@H](N)C1(C)C	-4.712	-4.837	-23.645
276	DC-505	A=A1AAAA1a2aaaa(a2)AAa(aa3)a(a34)AAa5a4aaaa5	OPLS-2005	9.012	[*]c1cc(CN)oc1	-4.631	-4.746	-36.220
277	DC-506	A=A1AAAA1a2aaaa(a2)AAa(aa3)a(a34)AAa5a4aaaa5	OPLS-2005	9.116	[*]c1ccc([nH]1)CC	-4.612	-7.064	-45.003
278	DC-507	A=A1AAAA1a2aaaa(a2)AAa(aa3)a(a34)AAa5a4aaaa5	OPLS-2005	9.102	[*]OC1([C@H](C)N)CC1	-4.584	-5.128	-27.433
279	DC-508	A=A1AAAA1a2aaaa(a2)AAa(aa3)a(a34)AAa5a4aaaa5	OPLS-2005	9.033	[*]N(C1)CC1F	-4.568	-5.510	-21.381
280	DC-509	A=A1AAAA1a2aaaa(a2)AAa(aa3)a(a34)AAa5a4aaaa5	OPLS-2005	9.082	[*]N(C1)CC1(C)O	-4.565	-6.085	-35.856
281	DC-510	A=A1AAAA1a2aaaa(a2)AAa(aa3)a(a34)AAa5a4aaaa5	OPLS-2005	9.091	[*]CN(C)C(C)(C)C	-4.538	-6.037	-48.442
282	DC-511	A=A1AAAA1a2aaaa(a2)AAa(aa3)a(a34)AAa5a4aaaa5	OPLS-2005	9.155	CCN([*])CC	-4.488	-6.058	-35.418
283	DC-512	A=A1AAAA1a2aaaa(a2)AAa(aa3)a(a34)AAa5a4aaaa5	OPLS-2005	9.082	[*]c1ncc(N)n1C	-4.485	-4.591	-41.311
284	DC-513	A=A1AAAA1a2aaaa(a2)AAa(aa3)a(a34)AAa5a4aaaa5	OPLS-2005	9.066	[*]OCC1(C)CCC1	-4.462	-4.686	-2.719
285	DC-514	A=A1AAAA1a2aaaa(a2)AAa(aa3)a(a34)AAa5a4aaaa5	OPLS-2005	9.072	[*]O[C@@H](C)C1(N)CC1	-4.427	-4.981	-31.992
286	DC-515	A=A1AAAA1a2aaaa(a2)AAa(aa3)a(a34)AAa5a4aaaa5	OPLS-2005	9.005	[*][N-]C1CCNC1	-4.419	-5.520	-35.301
287	DC-516	A=A1AAAA1a2aaaa(a2)AAa(aa3)a(a34)AAa5a4aaaa5	OPLS-2005	9.175	[*]CC1(C)[C@H](N)CC1	-4.412	-5.622	-36.798

288	DC-517	A=A1AAAA1a2aaaa(a2)AAa(aa3)a(a34)AAa5a4aaaa5	OPLS-2005	9.068	[*]N(C1)CC1C	-4.379	-5.693	-46.268
289	DC-518	A=A1AAAA1a2aaaa(a2)AAa(aa3)a(a34)AAa5a4aaaa5	OPLS-2005	9.116	[*]CCC(N)C1CC1	-4.314	-5.942	-40.297
290	DC-519	A=A1AAAA1a2aaaa(a2)AAa(aa3)a(a34)AAa5a4aaaa5	OPLS-2005	9.068	[*]N(C1)CC1C	-4.294	-5.213	-13.475
291	DC-520	A=A1AAAA1a2aaaa(a2)AAa(aa3)a(a34)AAa5a4aaaa5	OPLS-2005	9.063	[*]c1c(O)ccs1	-4.291	-5.907	-43.644
292	DC-521	A=A1AAAA1a2aaaa(a2)AAa(aa3)a(a34)AAa5a4aaaa5	OPLS-2005	9.112	[*]OC1(CN)CCC1	-4.281	-6.200	-17.688
293	DC-522	A=A1AAAA1a2aaaa(a2)AAa(aa3)a(a34)AAa5a4aaaa5	OPLS-2005	9.157	O=C1N([*])CCCN1	-4.276	-4.890	-40.115
294	DC-523	A=A1AAAA1a2aaaa(a2)AAa(aa3)a(a34)AAa5a4aaaa5	OPLS-2005	9.106	[*]N[C@@H](C1(C)C)CN1	-4.261	-5.609	-22.303
295	DC-524	A=A1AAAA1a2aaaa(a2)AAa(aa3)a(a34)AAa5a4aaaa5	OPLS-2005	9.059	[*]CC1(O)C[C@H](C)C1	-4.255	-4.618	-40.517
296	DC-525	A=A1AAAA1a2aaaa(a2)AAa(aa3)a(a34)AAa5a4aaaa5	OPLS-2005	9.367	[*]N(C)C(C)(C)CN	-4.245	-5.449	-37.072
297	DC-526	A=A1AAAA1a2aaaa(a2)AAa(aa3)a(a34)AAa5a4aaaa5	OPLS-2005	9.216	CCN([*])C1CC1	-4.227	-5.195	-31.644
298	DC-527	A=A1AAAA1a2aaaa(a2)AAa(aa3)a(a34)AAa5a4aaaa5	OPLS-2005	9.184	[*]c1ccc(N)cc1	-4.175	-6.426	-46.554
299	DC-528	A=A1AAAA1a2aaaa(a2)AAa(aa3)a(a34)AAa5a4aaaa5	OPLS-2005	9.255	C1CN([*])CCC1O	-4.167	-5.330	-40.711
300	DC-529	A=A1AAAA1a2aaaa(a2)AAa(aa3)a(a34)AAa5a4aaaa5	OPLS-2005	9.282	[*]N(C1)CCCC1O	-4.094	-6.812	-0.619
301	DC-530	A=A1AAAA1a2aaaa(a2)AAa(aa3)a(a34)AAa5a4aaaa5	OPLS-2005	9.182	[*]CC(C)(O)C(C)C	-4.091	-4.932	-19.866
302	DC-531	A=A1AAAA1a2aaaa(a2)AAa(aa3)a(a34)AAa5a4aaaa5	OPLS-2005	9.090	[*]CC(C)(N)[C@H](C)O	-4.072	-6.111	-29.755
303	DC-532	A=A1AAAA1a2aaaa(a2)AAa(aa3)a(a34)AAa5a4aaaa5	OPLS-2005	9.033	[*]OCC1(CC1)NC	-4.071	-6.214	-49.232
304	DC-533	A=A1AAAA1a2aaaa(a2)AAa(aa3)a(a34)AAa5a4aaaa5	OPLS-2005	9.477	CCN([*])C(C)(C)C	-4.039	-4.322	-41.558
305	DC-534	A=A1AAAA1a2aaaa(a2)AAa(aa3)a(a34)AAa5a4aaaa5	OPLS-2005	9.253	[*]N(C)CC1(N)CC1	-4.027	-6.296	-19.951
306	DC-535	A=A1AAAA1a2aaaa(a2)AAa(aa3)a(a34)AAa5a4aaaa5	OPLS-2005	9.011	[*]NC(C)C1CC1	-4.012	-4.404	-34.721
307	DC-536	A=A1AAAA1a2aaaa(a2)AAa(aa3)a(a34)AAa5a4aaaa5	OPLS-2005	9.095	[*]c1c(C)coc1	-4.010	-5.847	-38.845
308	DC-537	A=A1AAAA1a2aaaa(a2)AAa(aa3)a(a34)AAa5a4aaaa5	OPLS-2005	9.112	[*]c1cncn1CC	-3.996	-6.560	-37.832
309	DC-538	A=A1AAAA1a2aaaa(a2)AAa(aa3)a(a34)AAa5a4aaaa5	OPLS-2005	9.074	[*]c1c(O)cc[nH]1	-3.989	-4.095	-42.019
310	DC-539	A=A1AAAA1a2aaaa(a2)AAa(aa3)a(a34)AAa5a4aaaa5	OPLS-2005	9.060	[*]NC(C)(C)C	-3.987	-5.489	-37.663

311	DC-540	A=A1AAAA1a2aaaa(a2)AAa(aa3)a(a34)AAa5a4aaaa5	OPLS-2005	9.317	[*]N1CC[C@@H]1CCC	-3.975	-4.952	-37.701
312	DC-541	A=A1AAAA1a2aaaa(a2)AAa(aa3)a(a34)AAa5a4aaaa5	OPLS-2005	9.148	[*]CC(C)(CN)NC	-3.958	-6.676	-21.611
313	DC-542	A=A1AAAA1a2aaaa(a2)AAa(aa3)a(a34)AAa5a4aaaa5	OPLS-2005	9.108	[*]N(C)[C@@H]1C[C@@H](C1)N	-3.883	-5.896	-43.246
314	DC-543	A=A1AAAA1a2aaaa(a2)AAa(aa3)a(a34)AAa5a4aaaa5	OPLS-2005	9.201	[*]N1C[C@@H](N)C1(C)C	-3.868	-5.799	-38.128
315	DC-544	A=A1AAAA1a2aaaa(a2)AAa(aa3)a(a34)AAa5a4aaaa5	OPLS-2005	9.052	[*]CC(N)C(C)(C)C	-3.853	-5.030	-37.132
316	DC-545	A=A1AAAA1a2aaaa(a2)AAa(aa3)a(a34)AAa5a4aaaa5	OPLS-2005	9.151	[*]c1c(C)onc1O	-3.837	-5.029	-45.446
317	DC-546	A=A1AAAA1a2aaaa(a2)AAa(aa3)a(a34)AAa5a4aaaa5	OPLS-2005	9.104	[*]N(C1)CC1N(C)C	-3.782	-5.105	-41.108
318	DC-547	A=A1AAAA1a2aaaa(a2)AAa(aa3)a(a34)AAa5a4aaaa5	OPLS-2005	9.137	[*]CN(C)C(C)CN	-3.779	-6.375	-40.488
319	DC-548	A=A1AAAA1a2aaaa(a2)AAa(aa3)a(a34)AAa5a4aaaa5	OPLS-2005	9.367	[*]N(C)C(C)(C)CN	-3.709	-5.115	-45.516
320	DC-549	A=A1AAAA1a2aaaa(a2)AAa(aa3)a(a34)AAa5a4aaaa5	OPLS-2005	9.068	[*]N(C1)CC1C	-3.645	-5.655	-4.087
321	DC-550	A=A1AAAA1a2aaaa(a2)AAa(aa3)a(a34)AAa5a4aaaa5	OPLS-2005	9.140	[*]c1c(CN)occ1	-3.614	-4.819	-36.993
322	DC-551	A=A1AAAA1a2aaaa(a2)AAa(aa3)a(a34)AAa5a4aaaa5	OPLS-2005	9.166	[*]N(C1)CC1(F)CN	-3.578	-4.826	-22.881
323	DC-552	A=A1AAAA1a2aaaa(a2)AAa(aa3)a(a34)AAa5a4aaaa5	OPLS-2005	9.016	[*]CC1(C)CC1	-3.567	-5.092	-32.788
324	DC-553	A=A1AAAA1a2aaaa(a2)AAa(aa3)a(a34)AAa5a4aaaa5	OPLS-2005	9.171	[*]c1cc(C)ccn1	-3.545	-6.231	-43.928
325	DC-554	A=A1AAAA1a2aaaa(a2)AAa(aa3)a(a34)AAa5a4aaaa5	OPLS-2005	9.054	[*]CC1(C)CNC1	-3.529	-5.844	-25.033
326	DC-555	A=A1AAAA1a2aaaa(a2)AAa(aa3)a(a34)AAa5a4aaaa5	OPLS-2005	9.064	[*]CC(C)(O)CCC	-3.525	-5.204	-40.857
327	DC-556	A=A1AAAA1a2aaaa(a2)AAa(aa3)a(a34)AAa5a4aaaa5	OPLS-2005	9.123	[*]c1ncc(C)n1C	-3.458	-5.848	-44.721
328	DC-557	A=A1AAAA1a2aaaa(a2)AAa(aa3)a(a34)AAa5a4aaaa5	OPLS-2005	9.188	[*]CC1(C)[C@H](C)NC1	-3.452	-3.553	-13.382
329	DC-558	A=A1AAAA1a2aaaa(a2)AAa(aa3)a(a34)AAa5a4aaaa5	OPLS-2005	9.148	[*]c1c(C)[nH]cc1	-3.304	-5.756	-42.387
330	DC-559	A=A1AAAA1a2aaaa(a2)AAa(aa3)a(a34)AAa5a4aaaa5	OPLS-2005	9.023	[*]CC(F)(C)C	-3.300	-4.764	-36.981
331	DC-560	A=A1AAAA1a2aaaa(a2)AAa(aa3)a(a34)AAa5a4aaaa5	OPLS-2005	9.109	[*]CC1(C#N)CCC1	-3.293	-3.390	-34.178
332	DC-561	A=A1AAAA1a2aaaa(a2)AAa(aa3)a(a34)AAa5a4aaaa5	OPLS-2005	9.512	[*]N1CCCC1(C)C	-3.262	-5.312	-35.733
333	DC-562	A=A1AAAA1a2aaaa(a2)AAa(aa3)a(a34)AAa5a4aaaa5	OPLS-2005	9.073	[*]CCC(C)(N)CO	-3.157	-5.738	-49.405

334	DC-563	A=A1AAAA1a2aaaa(a2)AAa(aa3)a(a34)AAa5a4aaaa5	OPLS-2005	9.072	[*]O[C@@H](C)C1(N)CC1	-3.130	-4.834	-35.808
335	DC-564	A=A1AAAA1a2aaaa(a2)AAa(aa3)a(a34)AAa5a4aaaa5	OPLS-2005	9.029	n1cn([*])ccc1=O	-3.126	-5.299	-42.665
336	DC-565	A=A1AAAA1a2aaaa(a2)AAa(aa3)a(a34)AAa5a4aaaa5	OPLS-2005	9.078	[*]c1c(O)cn(n1)C	-3.113	-5.220	-42.910
337	DC-566	A=A1AAAA1a2aaaa(a2)AAa(aa3)a(a34)AAa5a4aaaa5	OPLS-2005	9.230	O1CCN([*])C[C@@H]1C	-3.094	-5.344	-42.408
338	DC-567	A=A1AAAA1a2aaaa(a2)AAa(aa3)a(a34)AAa5a4aaaa5	OPLS-2005	9.046	[*]N(C)CC(F)F	-3.091	-5.411	-14.076
339	DC-568	A=A1AAAA1a2aaaa(a2)AAa(aa3)a(a34)AAa5a4aaaa5	OPLS-2005	9.185	[*]N(C1)CC1(C)OC	-3.009	-5.013	-38.776
340	DC-569	A=A1AAAA1a2aaaa(a2)AAa(aa3)a(a34)AAa5a4aaaa5	OPLS-2005	9.106	[*]N[C@@H](C1(C)C)CN1	-2.980	-5.151	-17.909
341	DC-570	A=A1AAAA1a2aaaa(a2)AAa(aa3)a(a34)AAa5a4aaaa5	OPLS-2005	9.214	[*]c1c(F)cccc1	-2.900	-5.488	-40.448
342	DC-571	A=A1AAAA1a2aaaa(a2)AAa(aa3)a(a34)AAa5a4aaaa5	OPLS-2005	9.112	[*]c1cncn1CC	-2.890	-5.403	-43.348
343	DC-572	A=A1AAAA1a2aaaa(a2)AAa(aa3)a(a34)AAa5a4aaaa5	OPLS-2005	9.090	[*]c1cc(F)cnc1	-2.835	-5.521	-37.054
344	DC-573	A=A1AAAA1a2aaaa(a2)AAa(aa3)a(a34)AAa5a4aaaa5	OPLS-2005	9.146	[*]CC(CO)N(C)C	-2.815	-4.519	-12.806
345	DC-574	A=A1AAAA1a2aaaa(a2)AAa(aa3)a(a34)AAa5a4aaaa5	OPLS-2005	9.050	[*]c1cc(CO)c[nH]1	-2.735	-5.187	-32.366
346	DC-575	A=A1AAAA1a2aaaa(a2)AAa(aa3)a(a34)AAa5a4aaaa5	OPLS-2005	9.254	[*]c1c(C)nccc1	-2.725	-5.411	-41.555
347	DC-576	A=A1AAAA1a2aaaa(a2)AAa(aa3)a(a34)AAa5a4aaaa5	OPLS-2005	9.137	[*]N(C1)CC1(N)CC	-2.640	-5.294	-41.693
348	DC-577	A=A1AAAA1a2aaaa(a2)AAa(aa3)a(a34)AAa5a4aaaa5	OPLS-2005	9.030	[*]CN1CCNCC1	-2.629	-4.960	-42.710
349	DC-578	A=A1AAAA1a2aaaa(a2)AAa(aa3)a(a34)AAa5a4aaaa5	OPLS-2005	9.201	[*]c1cc(C#N)ccc1	-2.628	-5.217	-41.620
350	DC-579	A=A1AAAA1a2aaaa(a2)AAa(aa3)a(a34)AAa5a4aaaa5	OPLS-2005	9.039	[*]CC1(O)CCC1	-2.601	-4.222	-35.527
351	DC-580	A=A1AAAA1a2aaaa(a2)AAa(aa3)a(a34)AAa5a4aaaa5	OPLS-2005	9.287	[*]N(C)[C@@H](C1)C1(C)C	-2.549	-3.629	-31.452
352	DC-581	A=A1AAAA1a2aaaa(a2)AAa(aa3)a(a34)AAa5a4aaaa5	OPLS-2005	9.015	[*]NCC(C)(C)CO	-2.543	-3.456	-35.856
353	DC-582	A=A1AAAA1a2aaaa(a2)AAa(aa3)a(a34)AAa5a4aaaa5	OPLS-2005	9.153	[*]CC1(C)CCNC1	-2.539	-4.645	-34.009
354	DC-583	A=A1AAAA1a2aaaa(a2)AAa(aa3)a(a34)AAa5a4aaaa5	OPLS-2005	9.103	[*]c1nc(C)cn1C	-2.476	-4.656	-16.837
355	DC-584	A=A1AAAA1a2aaaa(a2)AAa(aa3)a(a34)AAa5a4aaaa5	OPLS-2005	9.200	[*]c1ccc(C)cc1	-2.431	-4.449	-32.816
356	DC-585	A=A1AAAA1a2aaaa(a2)AAa(aa3)a(a34)AAa5a4aaaa5	OPLS-2005	9.074	[*]c1ccc([nH]1)CN	-2.329	-4.782	-34.931

357	DC-586	A=A1AAAA1a2aaaa(a2)AAa(aa3)a(a34)AAa5a4aaaa5	OPLS-2005	9.078	[*]c1ncc([nH]1)CC	-2.285	-4.852	-40.038
358	DC-587	A=A1AAAA1a2aaaa(a2)AAa(aa3)a(a34)AAa5a4aaaa5	OPLS-2005	9.261	[*]c1c(C)ccn1C	-2.185	-4.638	-39.112
359	DC-588	A=A1AAAA1a2aaaa(a2)AAa(aa3)a(a34)AAa5a4aaaa5	OPLS-2005	9.011	[*]NC(C)C1CC1	-2.138	-4.203	-35.436
360	DC-589	A=A1AAAA1a2aaaa(a2)AAa(aa3)a(a34)AAa5a4aaaa5	OPLS-2005	9.094	[*]c1c(C#N)[nH]cc1	-2.131	-4.610	-38.747
361	DC-590	A=A1AAAA1a2aaaa(a2)AAa(aa3)a(a34)AAa5a4aaaa5	OPLS-2005	9.244	[*]c1c(N)ccn1C	-2.098	-4.472	-38.093
362	DC-591	A=A1AAAA1a2aaaa(a2)AAa(aa3)a(a34)AAa5a4aaaa5	OPLS-2005	9.104	[*]N(C1)CC1N(C)C	-2.067	-4.721	-18.919
363	DC-592	A=A1AAAA1a2aaaa(a2)AAa(aa3)a(a34)AAa5a4aaaa5	OPLS-2005	9.023	[*]CNC1(CN)CC1	-2.011	-4.325	-17.338
364	DC-593	A=A1AAAA1a2aaaa(a2)AAa(aa3)a(a34)AAa5a4aaaa5	OPLS-2005	9.073	[*]CCC(C)(N)CO	-1.975	-4.499	-35.088
365	DC-594	A=A1AAAA1a2aaaa(a2)AAa(aa3)a(a34)AAa5a4aaaa5	OPLS-2005	9.195	[*]c1ccc(C#N)cc1	-1.863	-4.451	-31.777
366	DC-595	A=A1AAAA1a2aaaa(a2)AAa(aa3)a(a34)AAa5a4aaaa5	OPLS-2005	9.172	[*]CC1(C)C(C)(C)C1	-1.819	-4.382	-38.187
367	DC-596	A=A1AAAA1a2aaaa(a2)AAa(aa3)a(a34)AAa5a4aaaa5	OPLS-2005	9.067	[*]NC(C)(C)CO	-1.761	-3.135	-31.918
368	DC-597	A=A1AAAA1a2aaaa(a2)AAa(aa3)a(a34)AAa5a4aaaa5	OPLS-2005	9.090	[*]CC(C)(N)[C@H](C)O	-1.699	-3.521	-31.922
369	DC-598	A=A1AAAA1a2aaaa(a2)AAa(aa3)a(a34)AAa5a4aaaa5	OPLS-2005	9.229	[*]c1cccn1CC	-1.673	-4.126	-35.345
370	DC-599	A=A1AAAA1a2aaaa(a2)AAa(aa3)a(a34)AAa5a4aaaa5	OPLS-2005	9.314	[*]N(C1)CCC[C@@H]1C	-1.531	-3.029	-28.160
371	DC-600	A=A1AAAA1a2aaaa(a2)AAa(aa3)a(a34)AAa5a4aaaa5	OPLS-2005	9.357	[*]N(C)C(C)(C)C	-0.801	-2.304	-31.399

Table S5: The specific residues involved in the receptor-ligand interaction of reference and designed compounds using molecular docking analysis.

S.NO	Active Ligands	Hydrophobic Interactions				Hydrogen Bond Interactions			Total number of Interactions
		Alkyl-Alkyl and Pi-Alkyl	Pi-Pi	Pi-sigma	Pi-Sulfur	Classical	Non-Classical (Carbon-Hydrogen)	Halogen	
1	RC-01	ARG-109, ALA-111, ARG-119, TRP-124, ILE-128, ILE-130, VAL-255, MET-259, VAL-281, MET-291	TRP-124, TRP-267	Nil	Nil	LEU-120, ILE-128	SER-278, SER-287	VAL-255	23
2	RC-02	ARG-109, ALA-111, TRP-124, ILE-128, ILE-130, VAL-255, MET-259, VAL-281, MET-291	TRP-124	Nil	Nil	LEU-120, ILE-128	ARG-119, SER-287	VAL-255	18
3	DC-01	ARG-109, ALA-111, ILE-113, ILE-117, ARG-119, TRP-124, ILE-128, ILE-130, VAL-255, MET-259, TRP-267, MET-290, MET-291	TRP-124	Nil	Nil	ILE-128	PRO-118, VAL-255	ILE-117	23
4	DC-02	ARG-109, ALA-111, ILE-113, ILE-117, ARR-119, TRP-124, ILE-128, ILE-130, VAL-255, MET-259, MET-290, MET-291	TRP-124, TRP-267			ILE-112, ILE-128		VAL-255	22
5	DC-03	ARG-109, ALA-111, ILE-113, ILE-117, ARF-119, TRP-124, ILE-128, ILE-130, VAL-255, MET-259, MET-290, MET-291	TRP-124	Nil	Nil	ILE-128	PRO-118, VAL-255	VAL-255	25
6	DC-04	ARG-109, ALA-111, ILE-112, ILE-117, TRP-124, ILE-128, ILE-130, VAL-255, MET-259, TYR-285, MET-	TRP-124	Nil	Nil	ILE-128	Nil	VAL-255	20

		290, MET-291, CYC-379							
